# Cell Lineages and the Logic of Proliferative Control

**DOI:** 10.1371/journal.pbio.1000015

**Published:** 2009-01-20

**Authors:** Arthur D Lander, Kimberly K Gokoffski, Frederic Y. M Wan, Qing Nie, Anne L Calof

**Affiliations:** 1 Department of Developmental and Cell Biology, University of California, Irvine, Irvine, California, United States of America; 2 Biomedical Engineering, University of California, Irvine, Irvine, California, United States of America; 3 Center for Complex Biological Systems, University of California, Irvine, Irvine, California, United States of America; 4 Anatomy and Neurobiology, University of California, Irvine, Irvine, California, United States of America; 5 Mathematics, University of California, Irvine, Irvine, California, United States of America; Salk Institute for Biological Studies, United States of America

## Abstract

It is widely accepted that the growth and regeneration of tissues and organs is tightly controlled. Although experimental studies are beginning to reveal molecular mechanisms underlying such control, there is still very little known about the control strategies themselves. Here, we consider how secreted negative feedback factors (“chalones”) may be used to control the output of multistage cell lineages, as exemplified by the actions of GDF11 and activin in a self-renewing neural tissue, the mammalian olfactory epithelium (OE). We begin by specifying performance objectives—what, precisely, is being controlled, and to what degree—and go on to calculate how well different types of feedback configurations, feedback sensitivities, and tissue architectures achieve control. Ultimately, we show that many features of the OE—the number of feedback loops, the cellular processes targeted by feedback, even the location of progenitor cells within the tissue—fit with expectations for the best possible control. In so doing, we also show that certain distinctions that are commonly drawn among cells and molecules—such as whether a cell is a stem cell or transit-amplifying cell, or whether a molecule is a growth inhibitor or stimulator—may be the consequences of control, and not a reflection of intrinsic differences in cellular or molecular character.

## Author Summary

Many tissues and organs grow to precise sizes and, when injured, regenerate accurately and rapidly. Here, we ask whether the organization of cells into lineages, and the feedback interactions that occur within lineages, are necessary elements of control strategies that make such behavior possible. Drawing on mathematical modeling and the results of experimental manipulation of the mouse olfactory epithelium, we show that performance objectives, such as robust size specification, fast regeneration from a variety of initial conditions, and maintenance of high ratios of differentiated to undifferentiated cells, can be simultaneously achieved through a combination of lineage structures, signaling mechanisms, and spatial distributions of cell types that correspond well with what is observed in many growing and regenerating tissues. Key to successful control is an integral-feedback mechanism that is implemented when terminally differentiated cells secrete molecules that lower the probability that progenitor cells replicate versus differentiate. Interestingly, this mechanism also explains how the distinctive proliferative behaviors of stem cell and “transit-amplifying” cell populations can emerge as a consequence of feedback effects, rather than intrinsic programming of cell types.

## Abstract 3

Are common, generic strategies used in the quantitative control of tissue growth and regeneration? An investigation of feedback effects in multistage lineages suggests they are.

## Introduction

In recent decades, biologists have come to view cell lineages as fundamental units of tissue and organ development, maintenance, and regeneration. The highly differentiated, often nondividing cells that characterize the mature functions of tissues are seen as end products of orderly, tissue-specific sequences of cell divisions, during which progenitor cells pass through distinct stages, marked by expression of stage-specific genes (e.g., [1–4]). At the starting points of lineages—particularly those in self-renewing tissues such as blood, epidermis, and the intestinal lining—one finds stem cells, characterized both by multipotency (ability to produce many cell types) and their ability to maintain their own numbers through self-replication [5–8]. As scientists and clinicians have become increasingly interested in harnessing these features of stem cells to repair injury and cure disease, there has been a resurgence of interest in the mechanisms underlying the execution and regulation of cell lineages (e.g., [9–12]).

The functions of lineages are often presented in terms of progressive allocation of developmental potential: Thus, pluripotent stem cells often give rise to oligopotent progenitors, which in turn give rise to unipotent (committed) progenitors. The sequential expression of marker genes at different lineage stages may be related to transcriptional “priming” events needed to lock cells into specific patterns of gene expression [13,14].

Not all lineage stages correlate with restriction of cell fate, however, raising the question of what else lineages do. The fact that lineage intermediates often display “transit-amplifying” behavior, i.e., are capable of at least some degree of self-replication, has led to the suggestion that lineage stages play essential roles in the control of tissue and organ growth (with growth referring in this case to increase in cell number). Here, we seek to discover what those roles are. We approach this question from the perspective of lineages in general, and within the context of the mammalian olfactory epithelium (OE), the neural tissue that senses odor and transmits olfactory information to the brain. The OE is a continually self-renewing tissue, even in man, and is capable of rapid regeneration [15]. As discussed below, a wealth of experimental data on the OE lineage and the molecules that regulate it makes the OE an attractive system in which to investigate the relationship between lineages and growth control.

### Performance Objectives of Growing Tissues

In biology, “control” is often used interchangeably with “regulation,” but in engineering, control has a precise meaning: It refers to the strategies that enable a system to achieve desired ends, usually in a robust manner. To begin talking about the control needs of growing tissues and organs, we must first ask what are the “desired” ends, and to what kinds of uncertainties and perturbations must growth and differentiation be robust?

Perhaps the most obvious objective of a growth control system is to reach and maintain a specified size. Sizes of organs such as the brain, for example, are genetically specified within narrow tolerances (e.g., [16]). Moreover, self-renewing organs, such as the liver, seem to “remember” their appropriate sizes, as they accurately regenerate to their original sizes following even massive lesions [17]. The fact that many genetic alterations can affect final organ size (e.g., [18,19]) suggests that there are diverse molecular pathways by which size may be regulated.

A less obvious performance objective is control of growth rate. Consider, for example, a self-renewing tissue that maintains constant size by balancing continual cell death with cell production. Following an injury in which differentiated cells are destroyed, if there is no adjustment in cell production, those cells will be replaced only at the same (often very slow) rate at which they previously turned over. In regenerating tissues, however, it is common to observe a dramatic increase in proliferation following injuries, with rapid restoration of tissue morphology and size [17,20,21]. Even in tissues that do not regenerate, control of growth rate is likely to be important during development, so that the changing sizes of different organs are properly coordinated with each other.

Other possible targets of control are the proportions of cell types in a tissue. For example, in a branched lineage (one with more than one terminal-stage cell type) a fixed ratio of end products may be important for tissue or organ function [22]. In lineages that operate continuously, it may also be desirable to ensure that stem and progenitor cells (which do not usually contribute directly to tissue function) are not too great a fraction of the tissue mass.

How difficult should it be for tissues to achieve such objectives? With control, the difficulty of the task depends upon the magnitude of the perturbations that are normally encountered (e.g., genetic and/or random effects on cell behavior, environmental fluctuations, injury, and disease); the sensitivity of the system's behavior to those perturbations; and the level of imprecision in output that is acceptable.

**Figure 1:**
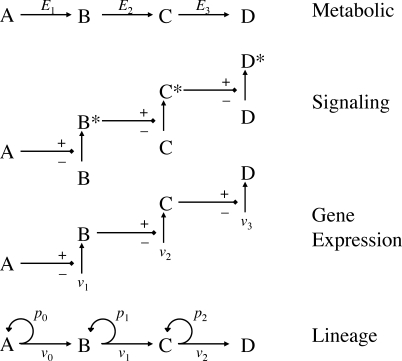
Biological Pathways That Are Potential Targets of Control Like metabolic, signaling, and gene expression pathways, cell lineages may be viewed as input–output pathways in which information or material flows through a series of defined elements (A–D) at rates controlled by measurable parameters (e.g., enzyme levels *E*_1_, *E*_2_, synthesis rates *v*_1_, *v*_2_, etc.). Unlike these other pathways, cell lineages are characterized by a potential for exponential expansion at most or all stages (parameters *p*_0_, *p*_1_, etc.). The impact of this difference on the strategies that may be used for tissue growth control has been little studied.

In recent years, increasing attention has been focused on the control challenges of biological networks, including those associated with metabolism, intracellular signaling, and gene regulation (e.g., [23–26]). Superficially, cell lineages look a great deal like these other kinds of pathways (Figure 1). Yet the components of lineages—cell stages—do not just transmit signals or material from one to another; they typically undergo autonomous, exponential expansion at the same time. This imparts a characteristic volatility to lineage dynamics that no doubt poses challenges for control. Given such challenges, it would not be surprising if the control of tissue and organ growth necessitates control strategies unlike those encountered elsewhere in biology. Here, we take steps toward identifying such strategies.

## Results

### Lineage Dynamics in the Absence of Control

**Figure 2:**
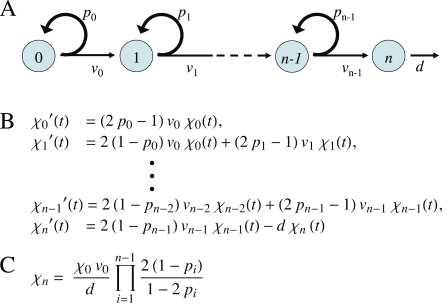
Lineage Behavior in the Absence of Control (A) Cartoon of an unbranched lineage that begins with a stem cell (type 0), progresses through an arbitrary number of transit-amplifying stages (types 1 to *n* − 1) and ends with a postmitotic terminal-stage cell (type *n*). Parameters *v*_i_ and *p*_i_ are the rate constants of cell cycle progression and the replication probabilities, respectively, for each stage. Turnover of the terminal-stage cell is represented with a cell-death rate constant, *d*. (B) Representation of the cell lineage shown in (A), as a system of ordinary differential equations. In these equations, χ*_i_*(*t*) stands for the number (or concentration) of cells of type *i* at time *t*, with each equation expressing the rate of expansion (or contraction) of each cell type. For all cell types except the first and last, this rate is the sum of two terms: production by cells of the previous lineage stage, and net production (or loss) due to replication (or differentiation) of cells at the same lineage stage. For the first cell type, there is no production from a prior stage, and for the last cell type, loss due to cell death is included. (C) Steady state solution for the output (number of terminal-stage cells) of the general system of equations given in (B). Notice that this output is linearly, or more than linearly, sensitive to every system parameter, with the exception of the *v*_i_ for *i* > 0, which do not appear in the solution.

One way to identify the control needs of a system, and the strategies that may be used to address those needs, is to build models and explore their behavior. Figure 2A is a general representation of an unbranched cell lineage that begins with a pool of stem cells, ends with a postmitotic cell type, and possesses any number of transit-amplifying progenitor stages. If cells at each stage are numerous, and divisions asynchronous, then the behavior of such a system over time can be represented by a system of ordinary differential equations (Figure 2B) with two main classes of parameters. The *v*-parameters quantify how rapidly cells divide at each lineage stage (in particular, *v* = *ln2/*λ, where λ = the duration of a cell cycle). The *p*-parameters quantify the fraction of the progeny of any lineage stage that remains at the same stage (i.e., 1-*p* is the fraction that differentiates into cells of the next stage). Thus *p* may be thought of as an amplification, or replication, probability. As each lineage stage has its own *v* and *p*, we use subscripts to distinguish them.

Let us refer to the number of terminal-stage cells at any point in time as the *output* of a lineage system. From Figure 2B, we can see that a system is not stable—over time the output increases without bound—if *p*_i_ > 0.5 for any *i*. In contrast, if *p*_i_ < 0.5 for all *i*, stem and progenitor cells eventually run out, and the production of new terminal-stage cells stops. Provided terminal-stage cells do not die at an appreciable rate, such a system will reach a final state with a fixed number of terminal-stage cells. Finally, if *p*_0_ = 0.5, and *p*_i_ < 0.5 for *i* > 0, then the system will eventually produce terminal-stage cells at a constant rate. If such cells die or are shed with a constant probability per unit time (represented in Figure 2B by the rate constant *d*), then the output will approach a steady state, the solution for which is given in Figure 2C (solutions for certain cases of final-state behavior are also given in Protocols S1–S3, sections 5 and 6).

The result in Figure 2C describes a steady state that is quite sensitive to the system's parameters. For example, output is proportional to the number of stem cells (*χ*_0_, which remains constant at its initial value) and the rate of stem cell division (*v*_0_), and inversely proportional to the rate of terminal-stage cell death (*d*). Output varies even more sensitively with the *p*_i_. For example, increasing the value of a *p*_i_ from 0.45 to 0.4725—a 5% change—necessarily produces a 74% increase in the output of terminally differentiated cells. In engineering, parameter sensitivity is usually quantified as the fold change in output for a given fold change in the parameter (equivalent to the slope of a log-log plot of output vs. parameter). Thus, a linear relationship corresponds to a sensitivity of 1 (directly proportional) or −1 (inversely proportional). From Figure 2C, we may calculate that the sensitivity of the output to any *p*_i_ is *p*_i_/(1 − 3*p*_i_ + 2*p*_i_^2^), which for *p*_i_ < 0.5 is always greater than 1, and grows without bound as *p*_i_ approaches 0.5.

In well-regulated biological systems, parameter sensitivities ≥ 1 tend to be undesirable, since genetic or environmental variability can easily cause several-fold changes in the biological processes (levels of proteins, cell growth rates, etc.) that underlie parameters [27–29]. A system that cannot compensate for such variation is justifiably considered *fragile* (the opposite of robust).

Arguably, the most severe fragility of the system in Figure 2 is the constraint placed on the stem cell replication probability: *p*_0_ must be *exactly* 0.5 for a non-zero steady state to exist (effectively, the system's sensitivity to *p*_0_ is infinite). This is simply another way of stating that, unless exactly half of all stem cell progeny are stem cells, lineages eventually either go extinct or explode. Meeting this constraint can be achieved by having every stem cell undergo perfect asymmetric divisions, but that does not seem to be what normally happens. Rather, individual stem cells behave stochastically, sometimes giving rise to two, one, or zero stem cells (e.g., [6,8,30]). For the exact condition *p*_0_ = 0.5 to arise as a population average, when such behavior is not a cell autonomous imperative, is an extraordinary—and yet poorly understood—feature of stem cell systems.

### Feedback Control of Transit-Amplifying Cells: Insights from the Olfactory Epithelium

The idea that negative feedback is used to regulate tissue size and enhance regeneration is an old one. Over 40 y ago, Bullough [31] introduced the term *chalone* to refer to secreted factors that inhibit growth of the tissues and organs that secrete them. When a tissue is injured or partially removed, reduction in chalone levels would thus result in an up-regulation of tissue production. The view that chalones are secreted factors was supported by in vitro experiments, and by experiments with parabiotically joined pairs of animals in which partial hepatectomy in one animal led to liver cell proliferation in the other [32].

Although many of the original, in vitro–defined chalones have yet to be fully characterized, genetic studies in the 1990s demonstrated that growth and differentiation factor 8 (GDF8)/myostatin (*Mstn1*, MGI:95691), a member of the transforming growth factor β (TGFβ) superfamily of secreted signaling molecules, is specifically expressed by striated muscle cells (the terminal-stage cells of muscle lineages), inhibits the production of muscle, and when genetically eliminated from animals, results in the production of supernumerary muscle cells and an increase in muscle mass [33]. Subsequently, GDF11 (MGI:1338027)—a close relative of GDF8—was shown to be produced specifically by cells of the neuronal lineage of the mouse OE, and to provide feedback to inhibit the production of neurons (olfactory receptor neurons; ORNs) in that system [34]. Animals deficient in GDF11 also develop supernumerary ORNs. In recent years, factors that exert negative feedback on growth have been described for many other tissues, including skin, liver, bone, brain, blood cells, retina, and hair (Table S1). Many of these factors turn out to be members of the TGFβ superfamily, especially the TGFβ/activin branch of that superfamily [35].

**Figure 3:**
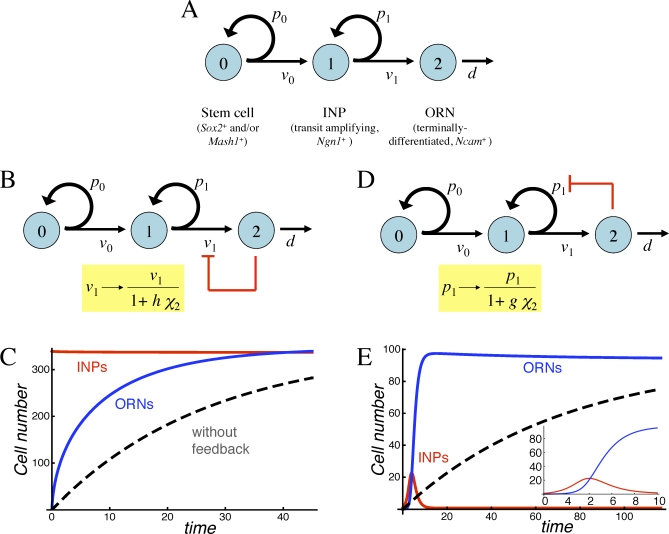
Strategies for Feedback Regulation of Transit-Amplifying Cells (A) The neuronal lineage of the OE, in which terminally differentiated ORNs are produced by committed transit-amplifying cells (INPs). (B) Negative feedback regulation of the INP cell cycle length (shown diagrammatically in red) can be modeled by making *v*_1_ a function of ORN numbers (*χ*_2_). (C) Simulated return to steady state of the system in (B) after removal of all ORNs. The parameters chosen provide the greatest improvement in regeneration speed (over what would occur in the absence of feedback; dashed line), consistent with progenitor cells comprising no more than 50% of the tissue mass (note that INP numbers [red curve] are virtually the same as those of ORNs [blue curve] at steady state). Cell numbers are expressed relative to the starting number of stem cells. (D) Negative feedback regulation of the INP replication probability (shown diagrammatically in red) can be modeled by making *p*_1_ a function of ORN levels (*χ*_2_). (E) Simulated return to steady state of the system in (D) after removal of ORNs. An inset shows the response at early times in greater detail. Note that progenitor load is now quite low, and regeneration is characterized by a burst of INP proliferation (red curve), followed by a wave of ORN production (blue curve). In (C and E), time is expressed in units of *ln*2/*v*_1_. Parameter values for (C) are *p*_1_ = 0.495, *d*/*v*_1_ = 0.0372, *v*_0_/*v*_1_ = 0.128, and h = 0.0734, and for (E) are *p*_1_ = 0.942, *d*/*v*_1_ = 0.0138, *v*_0_/*v*_1_ = 0.506, and *g* = 0.0449.

The OE of the mouse is a particularly useful system for studying lineage progression and feedback: It is continually self-renewing; its lineage stages are well defined; its cells can be studied in tissue culture; and it can be manipulated in vivo through genetic, chemical, or surgical means [36–38]. The OE neuronal lineage consists of a stem cell (which expresses *Sox2* [MGI: 98364], a gene encoding an SRY-box transcription factor), that gives rise to cells that express the proneural gene *Mash1* (*Ascl1*, MGI: 96919), which in turn give rise to cells that express another proneural gene, *Neurogenin1* (*Ngn1*; *Neurog1*; MGI: 107754), which in turn give rise to cells that exit the cell cycle and differentiate into ORNs. Recent data have raised the possibility that the *Sox2^+^* and *Mash1^+^* stages are not truly distinct, but rather are interchangeable states of the stem cell (K. K. Gokoffski et al., unpublished data). However, the *Ngn1* ^+^ cell—which is usually referred to as the Immediate Neuronal Precursor, or INP—is clearly a distinct transit-amplifying cell stage (Figure 3A; [34,39,40]).

The INP appears to give rise solely to ORNs, i.e., it does not represent a lineage branch point [39]. It is therefore interesting that the feedback actions of GDF11 seem to be directed specifically at INPs [34]: In vitro, GDF11 completely, but reversibly, arrests INP divisions, yet it has no effect on proliferation of *Mash1* ^+^ or *Sox2* ^+^ cells. In vivo, the increase in neuronal number observed in *Gdf11* ^−/−^ mice is accompanied by an increase in INPs, but not in *Mash1* ^+^ or *Sox2* ^+^ cells. These data imply that GDF11 regulates tissue size by inhibiting the proliferation of a committed transit-amplifying cell.

Because GDF11 can slow and even arrest INP divisions, it is natural to model GDF11-mediated negative feedback as an increase in the cell-cycle length of the INP (Figure 3B). Indeed, there is abundant literature showing that GDF11, GDF8, and other TGFβ superfamily members slow rates of progression through the cell cycle, at least in part by inducing cyclin-dependent kinase inhibitors [34,41–44]. Increasing the INP cell-cycle length is equivalent to decreasing its *v*-parameter, *v*_1_ (Figure 3B). Unfortunately, the result in Figure 2C states that the steady state outputs of lineage systems are independent of all *v* except for that of the stem cell (*v*_0_). This makes intuitive sense: if one decreases the division rate of an intermediate-stage cell in a lineage, the unchanged influx of cells from the previous lineage stage will cause its numbers to rise proportionately. From the standpoint of the lineage output, the two effects will cancel.

Apparently then, having GDF11 (or any other factor) feed back onto the INP cell division rate can be of no use in controlling the steady state level of ORNs. Could such feedback serve a function related to some other performance objective, such as rate control? As mentioned earlier, without control, lineage systems would be expected to return to steady state after a perturbation (i.e., regenerate) with a time scale similar to that over which terminal-stage cells normally turn over. In principle, feedback onto the cell division rate of a lineage intermediate could improve this. However, as explained below, the utility of this strategy turns out to be very limited:

Figure 3C shows a simulated regeneration experiment in which output, via GDF11, feeds back onto *v*_1_. At the start of the experiment, all ORNs are synchronously destroyed, and the time course of the return to steady state is followed (this type of perturbation can be produced experimentally by transecting the olfactory nerve or removing one or both olfactory bulbs of the brain [45]). For comparison, the figure also shows what the time course of the return to steady state would be in the absence of feedback (dashed line). From Figure 3C, we can see that feedback enables the system to regenerate faster, but we also observe a very high proportion of INPs (they are virtually as numerous, at steady state, as ORNs). It turns out that speeding up regeneration requires a large feedback gain (the parameter h in Figure 3B), which in turn drives down steady state ORN numbers (relative to other cells). If we define *progenitor load* as the percentage of the entire tissue that is composed of progenitors (stem cells plus INPs), we find that requiring the steady state progenitor load to be less than 50% limits any improvement in regeneration speed to about 3.2-fold; restricting progenitor load to 10% drops this value to about 2.6-fold (Figures S16 and S17 in Protocols S1–S3). In fact, experimental data indicate that the progenitor load in the OE is below 10% [46–48].

There is another cost of achieving fast regeneration through feedback on *v*_1_: the lower the progenitor load, the more necessary it becomes to use values of *p*_1_ that are perilously close to 0.5 (i.e., nearly half the output of INPs needs to be more INPs; Figures S16 and S17 in Protocols S1–S3). As discussed earlier, when *p*-parameters are close to 0.5, system output becomes extremely sensitive to small variations in those parameters (and thus very fragile).

All told, feeding back onto the rate at which INPs divide does not seem to be a particularly good control strategy. We wondered whether GDF11 might do a better job if it fed back onto a different parameter of INP growth: *p*_1_, the replication, or amplification, probability. Analysis of a model of this sort of feedback (Figure 3D) reveals several remarkable things:

First, with feedback on *p*_1_, the constraint *p*_1_ ≤ 0.5 goes away: Any INP replication probability allows for establishment of a steady state. Second, the fragility of the steady state output can be substantially reduced. In particular, sensitivity to the number of stem cells, the rate of stem cell division, and the death rate of terminally differentiated cells can be made arbitrarily small for appropriate parameter choices. Sensitivity to *p*_1_ can also be greatly reduced (to values <1), even if *p*_1_ is large (Figures S1 and S2 in Protocols S1–S3).

Finally, such a system can mount explosive regeneration after a perturbation. In some cases, the return to steady state can be as much as 100 times faster than in the absence of feedback. Furthermore, this can be accomplished without the need for a high progenitor load. Figure 3E shows this behavior for a particularly effective set of parameters. Notice how, in response to an acute loss of terminal-stage cells (ORNs), transit-amplifying cells (INPs) undergo a rapid, but transient, increase in number, following which, terminal-stage cells are restored rapidly to values close to steady state. This sort of behavior closely parallels what is seen in the OE following olfactory bulbectomy (in which ORN degeneration is induced by olfactory bulb removal): a transient upsurge in progenitor cell numbers, followed by a wave of neuronal production [20,40,46,49–51].

### GDF11 Controls Replication Probabilities

The fact that feedback aimed at *p*_1_ can, in theory, produce more useful and realistic behaviors than feedback aimed at *v*_1_, raised the possibility that the actual target of GDF11 might be *p*_1_, and not *v*_1_, as initially thought. To resolve this issue, we carried out tissue culture experiments in which mouse OE progenitor cells were pulse-labeled with 5-bromo-2-deoxyuridine (BrdU; to label cells undergoing division), and evaluated at successive times thereafter to determine when the progeny of dividing cells acquire immunoreactivity for NCAM, a marker for terminally differentiated ORNs. As shown previously, most dividing cells in these cultures are INPs, and their cell cycle length is about 17 h [39]. If all INP divisions result in production of ORNs, the acquisition of NCAM immunoreactivity by all BrdU-labeled cells should occur after sufficient time to progress through the rest of S-phase, G2-phase, M-phase, and however long it takes for NCAM levels to rise above the threshold of detection. If some INPs replicate, however, then a fraction of labeled cells will not express NCAM until one cell cycle (∼17 h) later (if the replicating fraction is high enough, some progeny will go through several cell cycles before acquiring NCAM immunoreactivity; cf. [39]). Accordingly, delay in the onset of NCAM expression can be used as a measure of the INP replication probability.

**Figure 4:**
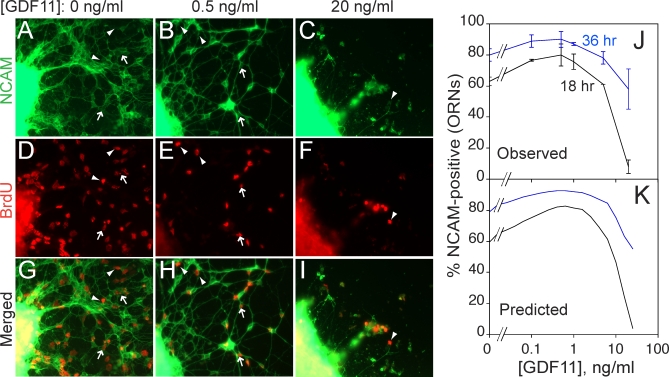
Experimental Demonstration That GDF11 Regulates *p*_1_ and *v*_1_ OE explants were cultured in various doses of GDF11. At 12 h, BrdU was added for 2 h and then washed out. Explants were fixed at various times after BrdU addition and immunostained for BrdU and NCAM expression. (A–I) Cultures grown in GDF11 concentrations of 0 (A, D, and G), 0.5 (B, E, and H), and 10 (C, F, and I) ng/ml, fixed 18 h after BrdU addition (previous studies have shown that 18 h is sufficient time for INP progeny that become ORNs to express NCAM [39]). NCAM immunofluorescence (green) is shown in (A–C); BrdU immunofluorescence (red) in (D–F); merged images in (G–I). Arrowheads point to examples of BrdU^+^/NCAM^−^ cells; arrows point to examples of BrdU^+^/NCAM^+^ cells. (J) Percentage of BrdU^+^ cells migrating out of OE explants that had differentiated (acquired NCAM immunoreactivity) by 18 h (black line) or 36 h (blue line), as a function of GDF11 dose. Low doses of GDF11 increase the proportion of INP progeny that differentiate (i.e., *p*_1_ decreases). At high dose, the effect reverses, with the NCAM^+^ fraction falling to near zero at 18 h, but recovering at 36 h. These data are consistent with a slowing of the cell cycle (*v*_1_) such that 18 h is not long enough to produce NCAM^+^ offspring (but 36 h is). This interpretation is consistent with a previous demonstration that high doses of GDF11 reversibly arrest the INP cell cycle [34]. (K) Simulation of the experiment in (J) by a model in which GDF11 affects both *p*_1_ and *v*_1_. Parameters used in the model are consistent with measured proportions of ORNs, INPs, and *Mash1* ^+^/*Sox2* ^+^ cells, as well as experimental data on the effects of GDF11 on BrdU pulse-labeling by INPs [34,39,40].

Figure 4 shows the effect of GDF11 (added to the culture medium 12 h prior to BrdU labeling) on acquisition of NCAM expression by BrdU pulse-labeled cells. In Figure 4J, data for two different “chase” periods are graphed. In the absence of GDF11, about 60% of BrdU-labeled cells become NCAM-positive within 18 h. In the presence of low levels of GDF11, this percentage rises as high as 75%, then falls again at high concentrations of GDF11 to less than 10%.

The increase in neuronal differentiation in response to low levels of GDF11 documents that GDF11 indeed suppresses INP replication (i.e., it lowers *p*_1_). The fact that this increase gives way to a large decrease in neuronal differentiation at high GDF11 levels is most likely due to the additional effect of GDF11 on the rate of cell cycle progression: As the INP cell cycle is progressively lengthened, one would expect that an 18-h chase period would cease being long enough to allow BrdU-labeled cells to go on to differentiate. This would lead to a sharp drop-off in the percentage of BrdU-labeled cells that acquire NCAM expression, but with longer chase times (e.g., 36 h), this effect would be overcome. That is indeed what is observed (Figure 4J). A numerical simulation of the experiment, in which GDF11 negatively regulates both *p*_1_ and *v*_1_, replicates both qualitative and quantitative features of the experimental data (Figure 4K; Protocols S1–S3, section 10).

### Performance Tradeoffs

Having the output of the OE lineage feed back onto *p*_1_ seems to be an effective strategy for meeting two control objectives: steady state robustness (low sensitivity to stem cell number *χ*_0_, cell division rates *v*_0_, and *v*_1_, and the death rate constant of the terminal-stage cell *d*) and rapid regeneration. But the ability to meet each objective separately does not guarantee that both can be met together (i.e., for the same sets of parameters).

**Figure 5:**
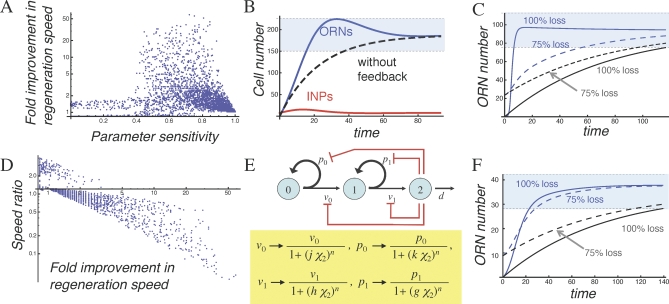
Performance Tradeoffs Associated with Feedback Strategies (A) Simulations of the model in Figure 3D were carried out for 20,000 randomly chosen sets of parameters (Protocols S1–S3, section 8). To simulate regeneration following a loss of terminal-stage cells, numbers of ORNs were set to zero, whereas numbers of stem cells and transit-amplifying cells (INPs) were set to their steady state values. For each parameter set, the time it took for ORN numbers to return to and remain within 20% of their steady state values was taken as an objective measure of regeneration time, and cases with very long regeneration times (>29 transit-amplifying cell cycle lengths) are not shown (see Protocols S1–S3). Next, the time that would have been required to generate the same number of ORNs, from the same initial conditions but in the absence of feedback, was calculated. Finally, the ratio of the two regeneration times (with and without feedback) was considered to be the fold improvement in regeneration speed due to feedback. For each parameter set, this was plotted against the sensitivity of the steady state solution to variation in either the initial number of stem cells, the stem cell cycle time, or the normal lifetime of ORNs (all three sensitivities are equal). The data show that only those parameter sets that do not support a robust ORN steady state (abscissa values >0.4) show substantial improvement in regeneration speed (ordinate values >2). (B) Simulated regeneration for the set of parameters in (A) that showed the greatest improvement in regeneration consistent with sensitivity to parameters remaining below 0.4 (this corresponds to a 32% change in steady state values for a 2-fold change in parameters). As in Figure 3, the blue curve denotes ORN numbers, the red curve shows INPs, and the dashed line shows the time course over which regeneration would proceed in the absence of feedback. The light-blue zone denotes the range of cell numbers within 20% of the steady state value for ORNs. (C) Simulated regeneration for the parameters used in Figure 3C, but starting from two different initial conditions. The solid blue curve shows the dynamics of ORN recovery after complete removal of existing ORNs; the solid gray curve illustrates the predicted rate of recovery in the absence of feedback. The dashed blue and gray curves present corresponding simulations where ORN numbers were initially depleted only 75%, rather than completely. Under these conditions, nearly all improvement in regeneration is lost. (D) To quantify the effect of initial conditions on regeneration speed, a ratio was defined (“speed ratio”) that indicates how much faster (or slower) regeneration from 75% ORN depletion is than regeneration from 100% depletion. In the absence of feedback, this ratio should have a value of approximately 1.22 (regeneration from partial depletion should take slightly less time than regeneration from total depletion). This ratio was calculated for each of the random cases shown in (A), and the results were plotted against the fold improvement in regeneration speed (from [A]). The abscissa is drawn at an ordinate value of 1.22. The plot shows that the more one gains in regeneration speed from 100% depletion, the more one sacrifices in regeneration speed from 75% depletion. (E) Negative feedback effects of activin and GDF11 (shown diagrammatically in red) can be modeled by multiplying the replication probabilities and cell division rates of stem cells and INPs, respectively, by decreasing functions of ORN numbers (χ_2_). In this case, Hill functions are used, with parameters *g*, *h*, *j*, and *k* representing the feedback gains, and *n* the Hill coefficient. (F) Example of a case with both activin and GDF11 feedback. Notice that now, regeneration from initial conditions of 75% ORN depletion is nearly as fast as regeneration from 100% ORN depletion (compare with [C]). Parameters for this case are: *p*_0_ = 0.507, *p*_1_ = 0.546, *d*/*v*_1_ = 0.0116, *v*_0_/*v*_1_= 0.965, *g* = 1.258, *h* = 1.03, *j* = 0.0394, and *k* = 1.683 (and the ordinate axis has been scaled for easier comparison with [C]). In (B), (C), and (F), time is expressed in units of *ln*2/*v*_1_.

As it turns out, the two strategies are largely incompatible. Numerical exploration of the parameter space shows a strong negative correlation between robustness and enhancement of regeneration (Figure 5A). Cases for which the sensitivity to *χ*_0_, *v*_0_, or *d* is less than 0.4 (i.e., a 2-fold change in parameter will cause ≤32% change in output), generally do not exhibit acceleration in regeneration speed exceeding approximately 8-fold. In fact, this result is skewed by cases in which regeneration speed goes from extremely slow (in the absence of feedback) to merely very slow. If one restricts the analysis to cases in which regeneration from complete loss of terminal-stage cells is 80% complete in fewer than 29 transit-amplifying cell cycles (∼20 d for INPs), then to achieve parameter sensitivities less than 0.4, the best possible improvement in regeneration speed is less than 2-fold (Figure 5A and 5B).

Upon closer inspection, other unfortunate tradeoffs can be seen: For the cases in Figure 5A, improvement in regeneration speed was calculated by simulating a complete loss of terminal-stage cells and then measuring the return to steady state. If we use a milder perturbation (a 75% loss of terminal-stage cells), but otherwise the same parameters, the return to steady state is, unexpectedly, quite slow (Figure 5C). The need to sustain injury that is massive before regeneration can be rapid hardly seems like a good strategy for an organism in the real world. To define the conditions under which this phenomenon occurs, we calculated, for all the cases in Figure 5A, the ratio of two regeneration times: the time for regeneration from a 100% perturbation, and the time for regeneration from a 75% perturbation. In Figure 5D, this value (“speed ratio”) is plotted against fold improvement in regeneration speed (for the 100% perturbation, compared with no feedback). The data show that the speed of regeneration following massive injury cannot be improved by more than about 3-fold, without sacrificing the speed of regeneration following less-than-massive injury.

Altogether, tradeoffs among regeneration speed, sensitivity to parameters, and sensitivity to initial conditions make the control strategy of having GDF11 feed back onto *p*_1_ less attractive than it originally seemed. Analysis of cases in which GDF11 inhibits both *p*_1_ and *v*_1_ (which corresponds most closely to what GDF11 does in vitro; Figure 4J and 4K) shows some improvement in the tradeoff between regeneration speed and parameter sensitivity, but the effect is not dramatic (Figure S18 in Protocols S1–S3). Accordingly, we wondered whether additional control elements might still be missing.

### Two Loops Are Better Than One

As mentioned in Table S1, many feedback inhibitors of tissue and organ growth belong to the TGFβ superfamily of growth factors, with those of the TGFβ/activin branch (which signals through the intracellular proteins Smad2 and Smad3) being the most highly represented. Recently, we found that activinβB (*Inhbb*; MGI: 96571; hereafter referred to simply as “activin”) is highly expressed in the OE and, like GDF11, has growth-inhibitory effects on the neuronal lineage. Unlike GDF11, however, activin's effects are aimed specifically at the *Sox2* ^+^ and *Mash1* ^+^ populations, and not at INPs (K. K. Gokoffski et al., unpublished data). This implies that two feedback loops exist in the OE, one aimed at stem cells, and one aimed at transit-amplifying cells (Figure 5E).

Like GDF11, activin could potentially feed back onto a *v*-parameter (namely *v*_0_, the rate of stem cell division) or a *p*-parameter (namely *p*_0_, the stem cell replication probability), or both. For technical reasons, a pulse-chase experiment similar to that in Figure 4 cannot be performed to sort this out. However, we infer that feedback onto *p*_0_ must occur, because *Sox2* ^+^ and *Mash1* ^+^ populations are markedly expanded in the OE of *ActβB* ^−/−^ mice (K. K. Gokoffski et al., unpublished data). If activin only regulated *v*_0_, loss of activin would result in stem cells that cycle faster, but it could not increase their numbers.

Interestingly, when we add the feedback effects of both activin and GDF11 into the equations for the behavior of the ORN lineage, the expression for the steady state value of ORNs becomes very simple: (2*p*_0_ − 1)/*j*, where *j* is the feedback gain for activin (Protocols S1–S3, section 4). This constitutes a dramatic improvement in robustness—the system will, at steady state, always produce the same number of terminal-stage cells regardless of how many stem cells it starts with, how fast stem cells divide, or how quickly terminal-stage cells are lost.

Perhaps even more strikingly, the problematic constraint that the stem cell population must intrinsically “know” to replicate exactly half the time (*p*_0_ = 0.5) vanishes. As long as *p*_0_ > 0.5, feedback automatically ensures that the stem cell population behaves in the necessary way.

All of these improvements in steady state control come solely from the single feedback loop of system output onto *p*_0_. When such a loop is in place, however, feedback onto other *p*- and *v*-parameters can have additional useful effects:

Consider, for example, the matter of regeneration speed, which we previously found could be increased through feedback onto *p*_1_ or *v*_1_, but only by sacrificing robustness, low progenitor loads, or the ability to regenerate quickly from a variety of initial conditions (Figures 3C and 5A–5D). When feedback is directed solely at stem cells, we also fail to achieve good performance: Feedback onto *p*_0_ hardly improves regeneration speed at all (Figure S19 in Protocols S1–S3), and although feedback onto *p*_0_ and *v*_0_ together can produce fast rates of regeneration (Figure S21 in Protocols S1–S3), those rates still show a very sensitive dependence on initial conditions (Figure S22 in Protocols S1–S3).

In contrast, when feedback is directed at both stem and transit-amplifying cell stages—i.e., the arrangement that actually occurs in the OE—it becomes possible to achieve very rapid regeneration, with low progenitor loads, from almost any starting conditions. This includes conditions in which variable numbers of stem, transit-amplifying, or terminal-stage cells are depleted. Figure 5F shows an example of such a case.

**Figure 6:**
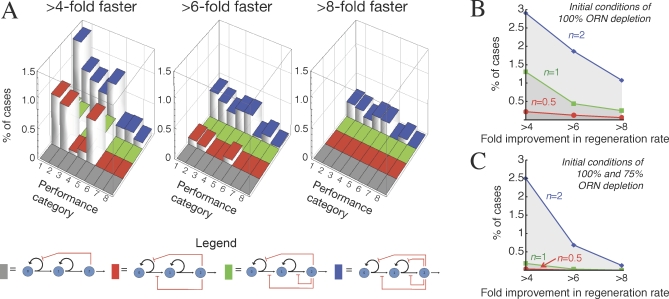
Effects of Feedback Configuration on Regeneration from Diverse Perturbations (A) Four different feedback architectures (shown diagrammatically beneath the word “Legend”) were modeled and investigated for their ability to support rapid regeneration from multiple starting conditions. For each model, 20,000 random parameter sets were explored (see Protocols S1–S3, section 8) using simulations that started from initial conditions corresponding to four different perturbations of the steady state. For all 640,000 solutions, the fold improvement in ORN regeneration speed was calculated as in Figure 5. The bar graphs depict the fractions of random parameter sets for each model that produced at least a given amount of improvement in regeneration speed for one or more sets of initial conditions. The four different feedback architectures are designated by different colored bars (see diagrams under “Legend”): feedback on *p*_0_ (grey); *p*_0_ and *v*_0_ (red); *p*_0_, *v*_0_, and *v*_1_ (green); and *p*_0_, *v*_0_, *p*_1_, and *v*_1_ (blue). The heights of bars give the fraction of parameter sets that produced at least the indicated amount—4-fold (left graph), 6-fold (middle graph), or 8-fold (right graph)—of improvement in regeneration speed. The “Performance categories” refer to different combinations of initial conditions: Cases included in performance category 1 are those that met the desired level of improvement in the speed of regeneration following a complete loss of terminal-stage cells. In category 2, the perturbation was a complete loss of both terminal-stage and transit-amplifying cells. In category 3, it was a 75% loss of terminal-stage cells. In category 4, the perturbation was a complete loss of terminal-stage and transit-amplifying cells and a 50% loss of stem cells. Category 5 cases are those parameter sets that met the criteria for initial conditions of both categories 1 and 2. Category 6 refers to those that did so for both categories 1 and 3. Category 7 refers to those that did so for both categories 1 and 4. Category 8 refers to cases in which the parameter sets met the indicated criterion for all four initial conditions. The data show that rapid regeneration from a variety of initial conditions is facilitated by feedback on the *p*-parameters of at least two progenitor cell stages. (B and C). For the system with feedback on *p*_0_, *v*_0_, *p*_1_, and *v*_1_ (i.e., the system depicted with blue bars in [A]), the graphs show the percentages of random parameter sets that meet the regeneration-rate criteria on the abscissa (4-fold, 6-fold, or 8-fold improvement in regeneration speed), as a function of the Hill coefficient, *n*, used in the expressions for the feedback functions. The results in (B) were obtained by only considering simulations that started from a 100% loss of terminal-stage cells. Cases presented in (C) are those that also met the same regeneration-rate criteria for simulations starting from a 75% loss of terminal-stage cells. The results show that larger *n* substantially increases the fraction of cases with rapid regeneration. This effect is especially prominent when the performance criteria call for fast regeneration from more than one set of initial conditions (C).

Not only is such performance possible, it occurs over a substantial fraction of the parameter space (that is, a substantial fraction of randomly chosen sets of parameters meet all of these performance objectives). Figure 6A shows graphically how, as feedback loops are added one at a time, good control (robustness, stability, low progenitor load, and fast regeneration from a variety of conditions) is found over an increasing fraction of the parameter space (exploring wide ranges on all parameters). In evaluating the magnitude of this effect, it should be noted that fractions of parameter space in the range of 0.1%–1.5% are remarkably high, given the numbers of parameters in each model (cf. [52]). For example, when there are eight independent parameters (as there are when feedback is directed at *p*_0_, *v*_0_, *p*_1_, and *v*_1_), good performance over 0.1% of the parameter space means that the average parameter value “works” over 42% (∼0.001^1/8^) of its range. In Figure 6, most parameters were explored over three orders of magnitude (i.e., they were randomly selected from a log-uniform distribution with a 1,000-fold range), so for such cases, 42% means that the average parameter can be varied over an 18-fold range (1,000^0.42^) without loss of good control.

What is the significance of a control system that works over a large portion of its parameter space? It means that the output of the system can be adjusted (through changes to the parameters) without the control strategy itself being jeopardized. From a biological perspective, this means that the system is evolvable, a feature we should expect to observe in most biological control systems [53].

### Sensitivity and Geometry

So far, we have said much about the cell stages and processes that are targets for feedback in cell lineages, and little about the quantitative details of feedback signals. In Figures 3 and 5, feedback was modeled using Hill functions; these are natural choices for the actions of secreted growth factors, since saturable binding of ligands to receptors is usually well described by them [54].

Hill functions typically employ a parameter *n*, the Hill coefficient, to fit dose-response relationships that are positively (*n* > 1) or negatively (*n* < 1) cooperative. In Figures 3, 5, and 6A, a Hill coefficient of 1 was used, but more detailed exploration of the two-loop feedback system (with feedback on *p*_0_, *v*_0_, *p*_1_, and *v*_1_) shows that system performance increases steadily as *n* goes from 0.5 to 2 (Figure 6B and 6C). This makes intuitive sense if we consider that high values of *n* make Hill functions more switch-like. In the limit of a perfect switch (infinite *n*), the drive for increased growth would be zero when output is at the desired value, yet maximal when output is even slightly below the desired value. Such a strategy clearly achieves the fastest possible regeneration following a perturbation.

In biology, dose-response relationships that are fit by Hill coefficients other than 1 arise for a variety of reasons besides biochemical cooperativity; these include buffering, competition, feedback, and distributed multistep reactions [55–57]. Generally speaking, Hill coefficients quantify the sensitivity of output to input (in the limit of high input, the Hill coefficient and the engineering definition of sensitivity are equivalent). Thus, in our models of feedback in the OE, Hill coefficients near 1 mean that the amount of activin and GDF11 signaling in stem cells and INPs (respectively) is roughly proportional (over some range) to the number of cells producing activin and GDF11 (i.e., the size of the tissue).

It occurred to us that this situation—feedback proportional to tissue size—might not be so easy for tissues to achieve. As a tissue grows in size, one can certainly envision the total amount of material it produces increasing proportionally, but it is the concentrations—not the amounts—of factors like GDF11 and activin to which cells respond. How the concentrations of secreted ligands change as tissues grow turns out to depend both on issues of geometry (tissue shape and boundary properties), and issues of cell biology (rates of ligand capture and turnover).

For example, consider a hypothetical tissue surrounded by a boundary across which macromolecules cannot diffuse. In this case, a secreted protein produced everywhere in the tissue should reach a steady state concentration determined by the balance between production and local degradation. If the tissue doubles in size, it will make twice as much of the protein, but distribute it over twice the volume. The result will be no change in concentration. In a truly “closed” tissue, secreted molecules cannot be used as part of a strategy for growth control.

Fortunately, epithelia, such as the OE, are not closed systems. Although tight junctions between epithelial cells prevent escape of molecules from the apical surface, there appears to be little or no impediment to diffusion across a basal lamina into the underlying connective tissue stroma [58]. Within such a geometry, we may use approaches developed for the analysis of morphogen and signaling gradients [59–62] to calculate expected intraepithelial distributions of secreted molecules (Protocols S1–S3, section 11).

**Figure 7:**
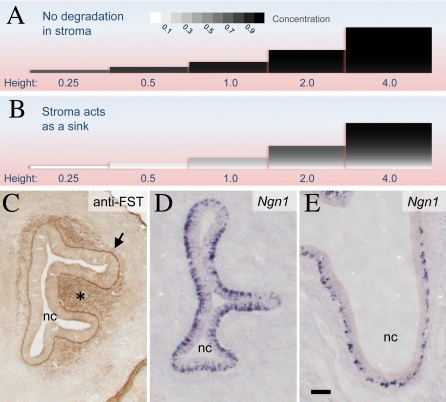
Effects of Geometry and Degradation on Levels of Secreted Molecules within Epithelia (A and B) Polypeptides secreted into the intercellular space of an epithelium are removed by two processes: diffusion into underlying connective tissue (stroma) and degradation within the epithelium. Given a molecule's rate of production, its diffusivity, its rate of uptake and degradation, and the geometry of the epithelium, one may calculate its concentration, at steady state, at every location within the epithelium. Here, such calculations are shown graphically, for epithelia of different thicknesses (in each picture, the epithelium is oriented with the apical surface at the top). Epithelial thickness (“height”) is scaled according to the decay length of the molecule of interest. The shading in each picture depicts the concentration of the secreted molecule, with black representing the limiting concentration that would be achieved in an epithelium of infinite thickness. In (A), the degradation capacity of the stroma is set at a relatively low value, one-tenth of that in the epithelium. In this case, intraepithelial concentrations of secreted molecules plateau while the epithelium is very thin. In (B), the degradation capacity of the stroma is ten times of that in the epithelium, so that few molecules that enter the stroma escape undegraded. Now, there is a large (and more physiological) range of epithelial thickness over which the concentrations of secreted molecules change appreciably with tissue size. This is particularly true near the basal surface of the epithelium (see also Figures S27 and S28 in Protocols S1–S3). (C) Follistatin (FST), a molecule that binds GDF11 and activin essentially irreversibly, is present at high levels in the basal lamina (arrow) and stroma (asterisk) beneath the embryonic day 13 OE. Association of FST with basal laminae is consistent with its affinity for extracellular matrix components [102]. Scale bar represents 100 μm. (D and E) INPs (visualized with *Ngn1* in situ hybridization) become progressively localized to the basal surface of the OE over the course of development. (D) = embryonic day 12.5; (E) = embryonic day 18.5. nc = nasal cavity. Scale bar in (E) represents 100 μm.

The results of these calculations (Figure 7) show that when an epithelium is very thin, concentrations of secreted molecules in the intercellular space initially go up linearly with tissue size, but soon level off. Does the normal size range of the OE (adult thickness ∼80 μm) lie in the linear region, or on the plateau? The answer depends on two factors: The first is the *decay length* of the molecule of interest. This is the average distance a molecule travels in tissue before being captured and degraded by cells, and is a function of its diffusion coefficient and rate of receptor-binding and degradation.

The second factor is the ratio of decay length within the epithelium to decay length in the adjacent stroma (which, in most cases, simply reflects how much faster or slower degradation proceeds in one location versus the other). If that ratio is low—i.e., if molecules that diffuse into the stroma are not quickly degraded—then intraepithelial concentrations will be poorly sensitive to tissue size long before the epithelium reaches even a single decay length in thickness (Figure 7A; Figure S27 in Protocols S1–S3).

In contrast, if the ratio of decay lengths between epithelium and stroma is high—i.e., if the stroma acts as a sink, quickly eliminating molecules that enter it—then average intraepithelial concentrations will rise more gradually, and not plateau until the epithelium has reached a size of several decay lengths (Figure 7B). This effect is more pronounced if the concentration that matters is the concentration close to the basal surface of the epithelium, and not the average concentration over the entire epithelial thickness. At this basal location, concentration varies linearly with tissue size for many decay lengths (Figure 7B; Figure S28 in Protocols S1–S3).

Estimates of intraepithelial decay lengths of TGFβ superfamily polypeptides, obtained both from measurements of morphogen gradients and from first-principles calculations, tend to be in the range of tens of micrometers [59,63–65], i.e., on the order of, or less than, the normal thickness of the OE. This suggests that it would be difficult to use activin and GDF11 as “reporters” of OE size, if these molecules merely leaked into the stroma and were not rapidly degraded there (as in Figure 7A): once the OE grew beyond 0.2 decay lengths in thickness, the poor sensitivity of activin and GDF11 concentrations to OE size would be functionally equivalent to feedback described by Hill coefficients less than 0.5. As already demonstrated (Figure 6B), such low Hill coefficients undermine good control.

Accordingly, we infer that it would be strategically advantageous for the OE to possess a mechanism that rapidly removes activin and GDF11 in the underlying stroma, as well as a mechanism for restricting the location at which cells measure the level of activin and GDF11, to the basal surface of the tissue. Remarkably, the OE seems to have both:

First, the OE contains large amounts of the protein follistatin (FST; MGI: 95586) in its basement membrane and stroma (Figure 7C; [34,66]). FST not only binds and inhibits both activins and GDF11, it does so irreversibly, effectively eliminating them [67–69]. That FST plays a central role in regulating GDF11 and activin function in the OE has recently been demonstrated genetically ([34] and K. K. Gokoffski et al., unpublished data); what the analysis here provides is an explanation for why FST is used by the OE, and why it should be found primarily beneath the epithelium.

Second, the progenitor cells of the OE that respond to activin and GDF11 become increasingly polarized, during early development, to the basal side of the epithelium; eventually they lie within a few cell diameters of the basement membrane. This is shown in Figure 7D and 7E, using in situ hybridization for *Ngn1* to visualize INPs. Thus, the only concentrations of GDF11 and activin that progenitor cells sense are likely to be those near the basal surface of the epithelium. Interestingly, in many other types of epithelia, stem/progenitor cells also localize near the basement membrane, an observation that has long suggested the existence of a specialized microenvironment, or “niche,” in this region [70].

### Final-State Systems

The OE, a self-renewing tissue, maintains its size by continuous replacement of dying cells [51,71]. Some organs—such as the mammalian brain—achieve a final size during development and largely cease proliferating [72–74]. Such final-state (as opposed to steady state) systems also may be modeled using the equations in Figure 2, by setting the terminal cell death rate constant, *d*, to zero, and allowing replication probabilities to be below 0.5. Like steady state systems, they can be quite fragile.

**Figure 8:**
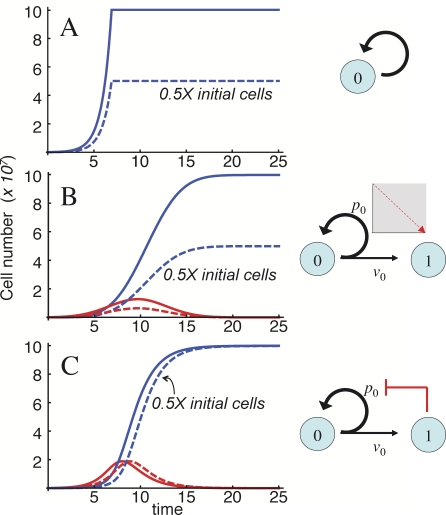
Behaviors of Final-State Systems Three different ways are shown by which an initial pool of 10^5^ progenitors (solid curves) or 5 × 10^4^ progenitors (dashed curves) can generate 10^8^ terminally differentiated cells. Differences among the three mechanisms are illustrated by the diagrams at right. (A) Simple exponential expansion. The progenitor pool expands for just enough time to produce the desired output and then stops. Halving the starting number of progenitors halves the output. (B) Nowakowski-Caviness system: progenitors undergo both replicative and differentiative divisions, according to a replication probability *p*_0_, which starts at *p*_max_ > 0.5 and declines linearly to *p*_min_ < 0.5 at time τ. As in (A), halving the initial progenitor cell number halves the output. The output is also highly sensitive to values of *p*_max_ and τ. (C) System with negative feedback on *p*_0_. Feedback is modeled as previously, using a Hill function (without cooperativity in this example). Halving the starting progenitor pool now produces almost no change in output (there is, however, a one cell cycle lag in reaching the final state). Sensitivity to *p*_0_ is also reduced. In each panel, time is expressed in units of *ln*2/*v*_1_. Parameter values were, in (A), time of cessation of cell division = 6.91/*v*_0_; in (B), *p*_max_ = 1, *p*_min_ = 0, and τ = 19.4; and in (C), *p*_0_ = 0.9, and γ = 3.14 × 10^8^ (where γ is the feedback gain).

This point is well illustrated by the mouse brain, which is composed of approximately 10^8^ cells of neural lineage (neurons and glia; [16]). Although brain cell number varies from mouse to mouse, within a given strain, the coefficient of variation is small, about 5% [16]. If we hypothesize that the brain is “founded” by a pool of 10^5^ progenitors (probably an overestimate), and we make the simplifying assumption that no cells die during development, then a 1,000-fold expansion in cell numbers is needed (Figure 8). One way to accomplish this would be to have all progenitors replicate for a time equal to ten cell-cycle lengths (2^10^ = 1,024), and then stop. With this strategy, final cell number will be linearly sensitive (i.e., proportional) to the initial size of the progenitor pool (Figure 8A), and much more than linearly sensitive to the average length of the cell cycle, or the length of time allowed for proliferation (a mere 5% change in either parameter would produce a 30% change in output). If the brain is founded by fewer progenitors, this fragility only becomes more severe.

Now, let us consider a slightly more sophisticated strategy: a progenitor pool that undergoes a mixture of replicative and differentiative divisions, with a replication probability *p* set below 0.5. Because proliferating cells replicate less than half the time, the progenitor pool runs out, and the tissue approaches a final state gradually, without need to count cell cycles or time. In this case, the final state is still linearly sensitive to the initial size of the progenitor pool, and although no longer sensitive to time or cell-cycle parameters, it is extremely sensitive to the value of *p* itself, which must be very close to 0.5 to produce a 1,000-fold expansion in cell numbers (Protocols S1–S3, section 5).

One way to circumvent this extreme fragility is to allow *p* to change over time, starting above 0.5 (promoting progenitor expansion), then falling below 0.5 (driving progenitor cell extinction). In fact, this very mechanism, illustrated in Figure 8B, was introduced by Nowakowski et al. [75] to explain the biphasic expansion and contraction of progenitor pools in the cerebral cortex, and it is supported by considerable experimental data (e.g., [76]). Mathematical analysis (Protocols S1–S3, section 5) shows that sensitivity to *p* is reduced by this strategy, but it still remains very high (Figure S5 in Protocols S1–S3). Moreover, the system now becomes quite sensitive to the rate at which *p* declines (relative to the cell-cycle length; Figure S4 in Protocols S1–S3). In addition, such a system is still linearly sensitive to the initial size of the progenitor pool (Figure 8B).

Given how difficult it seems to be to achieve even modestly robust final states, it is striking how much can be accomplished with the addition of just a single feedback loop. Figure 8C illustrates a case much like the one in Figure 8B, in which the *p*-value of a progenitor pool declines over time, but this time, the decline is caused by feedback from terminal-stage cells. Superficially (that is, when not perturbed), it behaves just like the Nowakowski-Caviness model [75], displaying expansion, contraction, and disappearance of a stem cell pool. Yet in this case, a 2-fold change in the initial number of stem cells produces only a minute (0.14%) change in the final state! Even sensitivity to the initial value of *p* can be much lower (<5) than in the case without feedback (Figures S6–S11 in Protocols S1–S3). Just as with our analysis of steady state systems, this sort of behavior arises only when feedback regulates replication probabilities (*p*-parameters), and not when it regulates cell cycle lengths (*v*-parameters).

## Discussion

At the start of this article, it was argued that, compared with other biological pathways, cell lineages should be especially fragile to intrinsic variability and external perturbation. Yet for many tissues and organs, size, growth rate, and cellular composition are actively maintained within narrow limits. The goal of the present study was to identify basic strategies that enable lineage pathways to achieve tight control of growth. The mammalian OE provided a platform for pursuing this investigation, which exploited both modeling and experimentation. Because some conclusions—those having to do with distributions of possible regeneration speeds—were supported by the computational exploration of parameter spaces, and not derived from models analytically, it is formally possible that additional system behaviors relevant to these conclusions were missed. However, given the large parameter ranges used, the smoothness of the feedback functions, and the regularity of the solutions (cf. [77]), this seems unlikely.

### The Power of *p*

Using this approach, we showed that a feedback configuration that exists in the OE—with regulation at two sequential lineage stages—achieves a variety of important control objectives, including limited parameter constraints, decreased parameter sensitivities, improved regeneration speed, minimized influences of initial conditions, and evolvability. The core of this strategy is feedback inhibition of replication probabilities, referred to here as *p*-parameters. Such feedback is highly useful, not only to tissues that continuously turn over (such as the OE), but also to tissues that are generated during a discrete period by a transient pool of progenitors (such as the mammalian brain). In contrast, feedback on rates of cell division was found to be of only marginal value unless also combined with feedback on *p*.

The data in Figure 4 provide experimental verification that GDF11 in fact acts by lowering the replication probability of neuronal transit-amplifying cells. Recent work suggests that GDF8/myostatin works similarly in muscle—lowering the probability that progenitors replicate and increasing the probability that they differentiate [78]. Thus, action on *p* may be a common feature of feedback inhibitors of tissue and organ growth. The molecular mechanisms by which such an action is achieved are currently unknown. Like many members of the TGFβ superfamily, GDF11 and GDF8 up-regulate the expression of cyclin-dependent kinase inhibitors (e.g., *p21* ^cip1/waf1^, *p27* ^kip1^), which are implicated in both inhibiting cell-cycle progression and promoting differentiation (e.g., [34,41,78–80]). Formally, it is possible that these two effects are linked, i.e., the probability that a cell replicates or differentiates is determined by how long its cell cycle lasts. Indeed, in the developing mammalian brain, an observed progressive decline in *p*-values is matched by a progressive increase in cell cycle lengths [73,76].

However, we do not favor the interpretation that cell cycle length dictates replication probability, for two reasons. First, as implied by Figure 4F and 4G (and unpublished data), the dose of GDF11 needed to maximally decrease *p*_1_ in the OE is considerably lower than that needed to prolong the cell cycle. Second, several growth factors are known to increase replication probabilities without altering cell cycle parameters. For example, the FGFs act in this way both on neural progenitors [39,81] (including the INPs of the OE) and on muscle progenitors [82]. The inhibitory effects of leukemia inhibitory factor on mouse embryonic stem cell differentiation also occur without changes to cell cycle parameters [83]. From this, we conclude that it is at least possible for *p*- and *v*-parameters to be regulated independently.

### Strategies of Control: Human versus Biological

In engineering, feedback control is often classified by the relationship between a measured “error”—usually the difference between actual and desired output values—and a control signal, i.e., a quantity that is fed back. “Proportional control” means the control signal is proportional to the error. In “integral control,” the signal is proportional to the integral, over time, of the error. “Derivative control” implies a control signal proportional to the derivative, with respect to time, of the error.

Each strategy has strengths and weaknesses, and engineers often combine them. Proportional control, for example, can never fully compensate for a steady perturbation, because only when output is not at the desired level does a non-zero control signal exist. Proportional control can decrease a system's response time, but at the expense of gain (the amount of amplification from input to output). In the lineage pathways described here, feedback onto *v*-parameters clearly exhibits the hallmarks of proportional control: Feedback onto *v*_0_ can reduce, but never eliminate, parameter sensitivities; and feedback onto *v*_1_ can speed regeneration, but only by decreasing the ratio of terminal-stage cells to progenitors.

Integral control, in contrast, will fully compensate for a steady perturbation, producing a steady state that is completely independent of many external and internal influences; this phenomenon is sometimes referred to as “perfect adaptation” [84]. Integral feedback also tends to speed the rate of approach to steady state, but often at the risk of overshoots, undershoots, and oscillations. In the lineage pathways described here, feedback onto *p*_0_ exhibits the hallmarks of integral control: output that is independent of many parameters, very rapid regeneration, and a tendency toward oscillation (the latter behavior is described in detail in [77]). To understand how feedback onto *p*_0_ implements integral control, it suffices to note that any steady deviation in the replication probability of stem cells above (or below) 0.5 leads to an ever-increasing (or ever-decreasing) effect on system output. In this way, output naturally follows the time integral of the difference between the effective value of *p*_0_ (i.e., *p*_0_ as modified by feedback) and the value 0.5. Feeding back output onto *p*_0_ thus represents true integral feedback control.

Derivative control is often used by engineers to suppress instabilities associated with integral control, but it suffers from its own problems, such as a tendency to amplify noise. At this point, it is unclear whether derivative control is used in lineage pathways. Intriguingly, it has been noticed in the OE that the expression of GDF11 is stronger in immature than mature cells [34], raising the possibility that GDF11 levels could track, at least to some degree, the rate of change (i.e., the time-derivative) of system output, and not just the current output.

### To Stem or Not to Stem?

In the biological literature, a sharp distinction between stem cells and transit-amplifying cells is classically drawn: the former are said to divide indefinitely and asymmetrically, regenerating themselves with each division, whereas the latter are said to have only limited capacity for self-replication [85,86]. The results of the present study lead us to question whether stem and transit-amplifying cells necessarily exist. By this we mean it is possible to have lineages in which all cells have the same intrinsic proliferative tendencies, yet typical stem and transit-amplifying *behaviors* are observed, solely as a consequence of feedback control. The only conditions required for this to happen are (1) cells should have an intrinsic tendency to self-replicate more than half the time (*p* > 0.5), and (2) the output of the lineage should negatively regulate replication probabilities (feedback on *p*).

For example, in a lineage with two sequential stages of dividing cells, if the output feeds back onto the *p*-parameters of both cell types, then either of two steady states is possible, depending on the relative strength of the two feedback loops (see Protocols S1–S3, section 4). In one of these, the first cell stage exhibits classic stem cell behavior, i.e., its population self-replicates exactly half the time, and the second cell exhibits classic transit-amplifying behavior, i.e., its population appears to undergo limited divisions. In the other, the first cell stage is extinguished, and the second cell exhibits stem cell behavior (see [77] and Protocols S1–S3, section 4, for further discussion; see also the related discussion in [87]). Which cell becomes the stem cell is thus determined by the feedback, and not anything intrinsic to that cell.

It is easy to see how other typical behaviors of stem cell systems can also be the consequences of control. For example, with sufficiently large negative feedback onto *v*-parameters, progenitor cell populations will appear “slowly cycling” or even “resting,” and would be observed to be “label-retaining” (see Protocols S1–S3, section 7, especially Figure S13). These arguments lend strong quantitative support to a view that has been gathering increasing support, namely that the definition of stem cell should be seen as one of context and condition, not of cell type [6,88,89]. The work presented here additionally suggests that much the same thing could be said about transit-amplifying cells. Interestingly, recent work on epidermis has shown that cells long thought to be classical transit-amplifying cells in fact do not display the seemingly essential property of limited self-replication [30,90]; instead they behave in a probabilistic manner that is fully consistent with the models presented here.

### No Free Lunch

In engineering, it is widely accepted that one cannot make a device robust in every possible way to every possible perturbation. Usually, strategies that eliminate one fragility come at the expense of creating new ones, a phenomenon underlying the characteristic “robust-yet-fragile” architecture of highly engineered systems [91,92]. Evidence for such tradeoffs can be seen in some of the data presented in this study (for example, the fact that feedback onto *p*_1_ leads to rapid regeneration only at the expense of steady state robustness; Figure 5B). This suggests that even the two-loop OE feedback control system of Figure 5E must have an Achilles' heel somewhere, and indeed this is the case. For such a system to robustly control output, the feedback gain parameters (the relationship between ORN number and the amount of feedback) must be reliable. In essence, sensitivity to one set of parameters (stem cell number, growth rates, death rates, etc.) has been shifted onto another.

Does this mean the control strategy is a failure? Not at all. As engineers know, control is not about eliminating fragility, but managing it. One seeks to transfer fragility to parameters or inputs that are either intrinsically more reliable, or can themselves be controlled by other means, or to outputs in which error is more tolerable. The feedback mechanisms described in the present study end up transferring fragility from cell-intrinsic processes (cell cycle length and death rate) to cell-extrinsic quantities (the level of GDF11 or activin in the extracellular space). This creates an opportunity for additional regulation, as well as opportunities to tie the behavior of cells in the OE neuronal lineage to each other, to other phenomena in the tissue, or even to the behaviors of cells in surrounding tissues. From a systems biology perspective, the present study has defined a control module, whose function can be appreciated in isolation, but whose real utility depends on how it integrates with other modules.

### From Models to Insights

The notion that elements like GDF11, activin, FST, lineage stages, and epithelial architecture are components of an integrated system for controlling growth and regeneration emerges here mainly from the mathematical analysis and computational exploration of models. The models are firmly anchored in experimental data, but their primary use was not to generate experimental predictions (although such things did occur, e.g., Figure 4). “Predictive” modeling can be valuable for testing mechanistic hypotheses, but it often requires a relatively complete picture of a system's components [64,93]. In tissue and organ growth control systems, it is indeed likely that components not considered here—such as Notch and Wnt signaling [94–98], lineage branch points, and other feedback and feedforward factors—also play important roles.

Rather, modeling was used here for its explanatory power, i.e., as a way to achieve clarity in the face of complexity. Whether the precise control mechanisms suggested here are “right” or “wrong” is less important than the fact that they provide a more satisfying set of explanations than those yielded by traditional intuitive reasoning about the data. In the OE, for example, traditional pathway-centered reasoning—following from the analysis of phenotypes—would naturally emphasize the fact that GDF11 and activin are potentially redundant “antineurogenic” factors; that Fst is “proneurogenic,” and that OE growth is somehow regulated by a balance among these factors. Although not inaccurate, this view draws attention away from what may be more fundamental relationships: that Fst extends the dynamic range over which tissue size can be sensed; that GDF11 and activin regulate a cell-fate decision (to replicate or differentiate); and that stem and transit-amplifying cell behaviors can be simple consequences of feedback. Such relationships fit better into the context of observations that Fst is highly expressed adjacent to several other epithelia that respond to Fst-sensitive ligands (e.g., in tongue, eye, and gut [66]; that GDF11 and GDF8 affect other kinds of cell fate decisions (e.g., in retina and muscle [99,100]); and that stem and transit-amplifying cell behaviors are strongly context-dependent in many lineage systems [6,89,90,101].

## Materials and Methods

### OE explant culture and labeling.

OE explants were prepared as previously described [39] and cultured with 10 ng/ml recombinant FGF2 and varying concentrations of GDF11 (PeproTech). After 18 h, bromodeoxyuridine (BrdU) cell-labeling reagent was added at 1:10,000 (#RPN201; Amersham). Two hours later, explants were washed with cold thymidine (10 μm; Sigma-Aldrich), growth factors replenished, and cultures grown for either 16 or 34 h longer (total culture time was either 30 or 48 h). For 48-h cultures, FGF2 and GDF11 were refreshed after 40 h in vitro.

Explants were fixed and stained with rat monoclonal anti-NCAM H28 and mouse monoclonal anti-BrdU antibody as described [39]. Immunoreactivity was visualized with Cy2-Donkey anti-rat IgG (1:50; Jackson Immunoresearch) and Texas Red goat anti-mouse IgG1 (1:50; Jackson Immunoresearch). To compare the percentage of ORNs produced by INPs in each culture condition, total migratory BrdU^+^ cells were counted in at least 15 fields each of duplicate cultures per condition and scored for BrdU and NCAM immunofluorescence by an experimenter blind to the treatment condition, to ensure lack of bias.

### Immunohistochemistry and in situ hybridization to tissue sections.

Embryos were dissected in room temperature phosphate-buffered saline (PBS; pH 7.2) and heads fixed in 4% paraformaldehyde in PBS overnight at 4 °C, then cryoprotected, embedded, sectioned, and processed as described [34]. For *Ngn1* in situ hybridization, tissue was processed using digoxigenin-labeled cRNA probes [34]. FST immunostaining was performed using R&D Systems goat anti-human FST antibody (10 μg/ml final concentration) and visualized with biotinylated horse anti-goat IgG (1:250) in combination with Vector MOM Immunodetection Kit (PK-2200; Vector Labs) according to the manufacturer's instructions.

### Computational methods.

Mathematical analysis and numerical simulation were carried out with the assistance of *Mathematica* (Wolfram Research). Codes used for all cases shown are provided in Protocols S1–S3.

### Accession numbers.

Gene accession numbers used in the manuscript refer to the Mouse Genome Informatics database, http://www.informatics.jax.org/.

## Supporting Information

Protocol S1 Mathematical Appendix File includes Figures S1–S31
Section 1: ODE model of an unbranched lineage.
Section 2: Steady state solution in the absence of feedback.
Section 3: Steady state solution for a two-stage lineage with feedback.
Section 4: Steady state solution for a three-stage lineage with feedback (Figures S1–S3).
Section 5: Final-state solutions in the absence of feedback (Figures S4 and S5).
Section 6: Final-state solutions in the presence of feedback (Figures S6–S11).
Section 7: Time-dependent solutions (Figures S12 and S13).
Section 8: Parameter space exploration—methods.
Section 9: Parameter space exploration—supplemental results (Figures S14–S22).
Section 10: Simulation of pulse-chase experiment (Figure S23).
Section 11: Spatial dynamics calculations (Figures S24–S31).
Section 12: Parameters: definitions, ranges, and justifications.
(1.59 MB PDF)

Protocol S2 Mathematical Appendix, Mathematica Notebook Version This is a version of Protocol S1 that may be opened as an interactive file in Mathematica.
(4.06 MB TXT)

Protocol S3 Mathematical Appendix, Mathematica Player™ Version This is a version of Protocol S1 that may be opened as an interactive file using Mathematica Player freeware, which is available at http://www.wolfram.com/products/player/.
(4.08 MB TXT)

Table S1 Negative Feedback Regulators of Proliferation (104 KB DOC)

## Abbreviations

BrdU: bromodeoxyuridine
FST: follistatin
GDF8: growth and differentiation factor 8
GDF11: growth and differentiation factor 11
INP: immediate neuronal precursor
Ngn1: Neurogenin1
OE: olfactory epithelium
ORN: olfactory receptor neuron
TGFβ: transforming growth factor β
