# Supplementary material for: Cell Lineages and the Logic of Proliferative Control
Source: PLoS Biol. 2009 Jan 20;7(1):e1000015. doi: 10.1371/journal.pbio.1000015 (PMC2628408; doi:10.1371/journal.pbio.1000015)
Supplement: Supplementary file 1 [file pbio.1000015.sd001.pdf]

Supplemental Material

Lander et al., "Cell Lineages and the Logic of Proliferative Control"

1. ODE model of an unbranched lineage

The system of equations in Figure 2b is derived from the principle that the rate of increase of each cell of type "n" has two components, creation by differentiation of a cell of type n-1, and self-replication. The rate of the former will be twice $1 - p_{n-1}$, the factor of two coming from the fact that cell type n-1 produces two cells with every division. The rate of the latter will be twice p_n minus 1; the factor two again reflects the fact that cells produce two cells with each division, while the subtraction of 1 reflects the fact that whenever a cell divides to produce two new cells, one must deduct one for the parental cell that no longer exists.

For cell type zero, the term representing production from a previous lineage stage is omitted. For the cell type at the end of the lineage, which does not divide, the term for replication is omitted. In addition, a probabilistic rate of death is added to the last equation, to capture the fact that the terminal cell often has a limited lifespan. Death of other cell types is not considered here, but could could easily be added to the equations. In addition, at death term that is age-structured, rather than probabilistic, could be used.

In generating simulations of the dynamic behaviors of lineages, it is convenient to perform some rescaling and non-dimensionalize to reduce the numbers of free parameters. For example, it is useful to define a unit of time $\tau = \mathrm{t}\ v_1$; a parameter $\zeta = v_0 / v_1$is used to eliminate v_0;and a parameter $\delta = d / v_1$is also defined. Here is how this works out for a three stage lineage.

```
{χ0'[t] == (2 p0 - 1) v0 χ0[t],
   χ1'[t] == 2 (1 - p0) v0 χ0[t] + (2 p1 - 1) v1 χ1[t],
   χ2'[t] == 2 (1 - p1) v1 χ1[t] - d χ2[t]} /.
  {v0 → v1 ζ, d → δ v1, χn_'[t] → v1 χn'[τ], χn_[t] → χn[τ]} // TableForm
```

$$v_1\,\chi_0'[\tau] == \zeta\,(-1 + 2\,p_0)\,v_1\,\chi_0[\tau]$$

$$v_1\,\chi_1'[\tau] == 2\,\zeta\,(1 - p_0)\,v_1\,\chi_0[\tau] + (-1 + 2\,p_1)\,v_1\,\chi_1[\tau]$$

$$v_1\,\chi_2'[\tau] == 2\,(1 - p_1)\,v_1\,\chi_1[\tau] - \delta\,v_1\,\chi_2[\tau]$$

v_1 can be cancelled from all sides of this. The remaining parameters are therefore p_0 , p_1, δ and ζ.

2. Steady state solution in the absence of feedback

The system in Fig. 2 b may be solved in the steady state by setting all time rates to zero.

From the first equation one gets $\chi_0 == 0$ or $v_0 == 0$ or $p_0 == 0.5$. The only solution of interest is $p_0 == 0.5$, which in turn implies that χ_0 is undetermined, i.e. arbitrary

From the last equation one gets

$$\chi_n == -\frac{2\,(-1 + p_2)\,v_2\,\chi_{-1+n}}{d}$$

From every other equation one gets

$$\forall_{j>0,\,j<n}\,,\quad \chi_{n-j} == \frac{2\left(-1 + p_{n-j-1}\right)v_{n-j-1}\,\chi_{n-j-1}}{\left(-1 + 2\,p_{n-j}\right)v_{n-j}}$$

Putting these together implies

$$\chi_n == \frac{\chi_0 v_0}{d} \prod_{i=1}^{n-1} \frac{2(1-p_i)}{1-2p_i}$$

3. Steady state solution for a two stage lineage with feedback

In this study, feedback is represented by multiplying p- and v- parameters by Hill functions of the form

$$\frac{1}{1+(a\,\chi_{\text{final}}[t])^n}$$

where $\chi_{\text{final}}[t]$ represents the amount of terminal stage cells, and a is a parameter. For a two stage lineage, with just a stem cell and a terminal stage cell, we thus may write, in the steady state:

twostage =

$$\{0 == (-1+2\,p_0)\,v_0\,\chi_0[t],\ 0 == 2\,(1-p_0)\,v_0\,\chi_0[t] - d\,\chi_1[t]\}\ /.\ \left\{p_0 \to \frac{p_0}{1+(j\,\chi_1[t])^n},\ v_0 \to \frac{v_0}{1+(k\,\chi_1[t])^n}\right\}$$

$$\left\{0 == \frac{v_0\,\chi_0[t]\left(-1+\frac{2\,p_0}{1+(j\,\chi_1[t])^n}\right)}{1+(k\,\chi_1[t])^n},\ 0 == -d\,\chi_1[t] + \frac{2\,v_0\,\chi_0[t]\left(1-\frac{p_0}{1+(j\,\chi_1[t])^n}\right)}{1+(k\,\chi_1[t])^n}\right\}$$

If j≠0, and n=1, we may solve this as follows.

Solve[twostage /. n → 1, {χ0[t], χ1[t]}] // Simplify

$$\left\{\{\chi_0[t]\to 0,\ \chi_1[t]\to 0\},\ \left\{\chi_0[t]\to \frac{d\,(-1+2\,p_0)\,(j-k+2\,k\,p_0)}{j^2\,v_0},\ \chi_1[t]\to \frac{-1+2\,p_0}{j}\right\}\right\}$$

Notice there are two solutions, the first being the trivial solution in which both cell types have a level of zero. This is the state reached iff $p_0 < 0.5$

If j≠0 and n=2, the solution is:

Solve[twostage /. n → 2, {χ0[t], χ1[t]}] // Simplify

$$\left\{\{\chi_0[t]\to 0,\ \chi_1[t]\to 0\},\ \left\{\chi_0[t]\to \frac{d\,\sqrt{-1+2\,p_0}\,(-j^2+k^2-2\,k^2\,p_0)}{j^3\,v_0},\ \chi_1[t]\to -\frac{\sqrt{-1+2\,p_0}}{j}\right\},\right.$$
$$\left.\left\{\chi_0[t]\to \frac{d\,\sqrt{-1+2\,p_0}\,(j^2-k^2+2\,k^2\,p_0)}{j^3\,v_0},\ \chi_1[t]\to \frac{\sqrt{-1+2\,p_0}}{j}\right\}\right\}$$

Of these solutions, the second may be ignored as it gives values of $\chi_1$that are always negative.

If j≠0 and n=1/2, the solution is:

Solve[twostage /. n → 1 / 2, {χ0[t], χ1[t]}] // Simplify

$$\left\{\{\chi_0[t]\to 0,\ \chi_1[t]\to 0\},\ \left\{\chi_0[t]\to \frac{d\left(1+\sqrt{\frac{k\,(1-2\,p_0)^2}{j}}\right)(1-2\,p_0)^2}{j\,v_0},\ \chi_1[t]\to \frac{(1-2\,p_0)^2}{j}\right\}\right\}$$

If j=0, then $p_0$must be 0.5 and $\chi_0$becomes arbitrary (as it does when there is no feeback). In this case, we may drop the first equation, and solve the second one in terms of χ_0. Here we look at the case where n=1

twostage[[2]]

$$0 == -d\,\chi_1[t] + \frac{2\,v_0\,\chi_0[t]\left(1-\frac{p_0}{1+(j\,\chi_1[t])^n}\right)}{1+(k\,\chi_1[t])^n}$$

```
Assuming[d > 0 && v₀ > 0 && χ₀ > 0 && k ≥ 0 && χ₁[t] ≥ 0,
 Simplify[Solve[twostage[[2]] /. {j → 0, p₀ → 1/2, n → 1, χ₀[t] → χ₀}, χ₁[t]]]]
```

$$\left\{\left\{\chi_1[t] \to -\frac{d + \sqrt{d\,(d + 4\,k\,v_0\,\chi_0)}}{2\,d\,k}\right\}, \left\{\chi_1[t] \to \frac{-1 + \sqrt{1 + \frac{4\,k\,v_0\,\chi_0}{d}}}{2\,k}\right\}\right\}$$

Of these solutions it is easy to see that only the second one can be positive-valued. Below is the solution when n=2

```
Assuming[d > 0 && v₀ > 0 && χ₀ > 0 && k ≥ 0 && χ₁[t] ≥ 0,
 Simplify[Solve[twostage[[2]] /. {j → 0, p₀ → 1/2, n → 2, χ₀[t] → χ₀}, χ₁[t]]]]
```

$$\left\{\left\{\chi_1[t] \to \frac{-2\,3^{1/3}\,d^2\,k^2 + 2^{1/3}\left(9\,d^2\,k^4\,v_0\,\chi_0 + \sqrt{12\,d^6\,k^6 + 81\,d^4\,k^8\,v_0^2\,\chi_0^2}\right)^{2/3}}{6^{2/3}\,d\,k^2\left(9\,d^2\,k^4\,v_0\,\chi_0 + \sqrt{12\,d^6\,k^6 + 81\,d^4\,k^8\,v_0^2\,\chi_0^2}\right)^{1/3}}\right\},\right.$$

$$\left\{\chi_1[t] \to \frac{2\left(3\,i + \sqrt{3}\right)d^2\,k^2 + i\,2^{1/3}\,3^{1/6}\left(i + \sqrt{3}\right)\left(9\,d^2\,k^4\,v_0\,\chi_0 + \sqrt{12\,d^6\,k^6 + 81\,d^4\,k^8\,v_0^2\,\chi_0^2}\right)^{2/3}}{2\,2^{2/3}\,3^{5/6}\,d\,k^2\left(9\,d^2\,k^4\,v_0\,\chi_0 + \sqrt{12\,d^6\,k^6 + 81\,d^4\,k^8\,v_0^2\,\chi_0^2}\right)^{1/3}}\right\},$$

$$\left.\left\{\chi_1[t] \to \frac{2\left(-3\,i + \sqrt{3}\right)d^2\,k^2 + 2^{1/3}\,3^{1/6}\left(-1 - i\,\sqrt{3}\right)\left(9\,d^2\,k^4\,v_0\,\chi_0 + \sqrt{12\,d^6\,k^6 + 81\,d^4\,k^8\,v_0^2\,\chi_0^2}\right)^{2/3}}{2\,2^{2/3}\,3^{5/6}\,d\,k^2\left(9\,d^2\,k^4\,v_0\,\chi_0 + \sqrt{12\,d^6\,k^6 + 81\,d^4\,k^8\,v_0^2\,\chi_0^2}\right)^{1/3}}\right\}\right\}$$

4. Steady state solution for a three - stage lineage with feedback (Fig. S1-S3)

```
threestage =
 {0 == (2 p₀ - 1) v₀ χ₀[t],
   0 == 2 (1 - p₀) v₀ χ₀[t] + (2 p₁ - 1) v₁ χ₁[t],
   0 == 2 (1 - p₁) v₁ χ₁[t] - d χ₂[t]} /.
  {p₀ → p₀/(1 + (j χ₂[t])^n), v₀ → v₀/(1 + (k χ₂[t])^n), p₁ → p₁/(1 + (g χ₂[t])^n), v₁ → v₁/(1 + (h χ₂[t])^n)}
```

$$\left\{0 == \frac{v_0\,\chi_0[t]\left(-1 + \frac{2\,p_0}{1+(j\,\chi_2[t])^n}\right)}{1 + (k\,\chi_2[t])^n},\right.$$

$$\left.0 == \frac{v_1\,\chi_1[t]\left(-1 + \frac{2\,p_1}{1+(g\,\chi_2[t])^n}\right)}{1 + (h\,\chi_2[t])^n} + \frac{2\,v_0\,\chi_0[t]\left(1 - \frac{p_0}{1+(j\,\chi_2[t])^n}\right)}{1 + (k\,\chi_2[t])^n},\ 0 == -d\,\chi_2[t] + \frac{2\,v_1\,\chi_1[t]\left(1 - \frac{p_1}{1+(g\,\chi_2[t])^n}\right)}{1 + (h\,\chi_2[t])^n}\right\}$$

If $j \neq 0$, and $n = 1$, we may solve this as follows.

```
Solve[threestage /. n → 1, {χ₀[t], χ₁[t], χ₂[t]}] // FullSimplify
```

$$\left\{\{\chi_0[t] \to 0,\ \chi_1[t] \to 0,\ \chi_2[t] \to 0\},\ \left\{\chi_0[t] \to 0,\ \chi_1[t] \to \frac{d\,(-1 + 2\,p_1)\,(g - h + 2\,h\,p_1)}{g^2\,v_1},\ \chi_2[t] \to \frac{-1 + 2\,p_1}{g}\right\},\right.$$

$$\left\{\chi_0[t] \to \frac{d\,(-1 + 2\,p_0)\,(j - k + 2\,k\,p_0)\,(g - j - 2\,g\,p_0 + 2\,j\,p_1)}{2\,j^2\,(g - j - 2\,g\,p_0 + j\,p_1)\,v_0},\right.$$

$$\left.\left.\chi_1[t] \to \frac{d\,(-1 + 2\,p_0)\,(-g + j + 2\,g\,p_0)\,(-h + j + 2\,h\,p_0)}{2\,j^2\,(-g + j + 2\,g\,p_0 - j\,p_1)\,v_1},\ \chi_2[t] \to \frac{-1 + 2\,p_0}{j}\right\}\right\}$$

$$\left\{\{\chi_0[t]\to 0,\ \chi_1[t]\to 0,\ \chi_2[t]\to 0\},\ \left\{\chi_0[t]\to 0,\ \chi_1[t]\to \frac{d\,(-1+2\,p_1)\,(g-h+2\,h\,p_1)}{g^2\,v_1},\ \chi_2[t]\to \frac{-1+2\,p_1}{g}\right\},\right.$$

$$\left\{\chi_0[t]\to \frac{d\,(-1+2\,p_0)\,(j-k+2\,k\,p_0)\,(g-j-2\,g\,p_0+2\,j\,p_1)}{2\,j^2\,(g-j-2\,g\,p_0+j\,p_1)\,v_0},\right.$$

$$\left.\left.\chi_1[t]\to \frac{d\,(-1+2\,p_0)\,(-g+j+2\,g\,p_0)\,(-h+j+2\,h\,p_0)}{2\,j^2\,(-g+j+2\,g\,p_0-j\,p_1)\,v_1},\ \chi_2[t]\to \frac{-1+2\,p_0}{j}\right\}\right\}$$

As before, the trivial steady state (all zeros) is reached for $p_0 < 0.5$.

Notice that are now two possible non − trivial steady state solutions for χ_2, either $\frac{2\,p_0-1}{j}$ or $\frac{2\,p_1-1}{g}$. The latter solution is accompanied by χ_0 going to zero. Thus, for some parameter values, χ_0 behaves like *a* stem cell and χ_1 like *a* transit amplifying cell, and for other parameter values, χ_0 is extinguished and χ_1 takes on the behavior of *a* stem cell.

Which steady state is reached depends on whether $\left(\frac{2\,p_1-1}{g}\right) > \left(\frac{2\,p_0-1}{j}\right)$. If this is true, the steady state with $\chi_2 = \frac{2\,p_1-1}{g}$ is reached.

Note that, since $p_0 > 0.5$, this condition will never be met for cases where $p_1 < 0.5$. So the ability of χ_1 to take over as the stem cell depends upon $p_1 > 0.5$.

If $j = 0$, or $n > 1$, we may use the same approach to obtain steady state solutions, although the expressions for them become more complicated. Here let' *s* just look at the case, discussed in the main text, where there is feedback on p_1 and v_1, but not on p_0 or v_0.

To make the solution simpler, let us make some substitions to nondimensionalize :

Let c1 $= \chi_1[t]/\chi_0$; c2 $= \chi_2[t]/\chi_0$; $\zeta = v_0/v_1$; $\gamma = g\,\chi_0$; $\eta = h\,\chi_0$; $\delta = d/v_1$

```
threestage[[{2, 3}]] /. {n → 1, j → 0, k → 0, p₀ → 1 / 2, χ₀[t] → χ₀,
   χ₁[t] → c1 χ₀, χ₂[t] → c2 χ₀, v₀ → v₁ ξ, g → γ / χ₀, h → η / χ₀, d → δ v₁} // Simplify
```

$$\left\{\left(\xi+\frac{c1\left(-1+\frac{2\,p_1}{1+c2\,\gamma}\right)}{1+c2\,\eta}\right)v_1\,\chi_0 == 0,\ \left(-c2\,\delta+\frac{2\,c1\left(1-\frac{p_1}{1+c2\,\gamma}\right)}{1+c2\,\eta}\right)v_1\,\chi_0 == 0\right\}$$

```
Solve[%, {c1, c2}] // FullSimplify
```

$$\left\{\left\{c1\to \frac{1}{2\,\gamma^2\,\delta}\left(\delta^2\,\eta+2\,\gamma^2\,\xi^2\,\eta+\gamma\,\delta\,(-\delta+\xi\,\eta)+4\,\delta^2\,\eta\,p_1^2+(\delta\,\eta-\gamma\,(\delta+\xi\,\eta))\sqrt{(\delta+2\,\gamma\,\xi)^2+4\,\delta^2\,(-1+p_1)\,p_1}+\right.\right.\right.$$
$$\left.2\,\delta\,p_1\left(\gamma\,(\delta+\xi\,\eta)-\eta\left(2\,\delta+\sqrt{(\delta+2\,\gamma\,\xi)^2+4\,\delta^2\,(-1+p_1)\,p_1}\right)\right)\right),$$
$$\left.c2\to -\frac{\delta-2\,\gamma\,\xi-2\,\delta\,p_1+\sqrt{(\delta+2\,\gamma\,\xi)^2+4\,\delta^2\,(-1+p_1)\,p_1}}{2\,\gamma\,\delta}\right\},\ \left\{c1\to\right.$$
$$\frac{1}{2\,\gamma^2\,\delta}\left(\delta^2\,\eta+2\,\gamma^2\,\xi^2\,\eta+\gamma\,\delta\,(-\delta+\xi\,\eta)+4\,\delta^2\,\eta\,p_1^2+(-\delta\,\eta+\gamma\,(\delta+\xi\,\eta))\sqrt{(\delta+2\,\gamma\,\xi)^2+4\,\delta^2\,(-1+p_1)\,p_1}+\right.$$
$$\left.2\,\delta\,p_1\left(\gamma\,(\delta+\xi\,\eta)+\eta\left(-2\,\delta+\sqrt{(\delta+2\,\gamma\,\xi)^2+4\,\delta^2\,(-1+p_1)\,p_1}\right)\right)\right),$$
$$\left.\left.c2\to \frac{-\delta+2\,\gamma\,\xi+2\,\delta\,p_1+\sqrt{(\delta+2\,\gamma\,\xi)^2+4\,\delta^2\,(-1+p_1)\,p_1}}{2\,\gamma\,\delta}\right\}\right\}$$

Notice that η does not enter into the steady state for c2, because, as was shown in the case without feedback, v_1 does not affect the steady state for c2.

We may re –

write the two solutions for c2 as $\frac{-\delta + 2\gamma\zeta + 2\delta p_1 - \sqrt{(\delta + 2\gamma\zeta)^2 + 4\delta^2(-1+p_1)p_1}}{2\gamma\delta}$ and $\frac{-\delta + 2\gamma\zeta + 2\delta p_1 + \sqrt{(\delta + 2\gamma\zeta)^2 + 4\delta^2(-1+p_1)p_1}}{2\gamma\delta}$

The expression inside the radical may be re – written as $(\delta(2p_1 - 1) + 2\gamma\zeta)^2 +$
$8\gamma\delta\zeta(1 - p_1)$. The expression outside the radical may be re – written as $\delta(2p_1 - 1) + 2\gamma\zeta$. Given that $1 - p_1 > 0$,

then the square root of the expression inside the radical is always greater than $\sqrt{(\delta(2p_1 - 1) + 2\gamma\zeta)^2}$. Therefore, if $\delta(2p_1 - 1) + 2\gamma\zeta > 0$,
the first solution leads to *a* negative c2 because the subtraction from *a* positive number of *a* larger positive number will lead to *a* negative one. If $\delta(2p_1 - 1) +$
$2\gamma\zeta < 0$, then the first solution leads to negative c2 because *a* negative
number if added to *a* negative number. Accordingly, the second solution is the correct one.

$$\textbf{sol1} = \Big\{\textbf{c1} \to \frac{1}{2\gamma^2\delta}\Big(\delta^2\eta + 2\gamma^2\zeta^2\eta + \gamma\delta(-\delta + \zeta\eta) + 4\delta^2\eta p_1^2 + (-\delta\eta + \gamma(\delta + \zeta\eta))\sqrt{(\delta + 2\gamma\zeta)^2 + 4\delta^2(-1+p_1)p_1} + 2\delta p_1\Big(\gamma(\delta + \zeta\eta) + \eta\Big(-2\delta + \sqrt{(\delta + 2\gamma\zeta)^2 + 4\delta^2(-1+p_1)p_1}\Big)\Big)\Big),$$
$$\textbf{c2} \to \frac{-\delta + 2\gamma\zeta + 2\delta p_1 + \sqrt{(\delta + 2\gamma\zeta)^2 + 4\delta^2(-1+p_1)p_1}}{2\gamma\delta}\Big\};$$

In order to calculate the robustness of c2 to the underlying parameters, we need to restore some of the original (dimensional) parameters

$$\textbf{sol1[[2]] /. }\{\gamma \to g\chi_0,\ c2 \to \chi_2/\chi_0,\ \zeta \to v_0/v_1,\ \delta \to d/v_1\}\ \textbf{// Simplify}$$

$$\frac{\chi_2}{\chi_0} \to \frac{-d + 2\,d\,p_1 + 2\,g\,v_0\,\chi_0 + v_1\sqrt{\frac{-4d^2p_1 + 4d^2p_1^2 + (d + 2g v_0\chi_0)^2}{v_1^2}}}{2\,d\,g\,\chi_0}$$

We may rearrange this to see that g $v_0\,\frac{\chi_0}{d}$ all lump together as a single parameter, which we may call ω. Thus we have

$$\textbf{Assuming}\Big[d > 0,\ \textbf{FullSimplify}\Big[\chi_2 == \frac{-d + 2dp_1 + 2gv_0\chi_0 + \sqrt{-4d^2p_1 + 4d^2p_1^2 + (d + 2gv_0\chi_0)^2}}{2dg}\ \textbf{/. }\chi_0 \to \frac{\omega d}{g v_0}\Big]\Big]$$

$$\chi_2 == \frac{-1 + 2\omega + 2p_1 + \sqrt{(1 + 2\omega)^2 + 4(-1+p_1)p_1}}{2g}$$

Now we can see right away if $2\ \omega << -1 + 2\ p_1$ (which requires $p_1 > 0.5$) then that guarantees $2\omega << 1$, which means χ_2->
$\frac{-1+2\ p_1+\sqrt{4\ p_1\ (-1+p_1)+1}}{2\ g}$ which simplifies to $\frac{2p_1 - 1}{g}$. So it follows that for $2\ \omega << -1 + 2\ p_1$, the system can be arbitrarily robust to ω, i.e. to v_0, χ_0, and d.
We can also show this by defining sensitivity according to its usual meaning in engineering:

$$\textbf{Sen[y\_, x\_] := D[y, x]}\ \frac{x}{y}$$

$$\textbf{Assuming}\Big[g > 0,\ \textbf{FullSimplify}\Big[\textbf{Sen}\Big[\frac{-1 + 2\omega + 2p_1 + \sqrt{(1 + 2\omega)^2 + 4(-1+p_1)p_1}}{2g},\ \omega\Big]\Big]\Big]$$

$$\frac{\omega\left(2 + \frac{2+4\omega}{\sqrt{(1+2\omega)^2 + 4(-1+p_1)p_1}}\right)}{-1 + 2\omega + 2p_1 + \sqrt{(1 + 2\omega)^2 + 4(-1+p_1)p_1}}$$

```
Plot3D[ω (2 + (2+4 ω)/Sqrt[(1+2 ω)^2+4 (-1+p1) p1]) / (-1 + 2 ω + 2 p1 + Sqrt[(1 + 2 ω)^2 + 4 (-1 + p1) p1]), {p1, 0, 1}, {ω, 0, 1}, PlotRange → {0, 1},
 AxesLabel → {"p1", "ω", "sensitivity to ω"}, PlotLabel → Style["Figure S1", Bold, Large]]
```

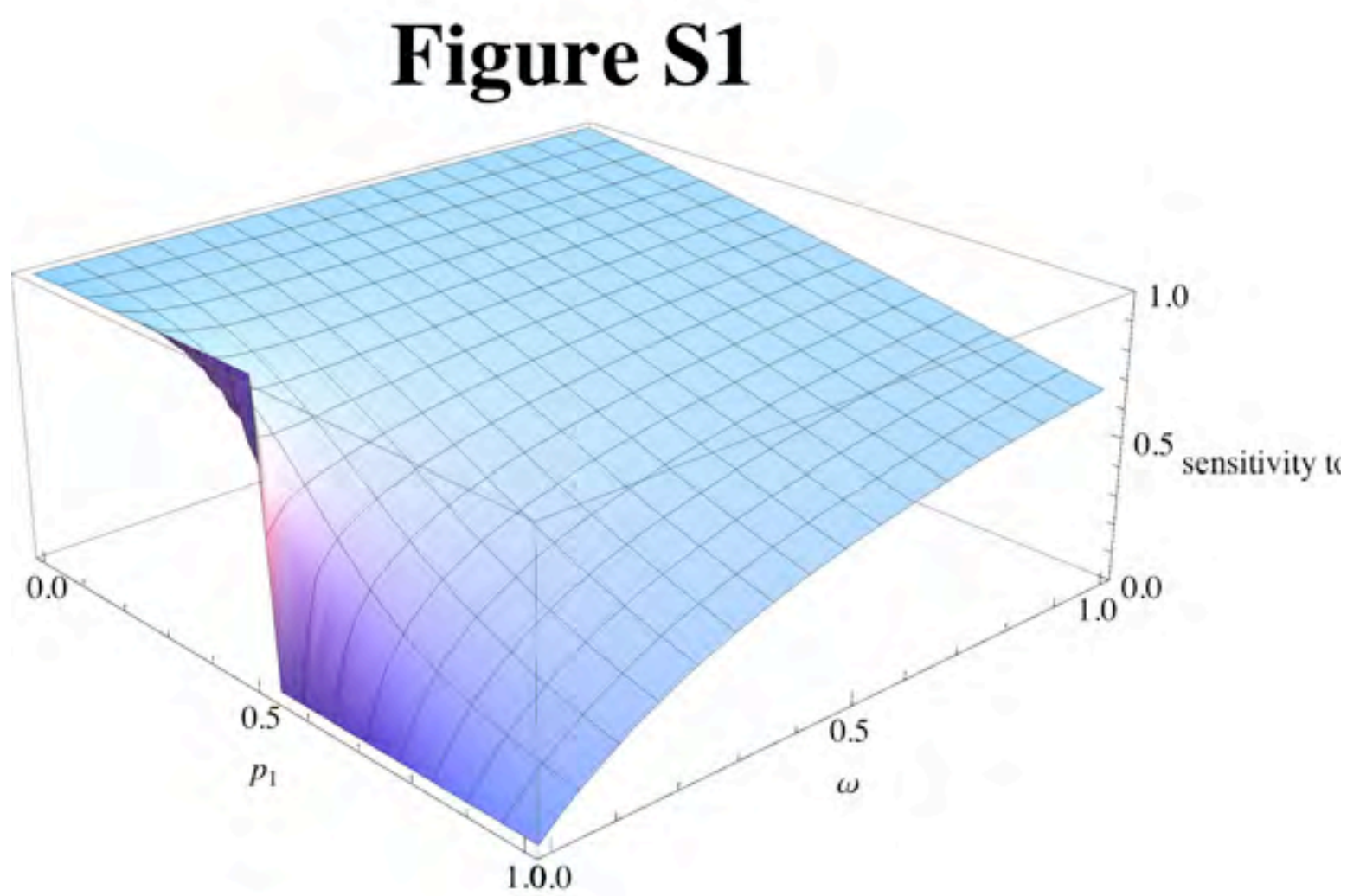

So sensitivity to v_0, χ_0, and d can indeed be made arbitrariy small for $p_1 > 0.5$ and ω<<1. While we're at it we can also examine the sensitivity to p_1

```
Plot3D[Evaluate[Sen[(-1 + 2 ω + 2 p1 + Sqrt[(1 + 2 ω)^2 + 4 (-1 + p1) p1])/(2 g), p1]], {p1, 0, 1},
 {ω, 0, 1}, PlotRange → {0, 3}, AxesLabel → {"p1", "ω", "sensitivity to p1"},
 PlotLabel → Style["Figure S2", Bold, Large]]
```

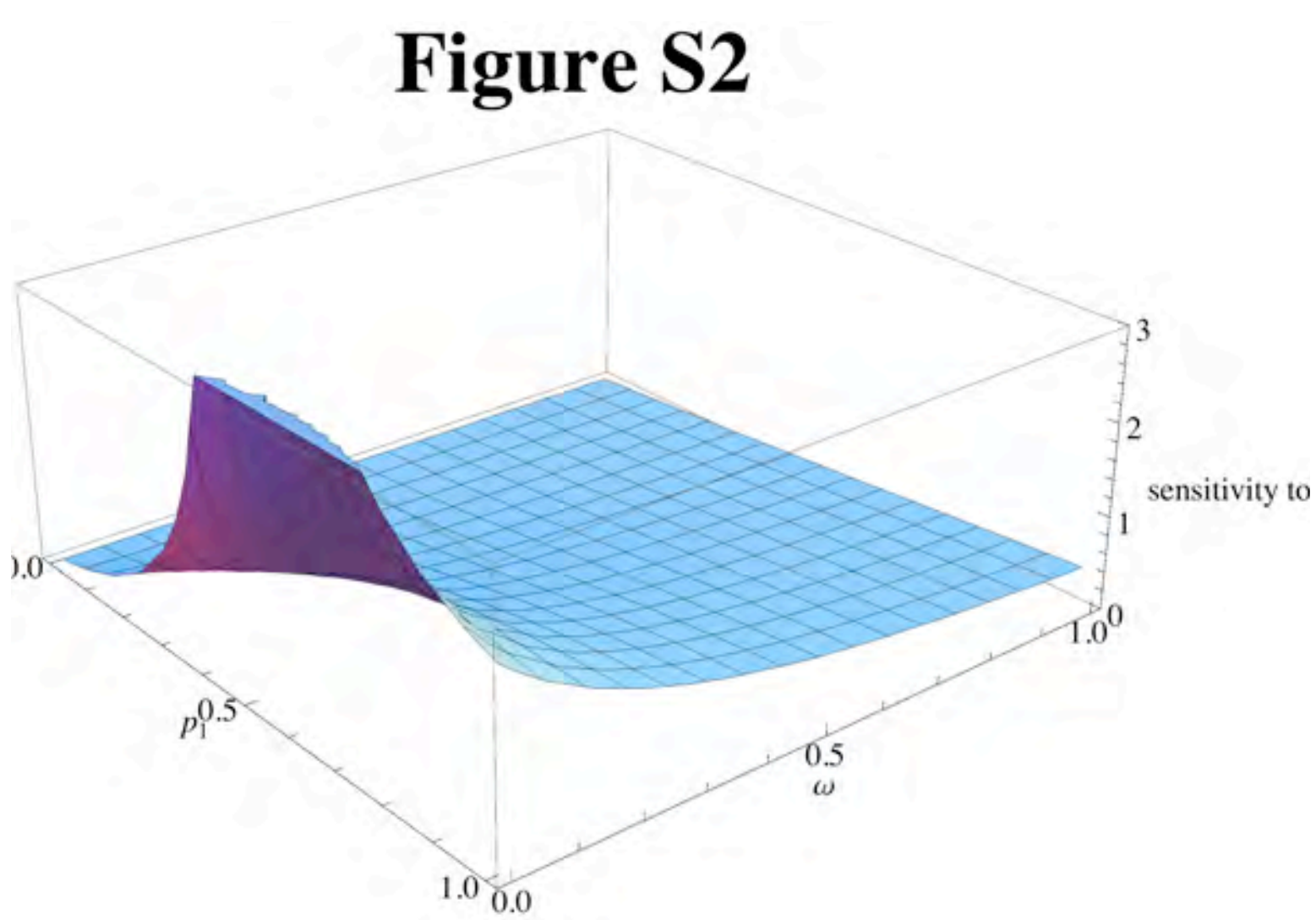

To visualize sensitivity to g, we need replace ω with α g (since ω is a function of g), then calculate sensitivity to g, then resubstitute α g with ω

```
Assuming[g > 0,
  FullSimplify[Sen[(-1 + 2 ω + 2 p1 + Sqrt[(1 + 2 ω)^2 + 4 (-1 + p1) p1])/(2 g) /. ω → α g, g]]] /. g → ω / α
```

$$\frac{1 + 2\,\omega - 2\,p_1 - \sqrt{(1 + 2\,\omega)^2 + 4\,(-1 + p_1)\,p_1}}{2\,\sqrt{(1 + 2\,\omega)^2 + 4\,(-1 + p_1)\,p_1}}$$

```
Plot3D[(1 + 2 ω - 2 p1 - Sqrt[(1 + 2 ω)^2 + 4 (-1 + p1) p1])/(2 Sqrt[(1 + 2 ω)^2 + 4 (-1 + p1) p1]), {p1, 0, 1}, {ω, 0, 1}, PlotRange → All,
 AxesLabel → {"p1", "ω", "sensitivity to g"}, PlotLabel → Style["Figure S3", Bold, Large]]
```

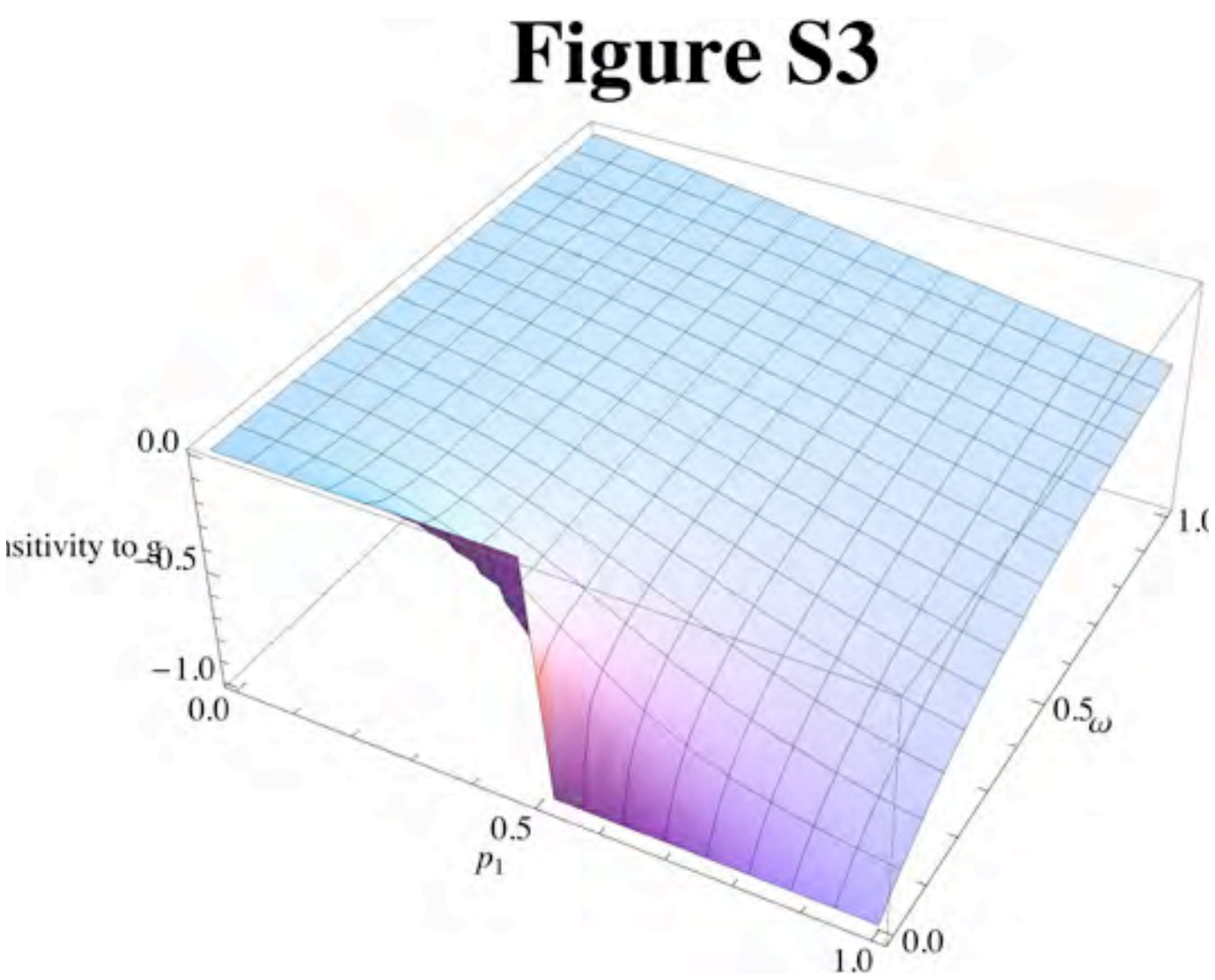

So low sensitivity to ω comes at the expense of linear sensitivity to g.

5. Final - state solutions in the absence of feedback (Fig. S4-S5)

- ***Consider the time dependent solution for a two stage system, with initial conditions of $\chi_0 = \chi_{init}$ and $\chi_1 = 0$, and a constant $p_0 < 0.5$ and no death of the terminal cell***

```
system1 = {χ0'[t] == (-1 + 2 p0) v0 χ0[t], χ1'[t] == 2 (1 - p0) v0 χ0[t], χ0[0] == χinit, χ1[0] == 0};
```

To simplify things, let's define a time scale $\tau = t\,v_0$. then $dt = \frac{1}{v_0}\,d\tau$.

```
system2 = Assuming[v0 > 0, Simplify[system1 /. {χn_'[t] → v0 χn'[τ], χn_[t] → χn[τ]}]]
```

$\{(-1 + 2\,p_0)\,\chi_0[\tau] == \chi_0'[\tau],\ 2\,(-1 + p_0)\,\chi_0[\tau] + \chi_1'[\tau] == 0,\ \chi_{init} == \chi_0[0],\ \chi_1[0] == 0\}$

Solving the differential equation directly:

```
DSolve[system2, {χ0[τ], χ1[τ]}, τ] // FullSimplify
```

$$\left\{\left\{\chi_0[\tau] \to e^{\tau\,(-1+2\,p_0)}\,\chi_{init},\ \chi_1[\tau] \to \frac{2\,\left(-1 + e^{\tau\,(-1+2\,p_0)}\right)\,(-1 + p_0)\,\chi_{init}}{1 - 2\,p_0}\right\}\right\}$$

We may evaluate $\chi_1[\tau]$as τ goes to infinity to get the total number of terminal cells produced.

$$\textbf{Assuming}\left[p_0 < \frac{1}{2},\ \textbf{Limit}\left[\frac{2\left(-1 + e^{\tau(-1+2p_0)}\right)(-1 + p_0)\,\chi_{init}}{1 - 2p_0},\ \tau \to \infty\right]\right]$$

$$\frac{2(-1 + p_0)\,\chi_{init}}{-1 + 2p_0}$$

We may then calculate the sensitivity of this output to p_0.

$$\sigma = \textbf{Sen}\left[\frac{2(-1 + p_0)\,\chi_{init}}{-1 + 2p_0},\ p_0\right] \mathbin{//} \textbf{Simplify}$$

$$\frac{p_0}{1 - 3p_0 + 2p_0^2}$$

It is useful to expess this number in terms of the amplification factor, "a" i.e. the final number of cells relative to the starting pool of progenitors. As we can see from the expression for output, this factor is $2(p_0 - 1)/(2p_0 - 1)$. Thus, we may express $p_0$in terms of a

$$\textbf{Solve}\left[a == \frac{2(-1 + p_0)}{-1 + 2p_0},\ p_0\right] \mathbin{//} \textbf{Simplify}$$

$$\left\{\left\{p_0 \to \frac{-2 + a}{2(-1 + a)}\right\}\right\}$$

Thus for a 1000 fold amplification, $p_0$needs to be $\frac{998}{2(999)}$ or 0.4995

$$\sigma\ /.\ p_0 \to \frac{-2 + a}{2(-1 + a)} \mathbin{//} \textbf{Simplify}$$

$$-3 + \frac{2}{a} + a$$

From this we can immediately see that, for values of the amplification factor above 10, the sensitivity of output to $p_0$is approximately equal to the amplification factor itself (i.e. enormous!)

- ***Now let's consider the case where $p_0$is not a constant, but undergoes a linear decline over time, from a starting value of pmax to an ending value of pmin, at time tmax. As before we will define our time scales in terms of v_0, and replace t with τ. As long as pmin<0.5, the system will reach a final state***

$$\textbf{system3} = \textbf{system2}\ /.\ p_0 \to (\text{pmax} - \text{pmin})\left(1 - \frac{\tau}{\tau\text{max}}\right) + \text{pmin}$$

$$\left\{\left(-1 + 2\left(\text{pmin} + (\text{pmax} - \text{pmin})\left(1 - \frac{\tau}{\tau\text{max}}\right)\right)\right)\chi_0[\tau] == \chi_0'[\tau],\right.$$
$$\left. 2\left(-1 + \text{pmin} + (\text{pmax} - \text{pmin})\left(1 - \frac{\tau}{\tau\text{max}}\right)\right)\chi_0[\tau] + \chi_1'[\tau] == 0,\ \chi_{init} == \chi_0[0],\ \chi_1[0] == 0\right\}$$

$$\textbf{DSolve}[\textbf{system3},\ \{\chi_0[\tau],\ \chi_1[\tau]\},\ \tau] \mathbin{//} \textbf{Flatten} \mathbin{//} \textbf{FullSimplify}$$

$$\left\{\chi_0[\tau] \to e^{\frac{\tau(-\text{pmax}\,\tau + \text{pmin}\,\tau - \tau\text{max} + 2\,\text{pmax}\,\tau\text{max})}{\tau\text{max}}}\chi_{init},\ \chi_1[\tau] \to\right.$$
$$\frac{1}{2(\text{pmax} - \text{pmin})}\left(-2\left(-1 + e^{\tau\left(-1 + 2\,\text{pmax} + \frac{(-\text{pmax} + \text{pmin})\tau}{\tau\text{max}}\right)}\right)(\text{pmax} - \text{pmin}) + e^{\frac{(1 - 2\,\text{pmax})^2\,\tau\text{max}}{4(\text{pmax} - \text{pmin})}}\sqrt{\pi}\sqrt{(-\text{pmax} + \text{pmin})\,\tau\text{max}}\right.$$
$$\left.\left.\left(\text{Erfi}\left[\frac{-2\,\text{pmin}\,\tau + 2\,\text{pmax}\,(\tau - \tau\text{max}) + \tau\text{max}}{2\sqrt{(-\text{pmax} + \text{pmin})\,\tau\text{max}}}\right] - \text{Erfi}\left[\frac{\tau\text{max} - 2\,\text{pmax}\,\tau\text{max}}{2\sqrt{(-\text{pmax} + \text{pmin})\,\tau\text{max}}}\right]\right)\right)\chi_{init}\right\}$$

To find the final state, we see how χ_1 behaves as τ goes to infinity.

$$\mathbf{sol3 = Assuming}\Big[\mathbf{pmax < 1 \;\&\&\; pmin < \frac{1}{2} \;\&\&\; pmax > pmin \;\&\&\; \tau max > 1 \;\&\&\; \chi_{init} > 0,}$$

$$\mathbf{Limit}\Big[\frac{1}{2\,(\mathrm{pmax}-\mathrm{pmin})}\Big(-2\left(-1+e^{\tau\left(-1+2\,\mathrm{pmax}+\frac{(-\mathrm{pmax}+\mathrm{pmin})\,\tau}{\tau\mathrm{max}}\right)}\right)(\mathrm{pmax}-\mathrm{pmin})+$$

$$e^{\frac{(1-2\,\mathrm{pmax})^2\,\tau\mathrm{max}}{4\,(\mathrm{pmax}-\mathrm{pmin})}}\sqrt{\pi}\,\sqrt{(-\mathrm{pmax}+\mathrm{pmin})\,\tau\mathrm{max}}\,\Big(\mathrm{Erfi}\Big[\frac{-2\,\mathrm{pmin}\,\tau+2\,\mathrm{pmax}\,(\tau-\tau\mathrm{max})+\tau\mathrm{max}}{2\sqrt{(-\mathrm{pmax}+\mathrm{pmin})\,\tau\mathrm{max}}}\Big]-$$

$$\mathrm{Erfi}\Big[\frac{\tau\mathrm{max}-2\,\mathrm{pmax}\,\tau\mathrm{max}}{2\sqrt{(-\mathrm{pmax}+\mathrm{pmin})\,\tau\mathrm{max}}}\Big]\Big)\Big)\,\chi_{init},\;\; \tau \to \infty\Big]\Big] \;//\; \mathbf{Simplify}$$

$$\frac{1}{2\,(\mathrm{pmax}-\mathrm{pmin})}\Big(2\,\mathrm{pmax}-2\,\mathrm{pmin}+e^{\frac{(1-2\,\mathrm{pmax})^2\,\tau\mathrm{max}}{4\,(\mathrm{pmax}-\mathrm{pmin})}}\sqrt{\pi}\,\sqrt{(\mathrm{pmax}-\mathrm{pmin})\,\tau\mathrm{max}}\;-$$

$$e^{\frac{(1-2\,\mathrm{pmax})^2\,\tau\mathrm{max}}{4\,(\mathrm{pmax}-\mathrm{pmin})}}\sqrt{\pi}\,\sqrt{(\mathrm{pmax}-\mathrm{pmin})\,\tau\mathrm{max}}\;\mathrm{Erf}\Big[\frac{\tau\mathrm{max}-2\,\mathrm{pmax}\,\tau\mathrm{max}}{2\sqrt{(\mathrm{pmax}-\mathrm{pmin})\,\tau\mathrm{max}}}\Big]\Big)\,\chi_{init}$$

In the special case of p going from 1 to zero, this expression is considerably more compact

$$\mathbf{sol4 = sol3\;/.\;\{pmax \to 1,\;\; pmin \to 0\}\;//\;FullSimplify}$$

$$\frac{1}{2}\left(2+e^{\tau\mathrm{max}/4}\sqrt{\pi}\,\sqrt{\tau\mathrm{max}}\left(1+\mathrm{Erf}\Big[\frac{\sqrt{\tau\mathrm{max}}}{2}\Big]\right)\right)\chi_{init}$$

How sensitive is this to τmax?

$$\mathbf{\sigma 4 = Sen[sol4,\;\; \tau max]\;//\;FullSimplify}$$

$$\frac{1}{4}\left(2+\tau\mathrm{max}-\frac{4}{2+e^{\tau\mathrm{max}/4}\sqrt{\pi}\,\sqrt{\tau\mathrm{max}}\left(1+\mathrm{Erf}\Big[\frac{\sqrt{\tau\mathrm{max}}}{2}\Big]\right)}\right)$$

$$\mathbf{ParametricPlot}\Big[\Big\{\frac{\mathbf{sol4}}{\chi_{init}},\;\sigma 4\Big\},\;\{\tau\mathrm{max},\;0,\;20\},\;\mathrm{AspectRatio}\to 0.6,$$

```
AxesLabel → {"amplification factor", "sensitivity to τmax"},
PlotLabel → Style["Figure S4", Bold, Large]]
```

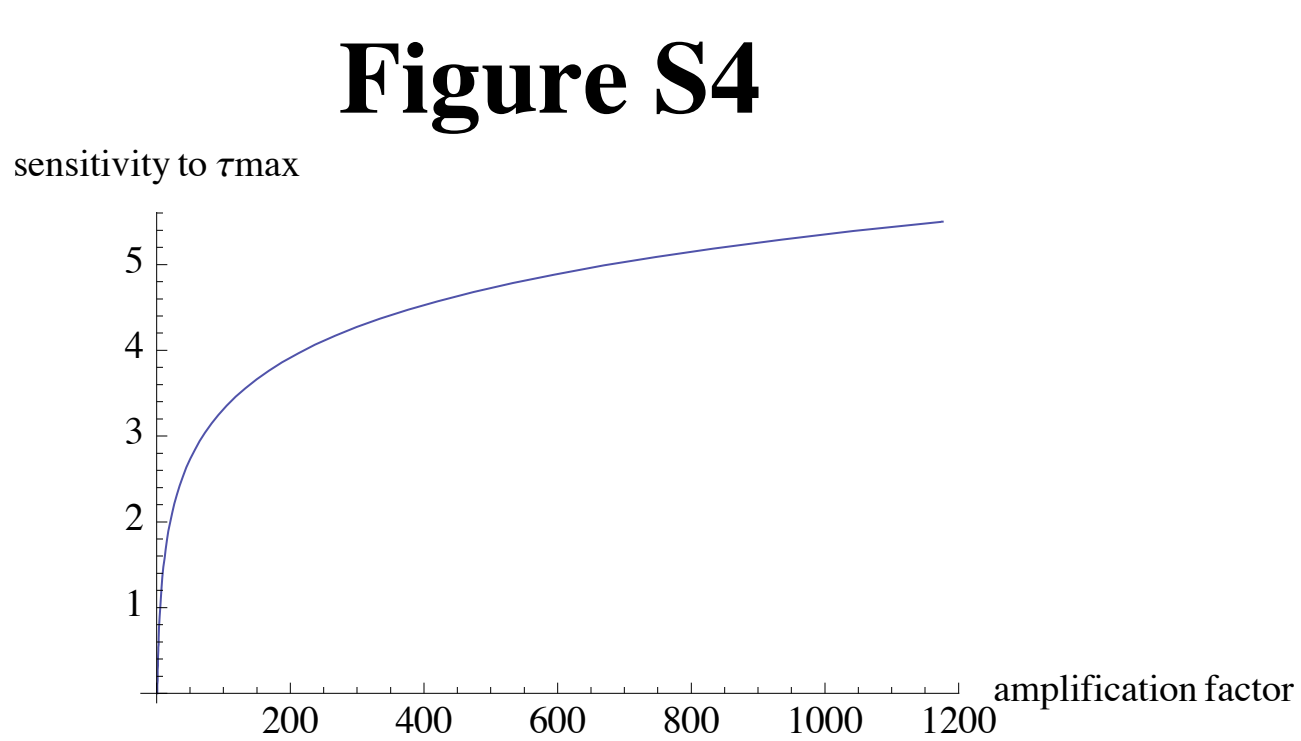

So we see that for an amplification factor of 1000, the sensitivity to the rate of decline of $p_0$is about 5, which is still quite high.

Alternatively, we may look at the special case when p goes from pmax to zero, then the expression for final output is

```
sol5 = sol3 /. pmin → 0 // FullSimplify
```

$$\frac{1}{2}\left(2+\frac{e^{\frac{1}{4}\left(-4+\frac{1}{\text{pmax}}+4\,\text{pmax}\right)\tau\text{max}}\sqrt{\pi}\;\tau\text{max}\,\text{Erfc}\left[\frac{(1-2\,\text{pmax})\,\tau\text{max}}{2\sqrt{\text{pmax}\,\tau\text{max}}}\right]}{\sqrt{\text{pmax}\,\tau\text{max}}}\right)\chi_{\text{init}}$$

```
σ5 = Sen[sol5, pmax] // FullSimplify
```

$$\left(2\,\text{pmax}\,(1+2\,\text{pmax})\,\tau\text{max}+e^{\frac{1}{4}\left(-4+\frac{1}{\text{pmax}}+4\,\text{pmax}\right)\tau\text{max}}\sqrt{\pi}\sqrt{\text{pmax}\,\tau\text{max}}\left(-2\,\text{pmax}-\tau\text{max}+4\,\text{pmax}^2\,\tau\text{max}\right)\text{Erfc}\left[\frac{(1-2\,\text{pmax})\,\tau\text{max}}{2\sqrt{\text{pmax}\,\tau\text{max}}}\right]\right)\Big/\left(4\,\text{pmax}\left(2\,\text{pmax}+e^{\frac{1}{4}\left(-4+\frac{1}{\text{pmax}}+4\,\text{pmax}\right)\tau\text{max}}\sqrt{\pi}\sqrt{\text{pmax}\,\tau\text{max}}\,\text{Erfc}\left[\frac{(1-2\,\text{pmax})\,\tau\text{max}}{2\sqrt{\text{pmax}\,\tau\text{max}}}\right]\right)\right)$$

Let us pick a variety of different τmax values, and and examine the curve relating amplification factor to sensitivity.

```
ParametricPlot[{{sol5/χinit, σ5} /. τmax → 19,
  {sol5/χinit, σ5} /. τmax → 30, {sol5/χinit, σ5} /. τmax → 40, {sol5/χinit, σ5} /. τmax → 50},
 {pmax, 0, 1}, AspectRatio → 0.8, PlotStyle → {Red, Green, Blue, Black},
 AxesLabel → {"amplification factor", "sensitivity to pmax"},
 PlotLabel → Style["Figure S5", Bold, Large]]
```

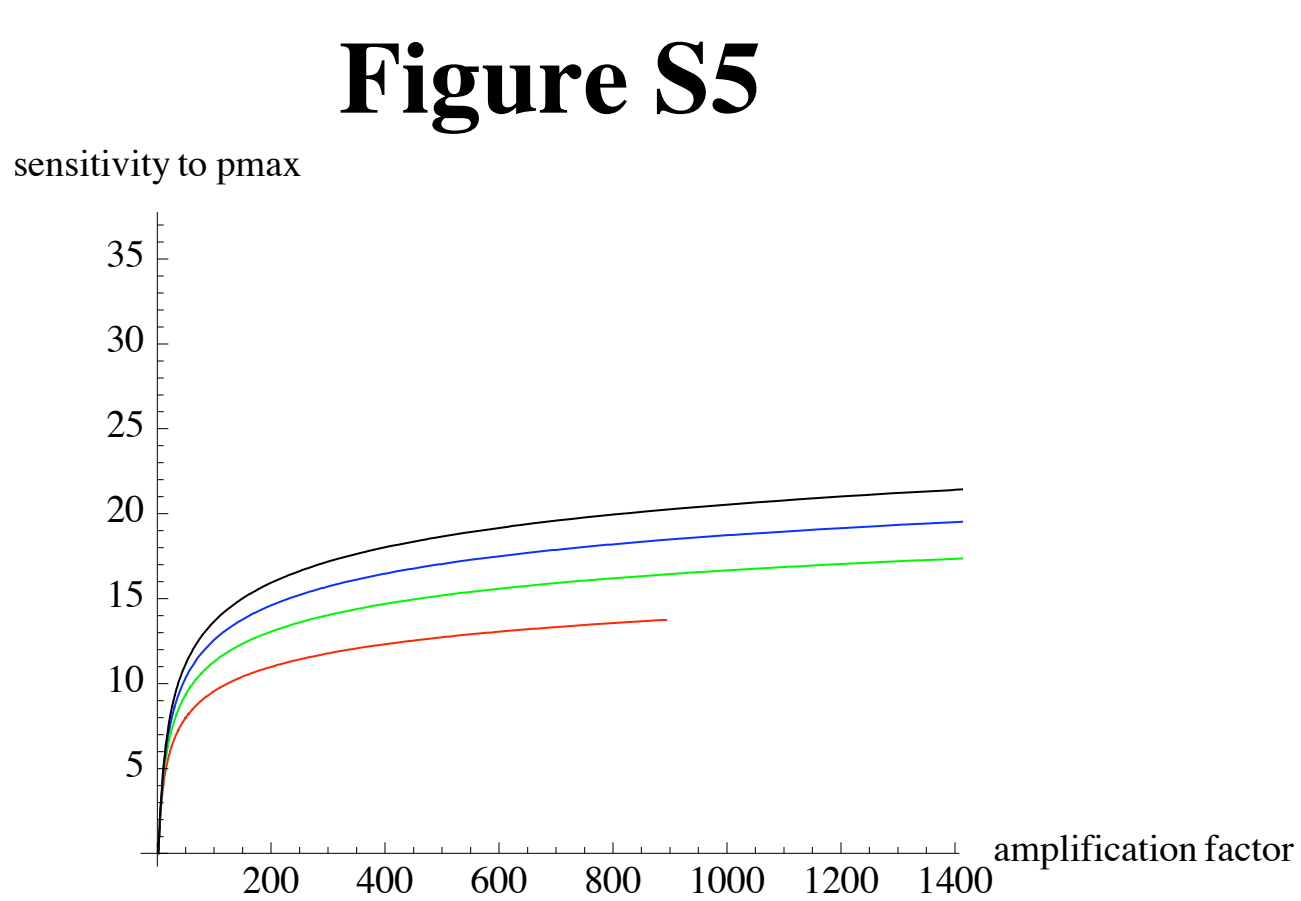

Here we see that, to get an amplification factor of 1000, sensitivity to pmax has to be at least 10, no matter the choice of τmax.

6. Final - state solutions in the presence of feedback (Fig. S6-S11)

Now let's put in feedback of the same sort we utilized in modeling steady state behaviors. We notice right away that, since it was possible to scale the units of time to v_0, and since we are only interested in the limiting behavior of the output at infinite time, then feedback onto v_0 can have no effect (i.e. the influence of v_0 on the output is only on the time scale at which output develops, but it cannot change the final value). Thus, we only consider feedback on p_0.

$$\textbf{system4} = \textbf{system2} \;/.\; p_0 \to \frac{p_0}{1 + g\,\chi_1[\tau]}$$

$$\left\{\chi_0[\tau]\left(-1 + \frac{2\,p_0}{1 + g\,\chi_1[\tau]}\right) == \chi_0'[\tau],\; 2\,\chi_0[\tau]\left(-1 + \frac{p_0}{1 + g\,\chi_1[\tau]}\right) + \chi_1'[\tau] == 0,\; \chi_{\text{init}} == \chi_0[0],\; \chi_1[0] == 0\right\}$$

Mathematica cannot solve this system of differential equations directly. However, we can determine its long-term behavior as follows: Since the first equation gives us $\frac{dx_0}{d\tau}$and the second one gives us $\frac{dx_1}{d\tau}$, then we may divide to get $\frac{dx_0}{d\chi_1}$.

$$\chi_0'[\chi_1] == \frac{-1 + \frac{2\,p_0}{1+g\,\chi_1}}{-2\left(-1 + \frac{p_0}{1+g\,\chi_1}\right)};$$

This equation can be solved. As for boundary conditions, we know that when $\chi_0 = \chi_{\text{init}}$, χ_1=0. For reasons that are not important, *Mathematica* doesn't like having subscripts in the argument for $\chi_0[\chi_1]$, so we will replace $\chi_1$with y wherever we see it, and then substitute χ_1 back in after solving.

$$\text{DSolve}\left[\left\{\chi_0'[y] == \frac{-1 + \frac{2\,p_0}{1+g\,y}}{-2\left(-1 + \frac{p_0}{1+g\,y}\right)},\; \chi_0[0] == \chi_{\text{init}}\right\},\; \chi_0[y],\; y\right] \;/.\; y \to \chi_1 \;//\; \text{Simplify}$$

$$\left\{\left\{\chi_0[\chi_1] \to \frac{(-\text{Log}[1 - p_0] + \text{Log}[1 - p_0 + g\,\chi_1])\,p_0 - g\,(\chi_1 - 2\,\chi_{\text{init}})}{2\,g}\right\}\right\}$$

As g ≠0, we may write this more compactly as

$$\chi_0 == \chi_{\text{init}} - \frac{\chi_1}{2} + \frac{p_0\,\text{Log}\left[1 + \frac{g\,\chi_1}{1-p_0}\right]}{2\,g}$$

Now in the limit of infinite time, we know from inspection of the initial system of equations that, at τ=∞, and only then, χ_0 goes to zero, and $\chi_1$goes to its final value, which we may call $\chi_{1\,\text{final}}$. We can therefore conclude:

$$0 == 2\,g\,\chi_{\text{init}} - g\,\chi_{1\,\text{final}} + p_0\,\text{Log}\left[1 + \frac{g\,\chi_{1\,\text{final}}}{1 - p_0}\right];$$

This gives us an implicit relationship between a and χ_{init}. To see this graphically, we first non-dimensionalize the cell number to g. Thus set g $\chi_{\text{init}} = \varphi$, and g $\chi_{1\,\text{final}} = \psi$. Then we have

$$0 == 2\,\varphi - \psi + p_0\,\text{Log}\left[1 + \frac{\psi}{1 - p_0}\right];$$

Below we plot ψ vs. φ for a variety of values of p_0,

```
ContourPlot[Evaluate[Table[ 2 φ - ψ + p0 Log[1 + ψ/(1 - p0)] == 0 , {p0, 0.1, 0.9, 0.1}]],
 {φ, 0, 0.1}, {ψ, 0, 4}, AxesLabel → {"g * initconds", "final state"},
 PlotLabel → Style["Figure S6", Bold, Large]]
```

Figure S6

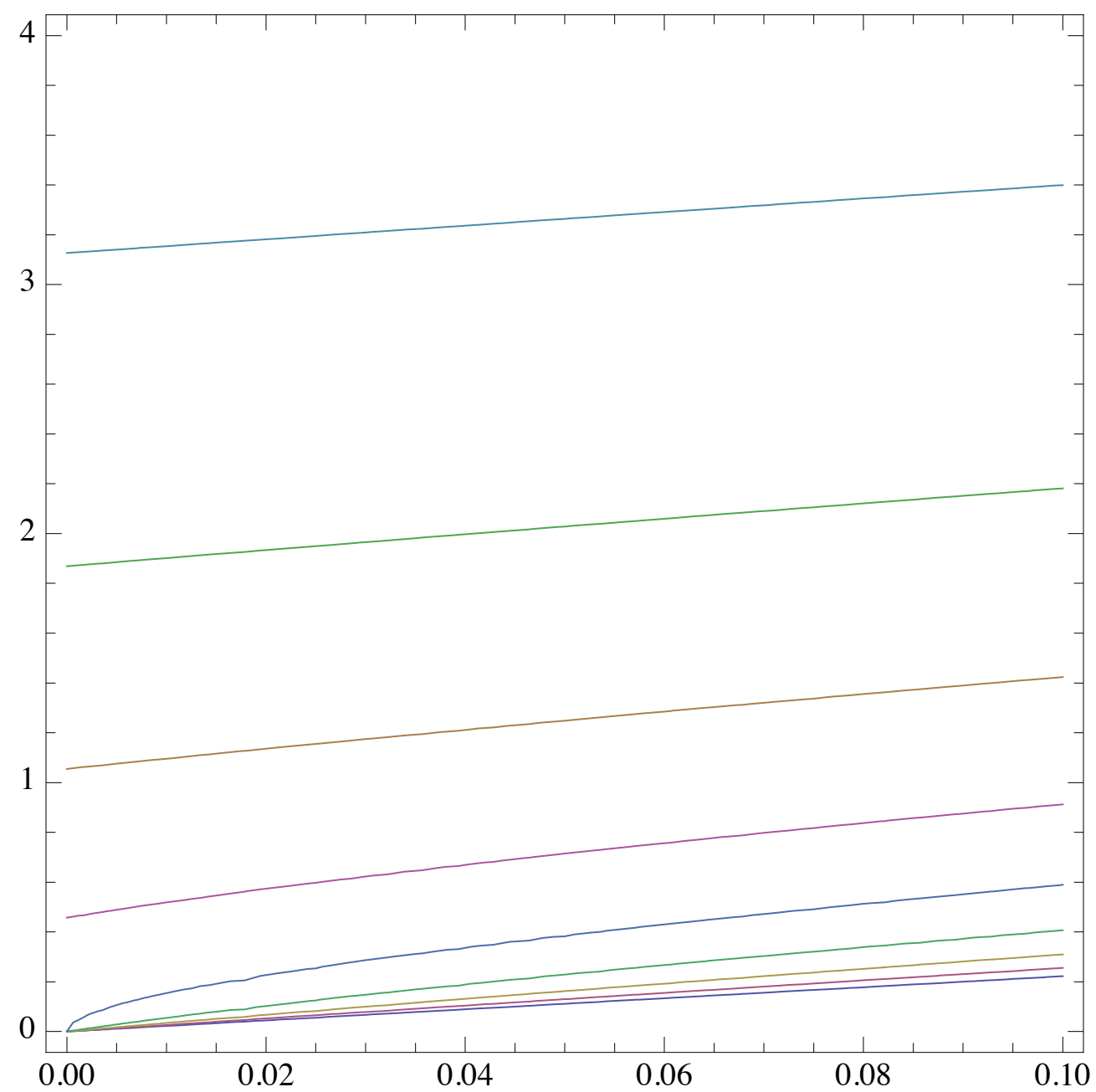

We may use log-log plotting to better explore the relationship over greater ranges, and to visualize sensitivity better

```
ContourPlot[
 Evaluate[Table[ 2 × 10^logφ - 10^logψ + p0 Log[1 + 10^logψ/(1 - p0)] == 0 , {p0, 0.1, 0.9, 0.1}]],
 {logφ, -4, 0}, {logψ, -2, 2}, AxesLabel → {"log10(g * initconds)", "log10final state"},
 PlotLabel → Style["Figure S7", Bold, Large]]
```

Figure S7

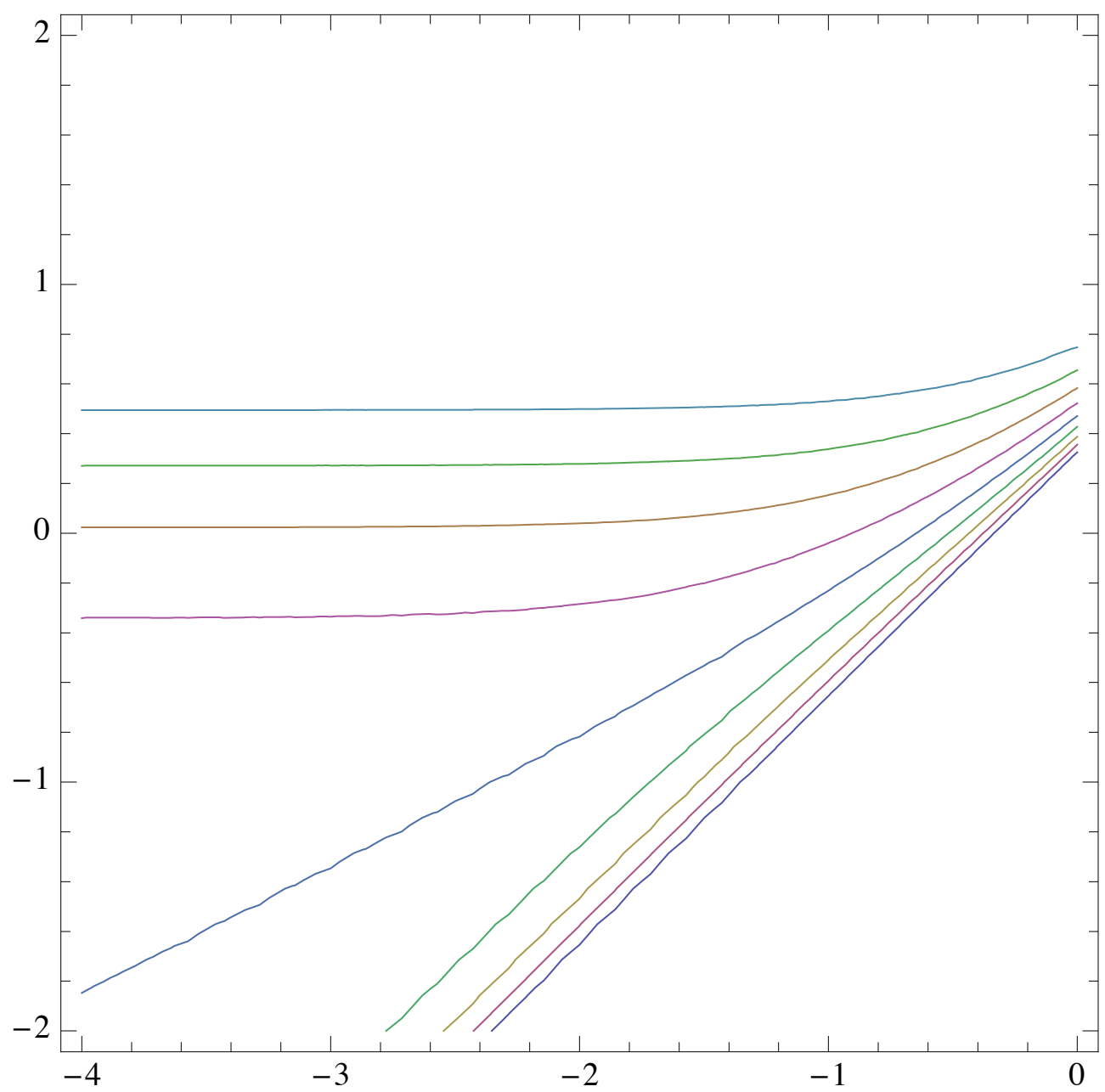

Here we see that the relationship between ψ and φ is a linear one, except when p is large and φ is small, in which case sensitivity drops very low. Are these values achievable for desirable values of the amplification factor (e.g. 1000)? We may go back to our equation and introduce the amplification factor a = $\chi_{1\,\mathrm{final}}/\chi_{\mathrm{init}}$ to get

$$0 == (2 - a)\,\varphi + p_0\,\mathrm{Log}\left[1 + \frac{a\,\varphi}{1 - p_0}\right];$$

Here we plot a vs .φ for a variety of values of p_0. Again the curves with the highest p_0 are the ones at the top.

```
ContourPlot[Evaluate[Table[ (2 - 10^loga) 10^logφ + p₀ Log[1 + (10^loga × 10^logφ)/(1 - p₀)] == 0 ,
    {p₀, 0.1, 0.9, 0.1}]], {logφ, -4, 0}, {loga, 0, 4},
  AxesLabel → {"log₁₀(g * initconds)", "log₁₀amplification factor"},
  PlotLabel → Style["Figure S8", Bold, Large]]
```

Figure S8

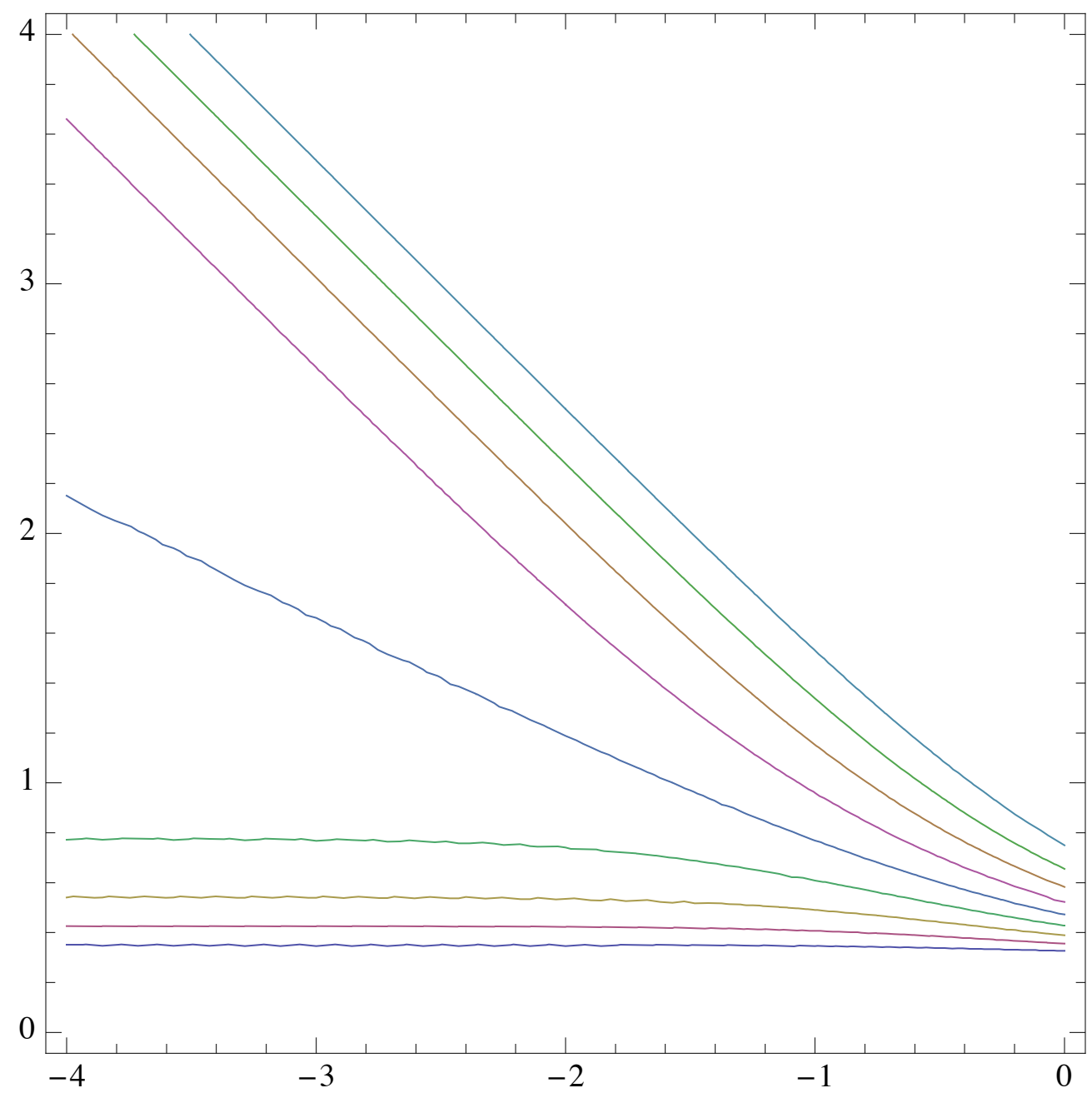

Here we see that high amplification factors occur for low φ (less than 10^{-2}) and high p_0 (>0.5). These are the same conditions that lead ψ to be insensitive to φ. Thus, we see that conditions where output is insensitive to χ_{init} are precisely those where amplification is high.

What about the sensitivity of the output to p_0?

```
ContourPlot[
 Evaluate[Table[ 2 φ - 10^logψ + 10^logp0 Log[1 + 10^logψ/(1 - 10^logp0)] == 0 , {φ, 0, 0.01, 0.001}]],
 {logp0, -1, 0}, {logψ, 0, 1}, MaxRecursion → 4,
 AxesLabel → {"log10p0", "log10final state"}, PlotLabel → Style["Figure S9", Bold, Large]]
```

Figure S9

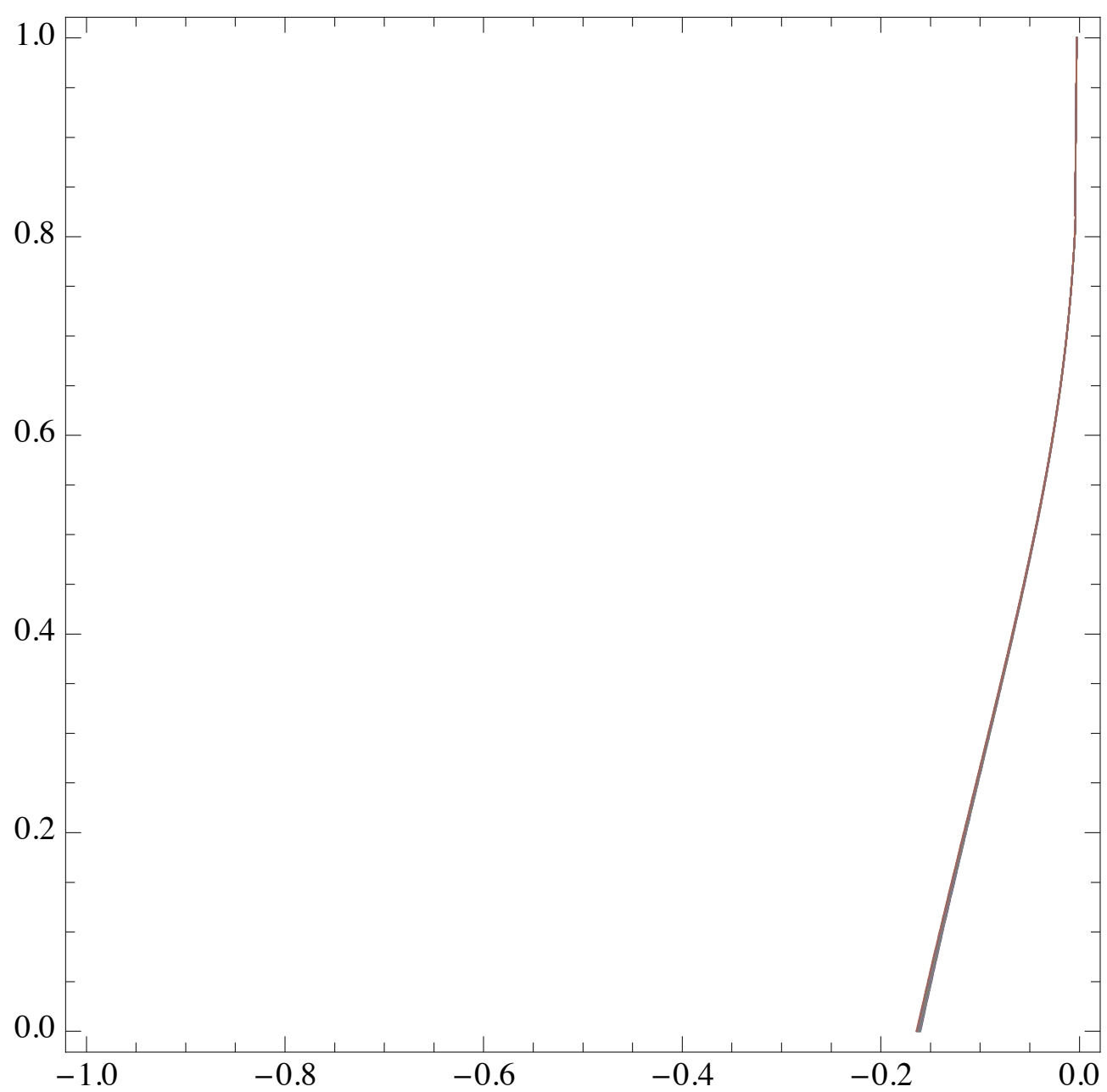

Let's specifically consider the case when the amplification factor =1000. Then $\psi/\varphi = 1000$, so we may subsitute $\psi/1000$ for φ

```
0 ==  2 φ - ψ + p0 Log[1 + ψ/(1 - p0)] /. φ → ψ / 1000
```

$$0 == -\frac{499\,\psi}{500} + \text{Log}\left[1 + \frac{\psi}{1 - p_0}\right] p_0$$

```
ContourPlot[0 == -(499 × 10^logψ)/500 + Log[1 + 10^logψ/(1 - 10^logp₀)] 10^logp₀, {logp₀, -2, 0}, {logψ, -1, 1},
 AxesLabel → {"log₁₀p₀", "log₁₀final state"}, PlotLabel → Style["Figure S10", Bold, Large]]
```

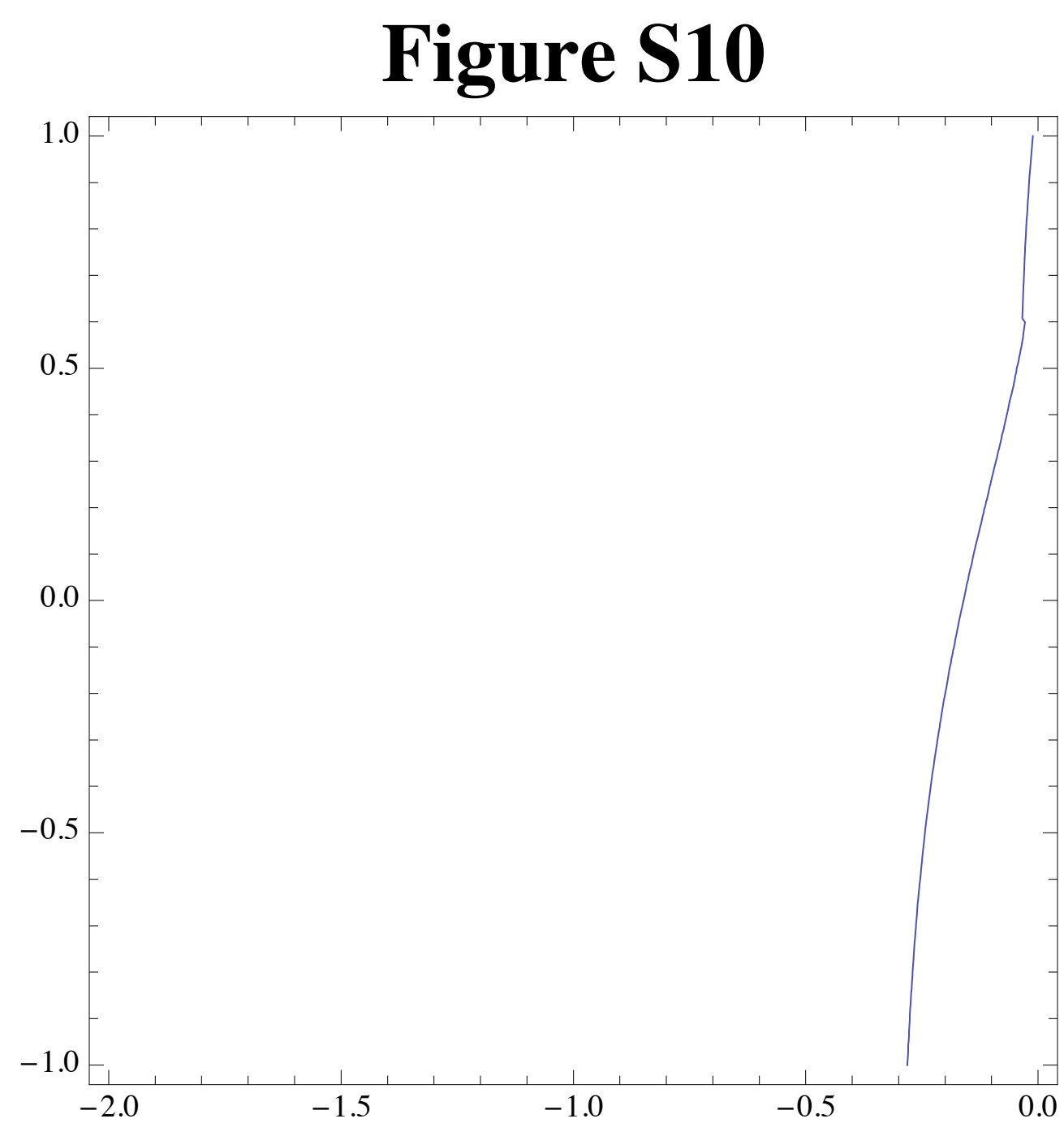

It appears there is an "optimal" zone of lowest possible sensitivity to p, which occurs for p that are close to but not too close to 1. Let's focus in a bit: We'll draw dashed lines for slopes of 4 and 5. From them we can see that when p_0 is around$10^{-0.1}$ to $10^{-0.025}$ (i.e. 0.8 to 0.95), we get a slope of the log-log plot that is somewhere between 4 and 5.

```
plot1 = ContourPlot[0 == -(499 × 10^logψ)/500 + Log[1 + (10^logψ)/(1 - 10^logp0)] 10^logp0,
   {logp0, -0.3, 0}, {logψ, -0.5, 1}, AxesLabel → {"log10p0", "log10final state"},
   PlotLabel → Style["Figure S11", Bold, Large], ContourStyle → Thick, MaxRecursion → 4];
plot2 = ContourPlot[Evaluate[Table[logψ == 0.70 + i logp0, {i, 4, 5}]],
   {logp0, -0.3, 0}, {logψ, -0.5, 1}, ContourStyle → Dashing[Large]];
Show[
 plot1,
 plot2]
```

Figure S11

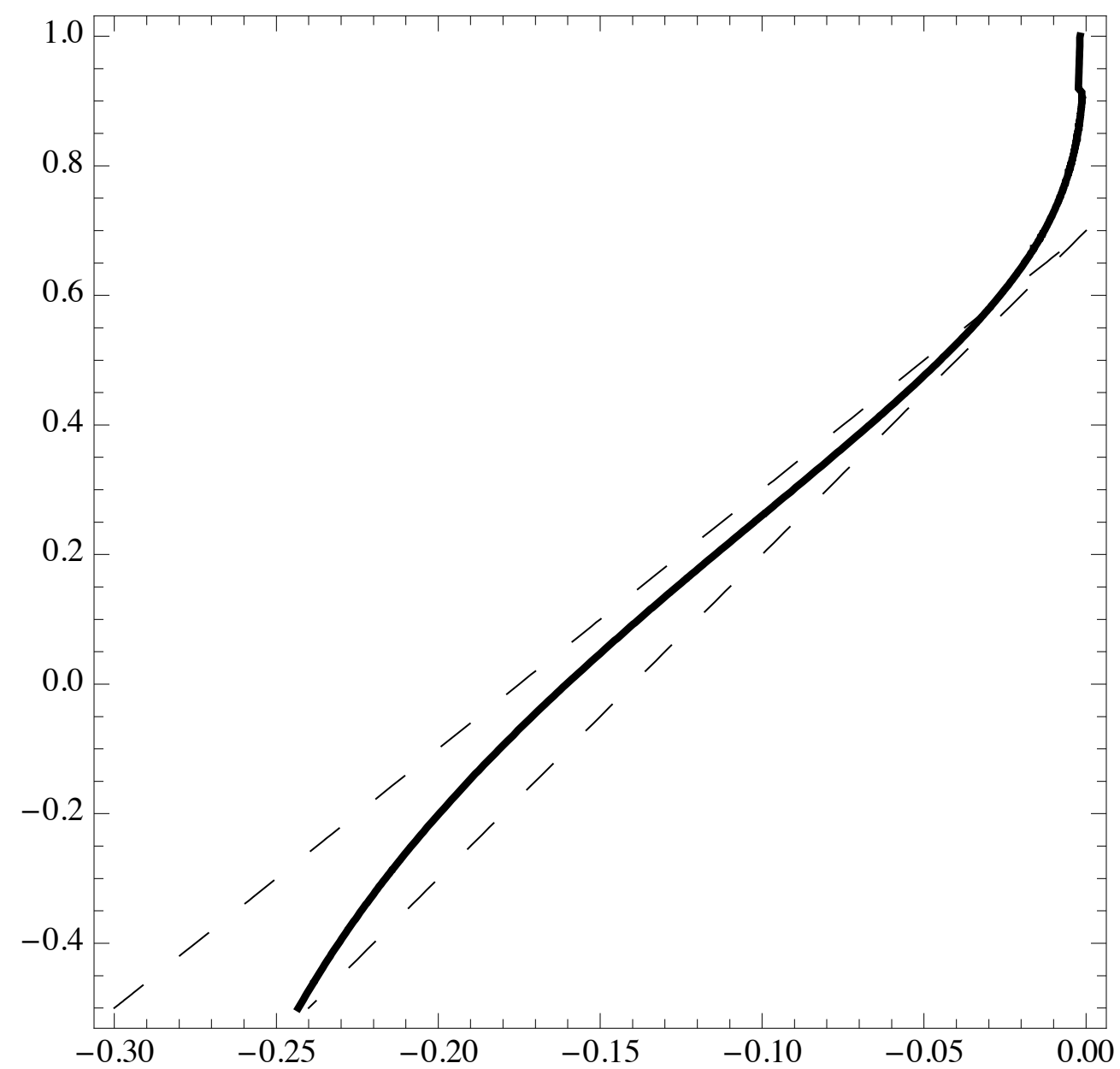

Thus, in conclusion, it is possible to to achieve a 1000-fold expansion, an extremely low sensitivity to χ_{init} and a sensitivity to $p_0$that is <5.

It is straightfoward to show that the same conclusions are reached if the expansion factor is even larger. Indeed, this is a limiting case.

7. Time-dependent solutions (Fig. S12-S13)

Here we start with the general model of section 1 for a three stage lineage, introduce feedback onto p_0, v_0, p_1 and v_1, as already described in sections 4 and 5. In addition, we nondimensionalize as in section 5 by defining a time scale $\tau = t\,v_0$. Also, as in section 4, we define $\xi = v_0 / v_1$ and $\delta = d / v_1$. (finally, because of some issues with *Mathematica* options we will write p0 instead of p_0 and p1 instead of p_1. This gives the following system of ODEs.

$$\text{system} = \left\{\chi_0'[\tau] == \frac{\xi\,\chi_0[\tau]\left(-1 + \frac{2\,\text{p0}}{1 + j\,\chi_2[\tau]}\right)}{1 + k\,\chi_2[\tau]},\right.$$

$$\left.\chi_1'[\tau] == \frac{\chi_1[\tau]\left(-1 + \frac{2\,\text{p1}}{1 + g\,\chi_2[\tau]}\right)}{1 + h\,\chi_2[\tau]} + \frac{2\,\xi\,\chi_0[\tau]\left(1 - \frac{\text{p0}}{1 + j\,\chi_2[\tau]}\right)}{1 + k\,\chi_2[\tau]},\ \chi_2'[\tau] == -\delta\,\chi_2[\tau] + \frac{2\,\chi_1[\tau]\left(1 - \frac{\text{p1}}{1 + g\,\chi_2[\tau]}\right)}{1 + h\,\chi_2[\tau]}\right\};$$

As already described in section 4, there are two non-trivial steady state solutions for this system, which we will call solset and altsolset.

```
solset = {χss0 → -((-1 + 2 p0) (j + k (-1 + 2 p0)) (j + g (-1 + 2 p0) - 2 j p1) δ)/(2 j^2 (g - 2 g p0 + j (-1 + p1)) ζ),
   χss1 → -((-1 + 2 p0) (j + g (-1 + 2 p0)) (j + h (-1 + 2 p0)) δ)/(2 j^2 (g - 2 g p0 + j (-1 + p1))), χss2 → (-1 + 2 p0)/j};
altsolset = {χss0 → 0, χss1 → ((-1 + 2 p1) (g + h (-1 + 2 p1)) δ)/g^2, χss2 → (-1 + 2 p1)/g};
```

We may explore the time dependent behavior of the system for various choices of initial condition: For example, here's a starting case in which the initial conditions have χ_0 and χ_1 starting at their steady state values, but $\chi_2$at zero, mimicking regeneration in the olfactory epithelium following an acute loss of ORNs.

```
With[{s1 = system}, Manipulate[
  Plot[Evaluate[{χ0[τ], χ1[τ], χ2[τ]} /.
      Flatten[NDSolve[Join[s1, {χ0[0] == 0.367059, χ1[0] == 1.08706, χ2[0] == 0}],
         {χ0[τ], χ1[τ], χ2[τ]}, {τ, 0, 1.2 Log[5]/δ}]]],
   {τ, 0, 1.2 Log[5]/δ}, PlotRange → All, PlotStyle → {Green, Red, Blue}],
  Style["Figure S12", Bold, Large], Item["Steady state solution is"],
  Dynamic[Evaluate[If[(-1 + 2 p1)/g > (-1 + 2 p0)/j, Evaluate[altsolset], Evaluate[solset]]]],
  {{p0, 0.51}, 0.001, 0.999}, {{p1, 0.55}, 0.001, 0.999},
  {{δ, 0.012}, 0.002, 0.2}, {{ζ, 1.5}, 0.1, 10}, {{g, 0.04}, 0.001, 5},
  {{h, 1}, 0, 10}, {{j, 0.002}, 0.001, 5}, {{k, 2.5}, 0, 200}]]
```

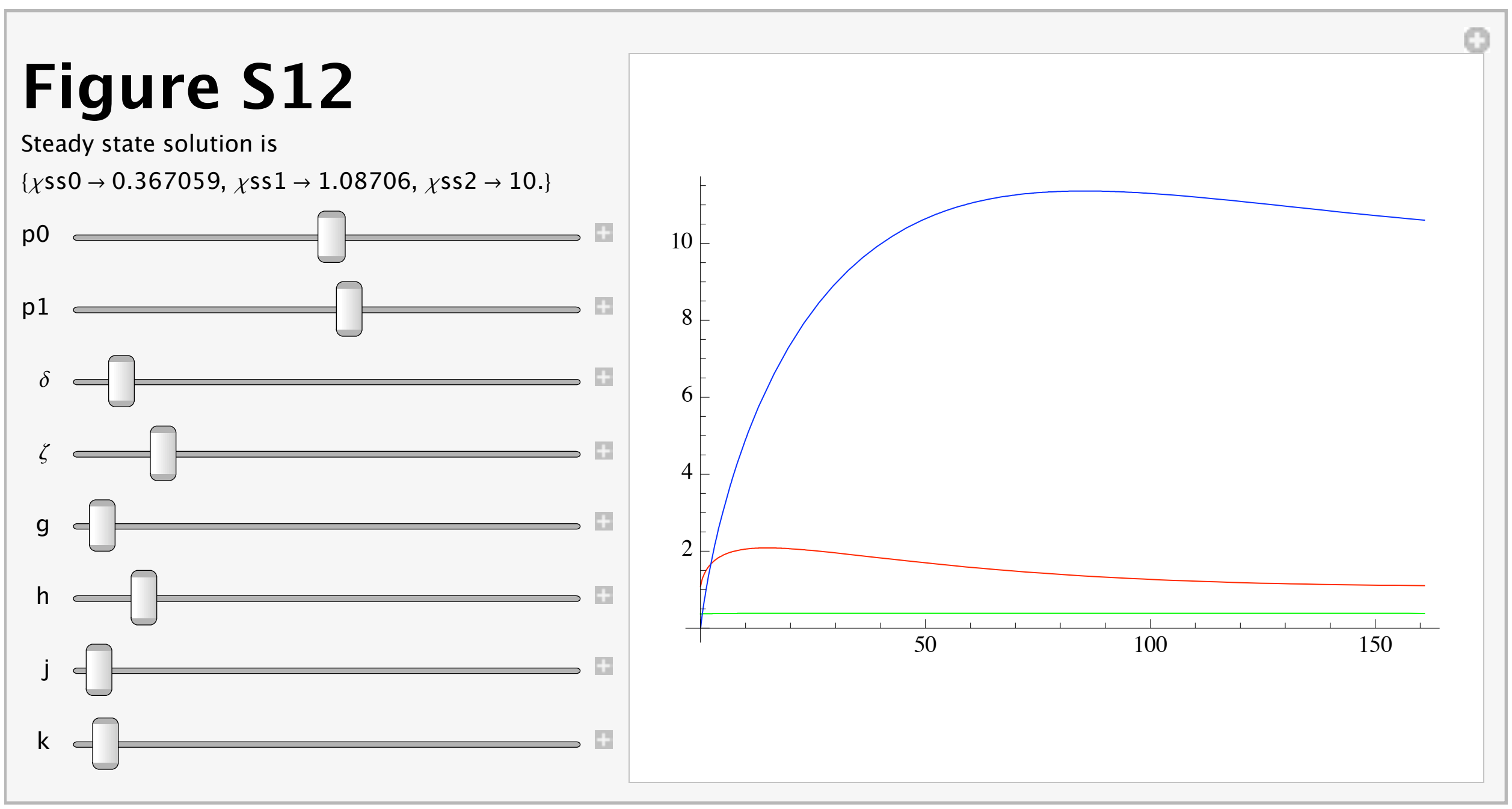

Below is a case in which the steady state solution tells us that cell type "0" will eventually become extinguished. However, because the feedback on $v_0$by cell type "2" is so large, when numbers of cell type "2" are significant, cell type "0" goes through cell cycles at an extremely slow rate. As a result, extinction is forestalled for many hundreds of cell cycles (potentially for the lifetime of the organism)

```
With[{s1 = system}, Manipulate[
  Plot[Evaluate[{χ0[τ], χ1[τ], χ2[τ]} /. Flatten[NDSolve[
      Join[s1, {χ0[0] == 0.2, χ1[0] == 0, χ2[0] == 0}], {χ0[τ], χ1[τ], χ2[τ]}, {τ, 0, 50 Log[5]/δ}]]],
   {τ, 0, 50 Log[5]/δ}, PlotRange → All, PlotStyle → {Green, Red, Blue}],
  Style["Figure S13", Bold, Large], Item["Steady state solution is"],
  Dynamic[Evaluate[If[(-1 + 2 p1)/g > (-1 + 2 p0)/j, Evaluate[altsolset], Evaluate[solset]]]],
  {{p0, 0.95}, 0.001, 0.999}, {{p1, 0.85}, 0.001, 0.999},
  {{δ, 0.2}, 0.002, 0.2}, {{ζ, 1}, 0.1, 10}, {{g, 0.16}, 0.001, 5},
  {{h, 0.1}, 0, 10}, {{j, 0.3}, 0.001, 5}, {{k, 40}, 0, 200}]]
```

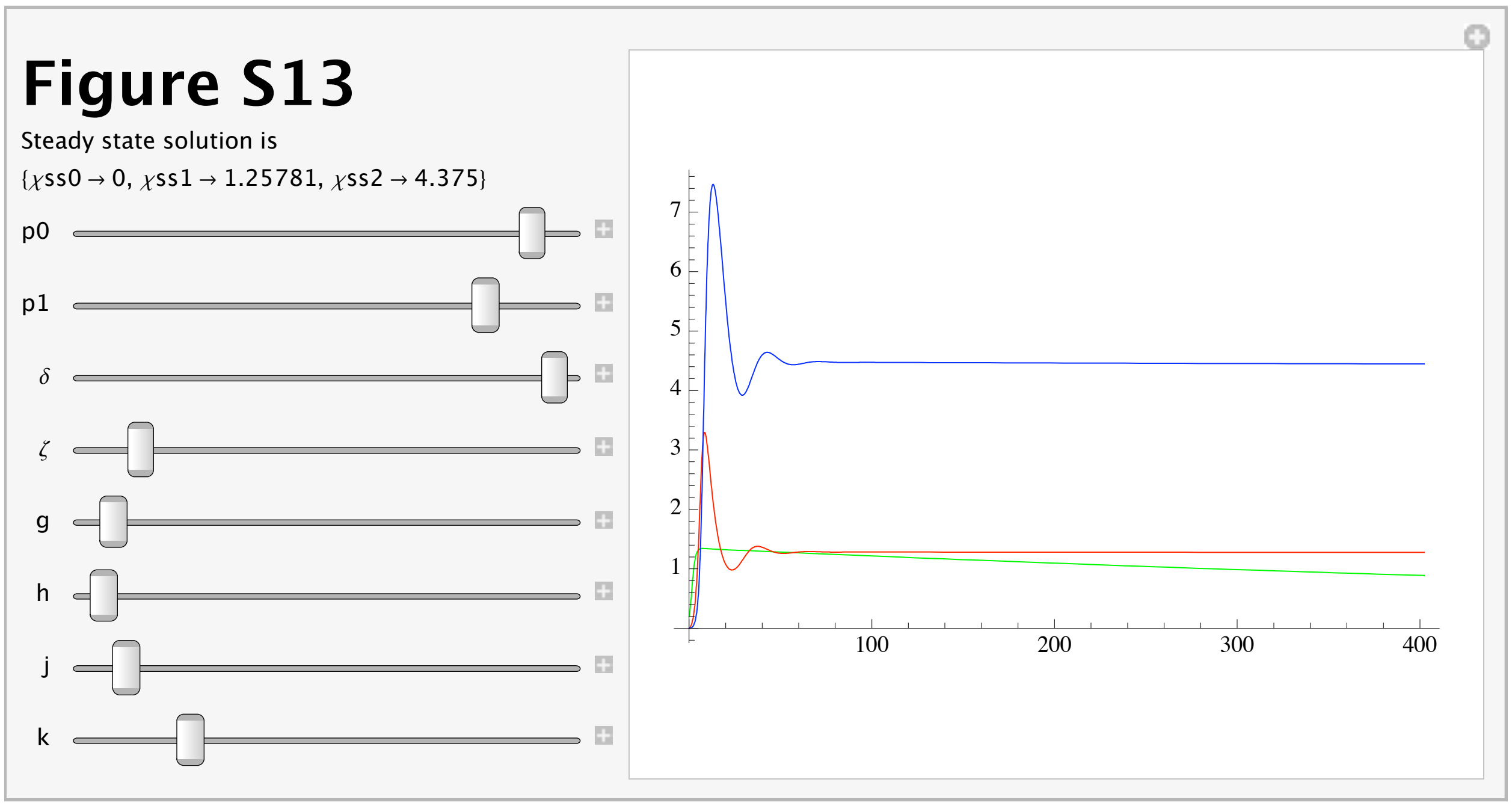

If a case like the one above were to occur in nature, what would an experimental biologist observe? The system would appear to contain a very slowly cycling stem cell (it might even be called "quiescent" and would certainly be found to be "label-retaining", i.e. after one round of division it would be a very long time before such a cell underwent a subsequent round), which only becomes highly proliferative in response to tissue injury. The experimentalist might also observe that the numbers of such stem cells gradually decline over the lifetime of the organism. All of these observations are ones that have frequently been made for tissue stem cells in various contexts. Here they arise simply as a result of feedback interactions. The cell that displays them has no innate programming to do so. In the absence of feedback, the proliferative and differentiative behaviors of cell type "0" would, in fact, be very similar to those of cell type "1".

8. Parameter space exploration—methods

To explore the dynamic behavior of different feedback models, the following steps are followed

1. The names of the parameters of the system are given in a list named "params"

2. If the system has no feedback on $p_0$then the only non-trivial steady state is one in which $p_0 = 0.5$, and χ_0 is arbitrary. In this case, the variables χ_1 and χ_2 are normalized to $\chi_0$and renamed c_1 and c_2 respectively. In addition, the parameters g and h are multiplied by $\chi_0$to give the new parameters γ and η, respectively. By all this normalization, $\chi_0$may be eliminated entirely from the system of equations.

3. If the steady state solution can be solved for directly, it is named "solset". If the variables were $\chi_0[\tau]$, $\chi_1[\tau]$ and $\chi_2[\tau]$, their steady state versions are named χss0, χss1 and χss2. If the variables were $c_1[\tau]$ and $c_2[\tau]$their steady state versions are named css1 and css2. [N.B. if there is more than one steady state, only one is chosen here; parameter values that are inconsistent with positive solutions for that steady state will get identified during the run and saved in a separate file; these may be re-run later, using a different one of the steady state solutions in the code]

If it cannot be solved directly in any reasonably compact form, the system of equations that determines the steady state is named "sssystem" and left unsolved.

4. If the steady state solution was solved for directly, the set of ODEs representing the model is transformed with new variables z_0, $z_1$and z_2 representing χ_1, χ_2 and χ_3, or $c_1$and c_2, normalized to their steady state values. This is given the name "system". If the steady state could not be solved for directly, the set of ODEs is left in its original form and given the name "tempsystem"

5. Definitions and parameter ranges are then entered as below: Initconds refer to intial conditions where different fractions of different cell types are eliminated. Endpoints refer to the expected times to reach 80% of steady state for systems with the corresponding initial conditions, in the absence of any feedback regulation.

a. If the system has feedback on $p_0$then the following is used:

```
vars = {z0[τ], z1[τ], z2[τ]};
initconds[1] = {z0[0] == 1, z1[0] == 1, z2[0] == 0};
initconds[2] = {z0[0] == 1, z1[0] == 0, z2[0] == 0};
initconds[3] = {z0[0] == 1, z1[0] == 1, z2[0] == 0.25};
initconds[4] = {z0[0] == 0.5, z1[0] == 0, z2[0] == 0};
endpoint[1] = 1.2 Log[5] / δ;
endpoint[2] = 1.2 Log[5] / δ;
endpoint[3] = 1.2 (Log[5] - Log[1 / (1 - initconds[3][[3, 2]])]) / δ;
endpoint[4] = 1 / ξ + 1.2 Log[5] / δ;
p0range = {0.5, 1};
p1range = {0, 1};
Logδrange = {-2.5, -0.5};
Logξrange = {-1, 1};
Loggrange = {-2, 1};
Loghrange = {-2, 1};
Logjrange = {-2, 1};
Logkrange = {-2, 1};
wholeset = Join[params,
  {f[1], f[2], f[3], f[4], css0, css1, css2, t[1], t[2], t[3], t[4], senx, send}];
Valu[list_, symbol_] := First[Part[list, Position[wholeset, symbol][[1]]]];
```

The penultimate statement defines a list that joins the parameters to a variety of other things that will be defined later. The last statement defines a function that picks out from any list the element that is at the same position as the named symbol is in wholeset.

b. If the system has no feedback on $p_0$then the following is used instead:

```
vars = {z1[τ], z2[τ]};
initconds[1] = {z1[0] == 1, z2[0] == 0};
initconds[2] = {z1[0] == 0, z2[0] == 0};
initconds[3] = {z1[0] == 1, z2[0] == 0.25};
endpoint[1] = 1.2 Log[5] / δ;
endpoint[2] = 1.2 Log[5] / δ;
endpoint[3] = 1.2 (Log[5] - Log[1 / (1 - initconds[3][[2, 2]])]) / δ;
p1range = {0, 1};
Logδrange = {-2.5, -0.5};
Logξrange = {-1, 1};
Logγrange = {-5, 1};
Logηrange = {-2, 2};
wholeset = Join[params, {f[1], f[2], f[3], css1, css2, t[1], t[2], t[3], sen}];
Valu[list_, symbol_] := First[Part[list, Position[wholeset, symbol][[1]]]];
```

6. If the Hill functions in the ODE system have Hill coefficients n, the values of these are next specified. e.g. {ng, nh, nj, nk} = {2, 2, 2, 2};

7. Next, calculated expression for the values of sensitivity to χ_0 and sensitivity to δ are are given the names σx and $\sigma\delta$, respectively. These may be defined in terms of the parameters or in terms of steady state values of the variables, as these will be calculated anyway.

If there is feedback on p_0, the following code is then run; it is a Do-loop that runs for 20,000 times, writing a result to the end of a file each time.

```
iterations = 20 000;
Timing[OpenAppend["filename", FormatType → OutputForm, PageWidth → Infinity];
 OpenAppend["filenamefailed", FormatType → OutputForm, PageWidth → Infinity];
 Do[{p0, p1, δ, ξ, g, h, j, k} = {RandomReal[p0range], RandomReal[p1range],
    10^RandomReal[Logδrange], 10^RandomReal[Logξrange], 10^RandomReal[Loggrange],
    10^RandomReal[Loghrange], 10^RandomReal[Logjrange], 10^RandomReal[Logkrange]};
  cell0 = χss0 /. solset; cell1 = χss1 /. solset; cell2 = χss2 /. solset;
  If[cell0 ≤ 0 ∨ cell1 ≤ 0, Write["filenamefailed", {p0, p1, δ, ξ, g, h, j, k}],

   f[1] = Log[2] * If[ξ ≤ 1, Log[2, (2^(1/ξ) (cell0 + cell1 + cell2))/(2 cell0 + 2^(1/ξ) cell1)], n /. FindRoot[
        2^(1+ξ (n-1)) cell0 + 2^n cell1 == cell0 + cell1 + cell2, {n, 0, 20}, Method → "Brent"]];
   f[2] = Log[2] * If[ξ ≤ 1, Max[0, -1 + 1/ξ + Log[2, (cell0 + cell1 + cell2)/cell0]],
      Max[0, ((-1 + ξ) + Log[2, (cell0+cell1+cell2)/cell0])/ξ]];
   f[3] = Log[2] * If[ξ ≤ 1, Log[2, (4 cell0 + 4 cell1 + 3 cell2)/(2^(3-1/ξ) cell0 + 4 cell1)], n /. FindRoot[
        2^(1+ξ (n-1)) cell0 + 2^n cell1 == cell0 + cell1 + (3 cell2)/4, {n, 0, 20}, Method → "Brent"]];
   f[4] = f[2] + Log[2]/ξ;
   Table[plotdata[i] = InputForm[Plot[Evaluate[
         Last[vars] /. Flatten[NDSolve[Join[system, initconds[i]], vars, {τ, 0, endpoint[i]}]]],
        {τ, 0, endpoint[i]}, PlotRange → All]], {i, 1, 4}];
   Table[list[i] = Flatten[Cases[plotdata[i], Line[z_] → z, Depth[plotdata[i]]], 1],
    {i, 1, 4}];
   Table[w[i] = Length[list[i]]; While[0.8` < list[i][[w[i], 2]] < 1.2` && w[i] > 1,
     w[i] = w[i] - 1], {i, 1, 4}];
   q = wholeset /. {css0 → cell0, css1 → cell1, css2 → cell2,
      t[1] → list[1][[w[1], 1]], t[2] → list[2][[w[2], 1]],
      t[3] → list[3][[w[3], 1]], t[4] → list[4][[w[4], 1]], senx → σx, send → σδ};
   Write["filename", AccountingForm[q]]],
  {iterations}];
 Close["filename"]; Close["filenamefailed"];
 Clear[iterations, cell0, cell1, cell2, f, list, w, p0, p1, δ, ξ, g, h, j, k]]
```

Here the code is annotated:

```
iterations = 20 000;
Timing[OpenAppend["filename", FormatType → OutputForm, PageWidth → Infinity];
 OpenAppend["filenamefailed", FormatType → OutputForm, PageWidth → Infinity];
 (*these steps open the files.  Normally, one would enter a different filename for
  each model.  The file named "filenamefailed" is there to hold the parameters
  of any parameter sets that might fail to yield a positive steady state*)
 Do[{p0, p1, δ, ξ, g, h, j, k} = {RandomReal[p0range], RandomReal[p1range],
    10^RandomReal[Logδrange], 10^RandomReal[Logξrange], 10^RandomReal[Loggrange],
    10^RandomReal[Loghrange], 10^RandomReal[Logjrange], 10^RandomReal[Logkrange]};
  (*these steps choose values for the parameters at randome from the indicated ranges*)
  cell0 = χss0 /. solset; cell1 = χss1 /. solset; cell2 = χss2 /. solset;
  (*these commands calcualte the steady state values of cell types 0,
```

```
1 and 2, from the steady state solution that was provided*)
If[cell0 ≤ 0 ∨ cell1 ≤ 0, Write["filenamefailed", {p0, p1, δ, ξ, g, h, j, k}],
 (*The first part of this IF statement test whether any cell type gives steady state less
  than or equal to zero, and if so, writes the parameters to "filenamefailed",
 and moves on to a new parameter set.  Otherwise four f-values are
   calculated.  These are estimations of the fastest possible regeneration times,
 given the number of cells that need to be made--i.e. they come from models
  in which every cell divides the minimum number of times to produce
  the right total number of cells, which then, at the very end,
 differentiate.  These values were not used in any of the data plotted in the manuscript,
 but are useful for comparison*)
 f[1] = Log[2] * If[ξ ≤ 1, Log[2, (2^(1/ξ) (cell0 + cell1 + cell2))/(2 cell0 + 2^(1/ξ) cell1)], n /. FindRoot[
      2^(1+ξ(n-1)) cell0 + 2^n cell1 == cell0 + cell1 + cell2, {n, 0, 20}, Method → "Brent"]];
 f[2] = Log[2] * If[ξ ≤ 1, Max[0, -1 + 1/ξ + Log[2, (cell0 + cell1 + cell2)/cell0]],
     Max[0, ((-1 + ξ) + Log[2, (cell0+cell1+cell2)/cell0])/ξ]];
 f[3] = Log[2] * If[ξ ≤ 1, Log[2, (4 cell0 + 4 cell1 + 3 cell2)/(2^(3-1/ξ) cell0 + 4 cell1)], n /. FindRoot[
      2^(1+ξ(n-1)) cell0 + 2^n cell1 == cell0 + cell1 + (3 cell2)/4, {n, 0, 20}, Method → "Brent"]];
 f[4] = f[2] + Log[2]/ξ;
 Table[plotdata[i] = InputForm[Plot[Evaluate[
      Last[vars] /. Flatten[NDSolve[Join[system, initconds[i]], vars, {τ, 0, endpoint[i]}]]],
     {τ, 0, endpoint[i]}, PlotRange → All]], {i, 1, 4}];
 (*The above command creates a plot of the dynamic behavior of the system,
 for some initial conditions, from a time of zero to an endpoint;
 it does so for four different initial conditions and endpoints.  These plots are
  read in in InputForm, which means the points are a list, rather than a Graphic*)
 Table[list[i] = Flatten[Cases[plotdata[i], Line[z_] → z, Depth[plotdata[i]]], 1],
  {i, 1, 4}];
 (*This strips out unnecessary information from the list*)
 Table[w[i] = Length[list[i]];
  While[0.8` < list[i]〚w[i], 2〛 < 1.2` && w[i] > 1, w[i] = w[i] - 1], {i, 1, 4}];
 (*This starts at the last point in each list and moves toward zero,
 checking for the first value of the terminal cell type that lies OUTSIDE of
  the band of 20% around its steady state value.  This is given the name w[i],
 with i being an index for each of the four initial conditions.  *)
 q = wholeset /. {css0 → cell0, css1 → cell1, css2 → cell2,
    t[1] → list[1]〚w[1], 1〛, t[2] → list[2]〚w[2], 1〛,
    t[3] → list[3]〚w[3], 1〛, t[4] → list[4]〚w[4], 1〛, senx → σx, send → σδ};
 (*This creates a temporary list called q,
 which stores along with the parameter values and the f-values,
 the three steady state values, and the times t[i] at which the w[i] occur for each i*)
 Write["filename", AccountingForm[q]]
 (*This appends q to the file*)
],
{iterations}];
Close["filename"]; Close["filenamefailed"];
Clear[iterations, cell0, cell1, cell2, f, list, w, p0, p1, δ, ξ, g, h, j, k]]
```

8. In the event that the steady state system cannot be solve for directly, the code is modified as follows:

```
Timing[OpenAppend["filename", FormatType → OutputForm, PageWidth → Infinity];
 OpenAppend["filenamefailed", FormatType → OutputForm, PageWidth → Infinity];
 Do[{p0, p1, δ, ξ, g, h, j, k} = {RandomReal[p0range], RandomReal[p1range],
    10^RandomReal[Logδrange], 10^RandomReal[Logξrange], 10^RandomReal[Loggrange],
    10^RandomReal[Loghrange], 10^RandomReal[Logjrange], 10^RandomReal[Logkrange]};
  sssol = Flatten[Select[NSolve[sssystem, {χss0, χss1, χss2}],
     (Re[#[[1, 2]]] > 0 && Im[#[[1, 2]]] == 0 && Re[#[[2, 2]]] > 0 &&
        Im[#[[2, 2]]] == 0 && Re[#[[3, 2]]] > 0 && Im[#[[3, 2]]] == 0) &]];
  (*The above finds the steady state solution numerically, after the parameters
   have been selected.  Since in many cases multiple steady states exist,
  the command here discard any that have imaginary or negative-
   valued solutions for any of the cell types*)
  If[Length[sssol] == 0, Write["filenamefailed", params] ,
   cell0 = χss0 /. sssol; cell1 = χss1 /. sssol; cell2 = χss2 /. sssol;
   system = tempsystem /. {χ0[τ] → z0[τ] * cell0, χ0'[τ] → z0'[τ] * cell0, χ1[τ] → z1[τ] * cell1,
      χ1'[τ] → z1'[τ] * cell1, χ2[τ] → z2[τ] * cell2, χ2'[τ] → z2'[τ] * cell2} ;
   (*this command creates the normalized system, by using the newly calculated steady state
    values to normalize the variables.  From this point on the code proceeds as before*)
```

9. Recall that the cases "filenamefailed" do not necessarily represent cases with no steady state solution. For the systems examined here, they invariably are cases in which χ_0 goes to zero and non-zero steady states are reached for χ_1 and χ_2 (i.e. χ_0 becomes extinguished and χ_1 takes over as the stem cell).

For these cases, the parameters are subsequently read back out and used as the starting points for a similar parameter space exploration, but using a different set of steady-state equations based specifically on the steady state in which $\chi_0 \to 0$.

10. If there is no feedback on p_0 then the code is a little different and simpler (fewer initial conditions are possible, one less variable):

```
iterations = 20000;
Timing[OpenWrite["filename", FormatType → OutputForm, PageWidth → Infinity];
 Do[{p, δ, ξ, γ, η} = {RandomReal[p1range], 10^RandomReal[Logδrange],
    10^RandomReal[Logξrange], 10^RandomReal[Logγrange], 10^RandomReal[Logηrange]};
  cell1 = css1 /. solset; cell2 = css2 /. solset; f[1] = If[ξ ≤ 1, Log[2, (2^(1/ξ) (1 + cell1 + cell2))/(2 + 2^(1/ξ) cell1)],
    n /. FindRoot[2^(1+ξ(n-1)) + 2^n cell1 == 1 + cell1 + cell2, {n, 0, 20}, Method → "Brent"]];
  f[2] = If[ξ ≤ 1, Max[0, -1 + 1/ξ + Log[2, 1 + cell1 + cell2]],
    Max[0, ((-1 + ξ) + Log[2, 1 + cell1 + cell2])/ξ]];
  f[3] = If[ξ ≤ 1, Log[2, (4 + 4 cell1 + 3 cell2)/(2^(3-1/ξ) + 4 cell1)],
    n /. FindRoot[2^(1+ξ(n-1)) + 2^n cell1 == 1 + cell1 + (3 cell2)/4, {n, 0, 20}, Method → "Brent"]];
  Table[plotdata[i] = InputForm[Plot[Evaluate[Last[vars] /.
        Flatten[NDSolve[Join[system, initconds[i]], vars, {τ, 0, endpoint[i]}]]],
      {τ, 0, endpoint[i]}, PlotRange → All]], {i, 1, 3}];
  Table[list[i] = Flatten[Cases[plotdata[i], Line[z_] → z, Depth[plotdata[i]]], 1],
   {i, 1, 3}];
  Table[w[i] = Length[list[i]]; While[0.8` < list[i][[w[i], 2]] < 1.2` && w[i] > 1, w[i] = w[i] - 1],
   {i, 1, 3}];
  q = wholeset /. {css1 → cell1, css2 → cell2, t[1] → list[1][[w[1], 1]],
     t[2] → list[2][[w[2], 1]], t[3] → list[3][[w[3], 1]], sen → σx};
  Write["filename", AccountingForm[q]],
  {iterations}];
 Close["filename"];
 Clear[iterations, cell1, cell2, f, list, w, p, δ, ξ, γ, η]]
```

▫ ***"Fold Improvement in Regeneration Speed"***

For all the cases in the text in which "improvement in regeneration speed" is referred to, the values were obtained from parameter-space explorations by comparing the t[i] values (the last times at which terminal cell values are either 20% above or below steady state), with the times that would have been expected, given the same steady state cell levels, but no feedback, i.e. assuming that the rate constant for approach to steady state is δ.

Those times were:

For initial conditions #1: $\ln 5/\delta$

For initial conditions #2: $\ln 5/\delta$

For initial conditions #3: $\ln(15/4)/\delta$

For initial conditions #4: $\ln 5/\delta$

9. Parameter space exploration— supplemental results (Fig. S14-S22)

- ***Here we consider the case with feedback only on v_1. The file with the parameter space exploration of that model (see previous section) was named "2V1" and is read in and analyzed by the following code:***

```
params = {p, δ, ξ, η};
wholeset = Join[params, {f[1], f[2], f[3], css1, css2, t[1], t[2], t[3], sen}];
Valu[list_, symbol_] := First[Part[list, Position[wholeset, symbol][[1]]]];
ListPlot[Map[{(1 + Valu[#, css1])/(1 + Valu[#, css1] + Valu[#, css2]), Log[5]/(Valu[#, δ] Valu[#, t[1]])} &,
   ToExpression[Import["2V1", "List"]]], PlotRange → All, GridLines → Automatic,
  AxesLabel → {"progenitor load", "improvement in regeneration speed"},
  ImageSize → 500, PlotLabel → Style["Figure S14", Bold, Large]]
```

Figure S14

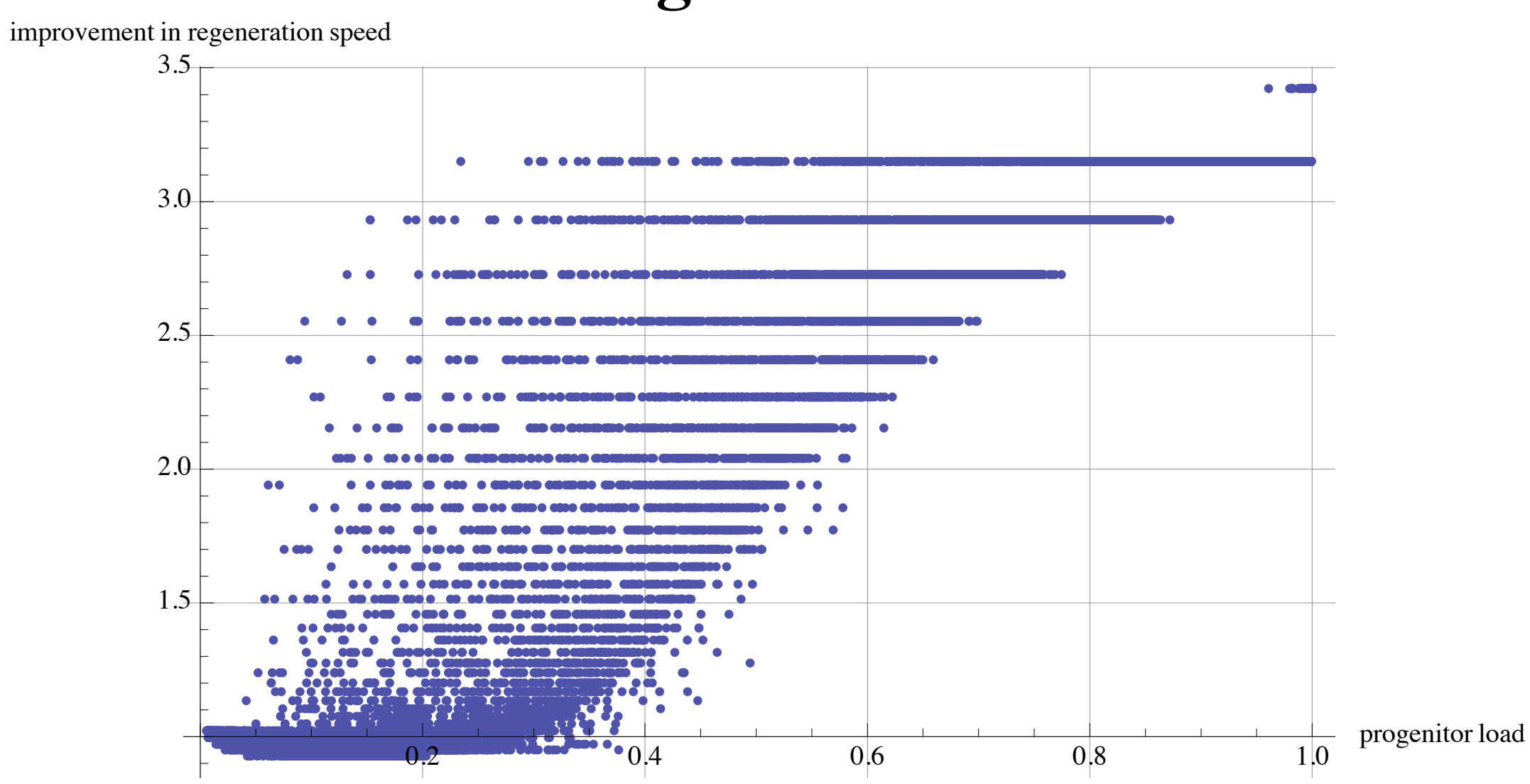

This shows how regeneration speed improvement depends on having a high progenitor load. The horizontal gaps in the distributions of points are an artifact of the fact that t[i] values were extracted from plots of the data, and as such are skewed according to the times chosen automatically by *Mathematica* for plotting.

Next, let's look at the relationship between regeneration speed improvement and the value of p_1. When we look at all cases, there's no impressive correlation.

```
params = {p, δ, ξ, η};
wholeset = Join[params, {f[1], f[2], f[3], css1, css2, t[1], t[2], t[3], sen}];
Valu[list_, symbol_] := First[Part[list, Position[wholeset, symbol][[1]]]];
ListPlot[Map[{Valu[#, p], Log[5] / (Valu[#, δ] Valu[#, t[1]])} &, ToExpression[Import["2V1", "List"]]],
 PlotRange → All, GridLines → Automatic,
 AxesLabel → {"p1", "improvement in regeneration speed"},
 ImageSize → 500, PlotLabel → Style["Figure S15", Bold, Large]]
```

Figure S15

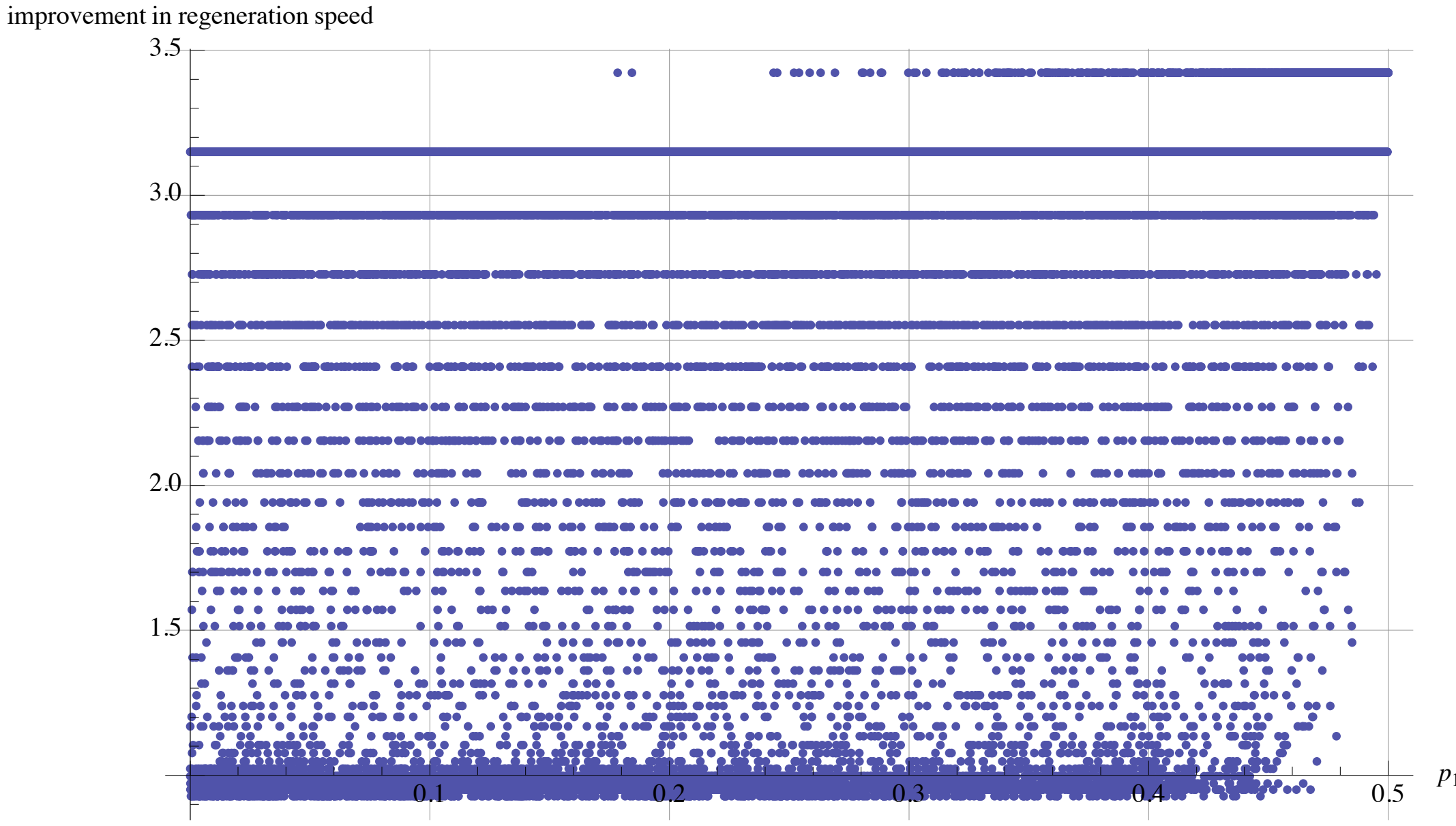

When we look at only those cases with progenitor loads <0.5, we see a strong corelation between high p-values and strong regeneration improvement.

```
params = {p, δ, ξ, η};
wholeset = Join[params, {f[1], f[2], f[3], css1, css2, t[1], t[2], t[3], sen}];
Valu[list_, symbol_] := First[Part[list, Position[wholeset, symbol][[1]]]];
ListPlot[Map[{Valu[#, p], Log[5]/(Valu[#, δ] Valu[#, t[1]])} &,
  Select[ToExpression[Import["2V1", "List"]], (1 + Valu[#, css1])/(1 + Valu[#, css1] + Valu[#, css2]) < 0.5 &]],
 PlotRange → All, GridLines → Automatic,
 AxesLabel → {"p1", "improvement in regeneration speed"},
 ImageSize → 500, PlotLabel → Style["Figure S16", Bold, Large]]
```

Figure S16

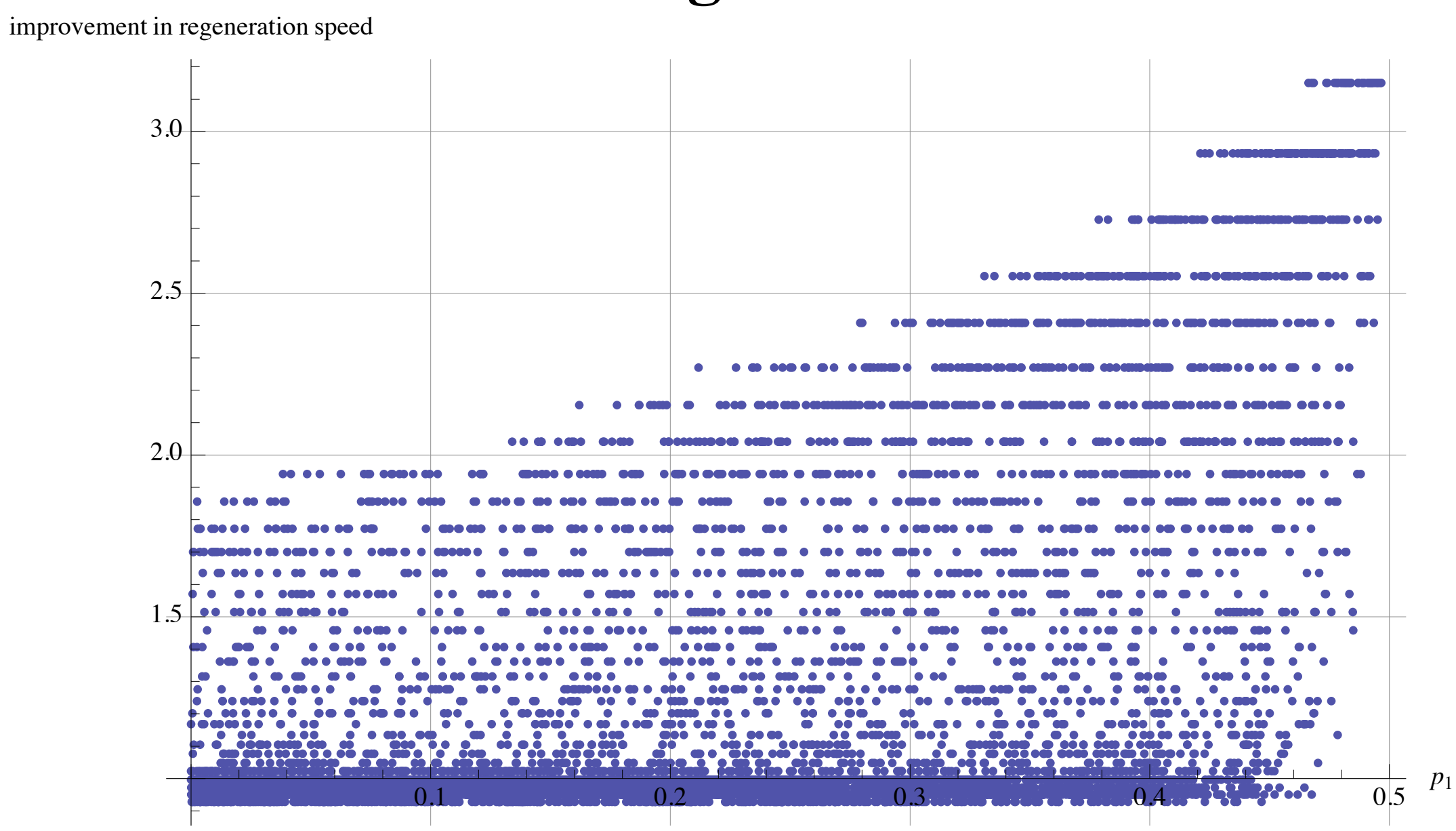

When we look at only those cases with progenitor loads <0.1, we see that only cases with p_1 close to 0.5 show any significant speed up. These cases will, of course, be very sensitive to small changes in p_1

```
params = {p, δ, ξ, η};
wholeset = Join[params, {f[1], f[2], f[3], css1, css2, t[1], t[2], t[3], sen}];
Valu[list_, symbol_] := First[Part[list, Position[wholeset, symbol][[1]]]];
ListPlot[Map[{Valu[#, p], Log[5]/(Valu[#, δ] Valu[#, t[1]])} &,
  Select[ToExpression[Import["2V1", "List"]], (1 + Valu[#, css1])/(1 + Valu[#, css1] + Valu[#, css2]) < 0.1 &]],
 PlotRange → All, GridLines → Automatic,
 AxesLabel → {"p1", "improvement in regeneration speed"},
 ImageSize → 500, PlotLabel → Style["Figure S17", Bold, Large]]
```

Figure S17

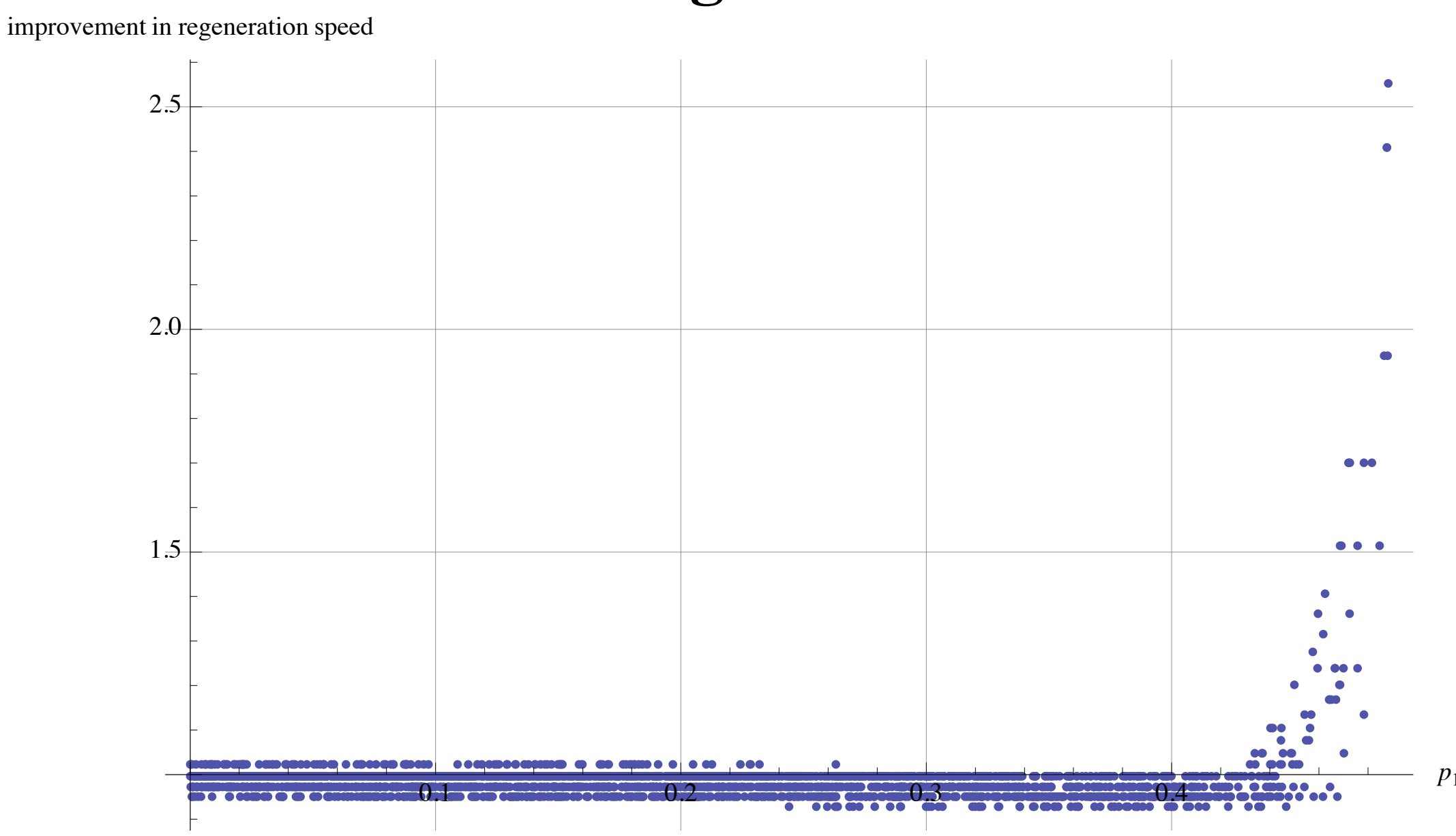

- ***Next we consider the case with feedback only on p_1 and v_1. The file with the parameter space exploration of that model (see previous section) was named "2P1V1" and is read in and analyzed by the following code:***

```
params = {p, δ, ξ, γ, η};
wholeset = Join[params, {f[1], f[2], f[3], css1, css2, t[1], t[2], t[3], sen}];
Valu[list_, symbol_] := First[Part[list, Position[wholeset, symbol][[1]]]];
ListLogPlot[Map[{Valu[#, sen], Log[5] / (Valu[#, δ] Valu[#, t[1]])} &,
  ToExpression[Import["2P1V1", "List"]]], PlotRange → {0, 50},
 AxesLabel → {"Sensitivity to χ0", "improvement in regeneration speed"},
 ImageSize → 500, PlotLabel → Style["Figure S18", Bold, Large]]
```

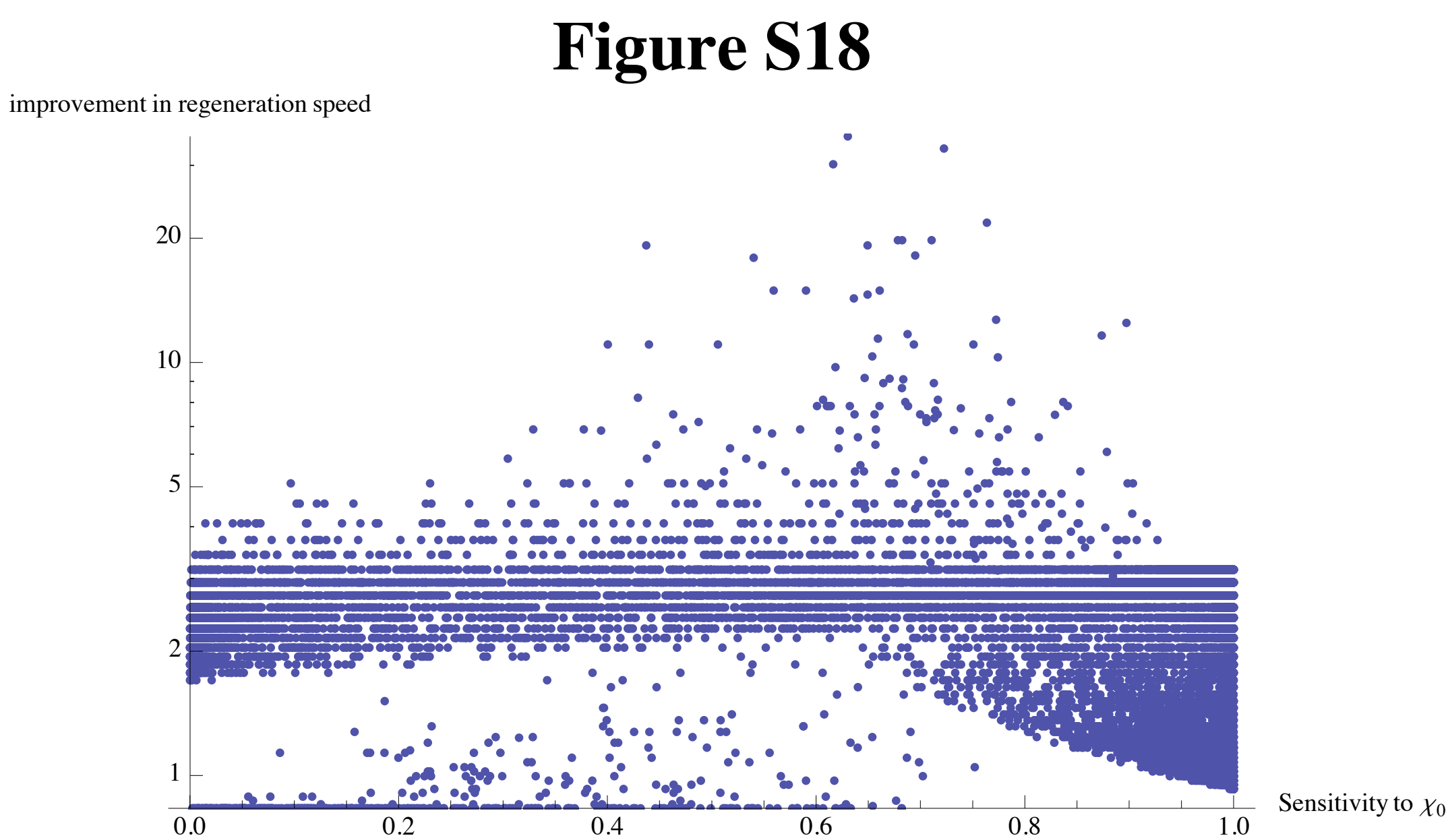

Comparing this with Figure 5a, we see that the cases of very low sensitivity to $\chi_0$now have improvements in regeneration speed that are clustered around 3, and occasionally get close to 5. So this is some improvement on the case with feedback only on P1.

- ***Next we consider the case with feedback only on p_0. The file with the parameter space exploration of that model (see previous section) was named "2P0" and is read in and analyzed by the following code:***

```
params = {p0, p1, δ, ξ, j};
wholeset = Join[params, {f[1], f[2], f[3], f[4], css0, css1, css2, t[1], t[2], t[3], t[4]}];
Valu[list_, symbol_] := First[Part[list, Position[wholeset, symbol][[1]]]];
ListPlot[Map[{Log[10, Valu[#, j]], Log[5] / (Valu[#, δ] Valu[#, t[1]])} &,
  ToExpression[Import["2P0", "List"]]], PlotRange → All, GridLines → Automatic,
 AxesLabel → {"log10feedback gain", "improvement in regeneration speed"},
 ImageSize → 500, PlotLabel → Style["Figure S19", Bold, Large]]
```

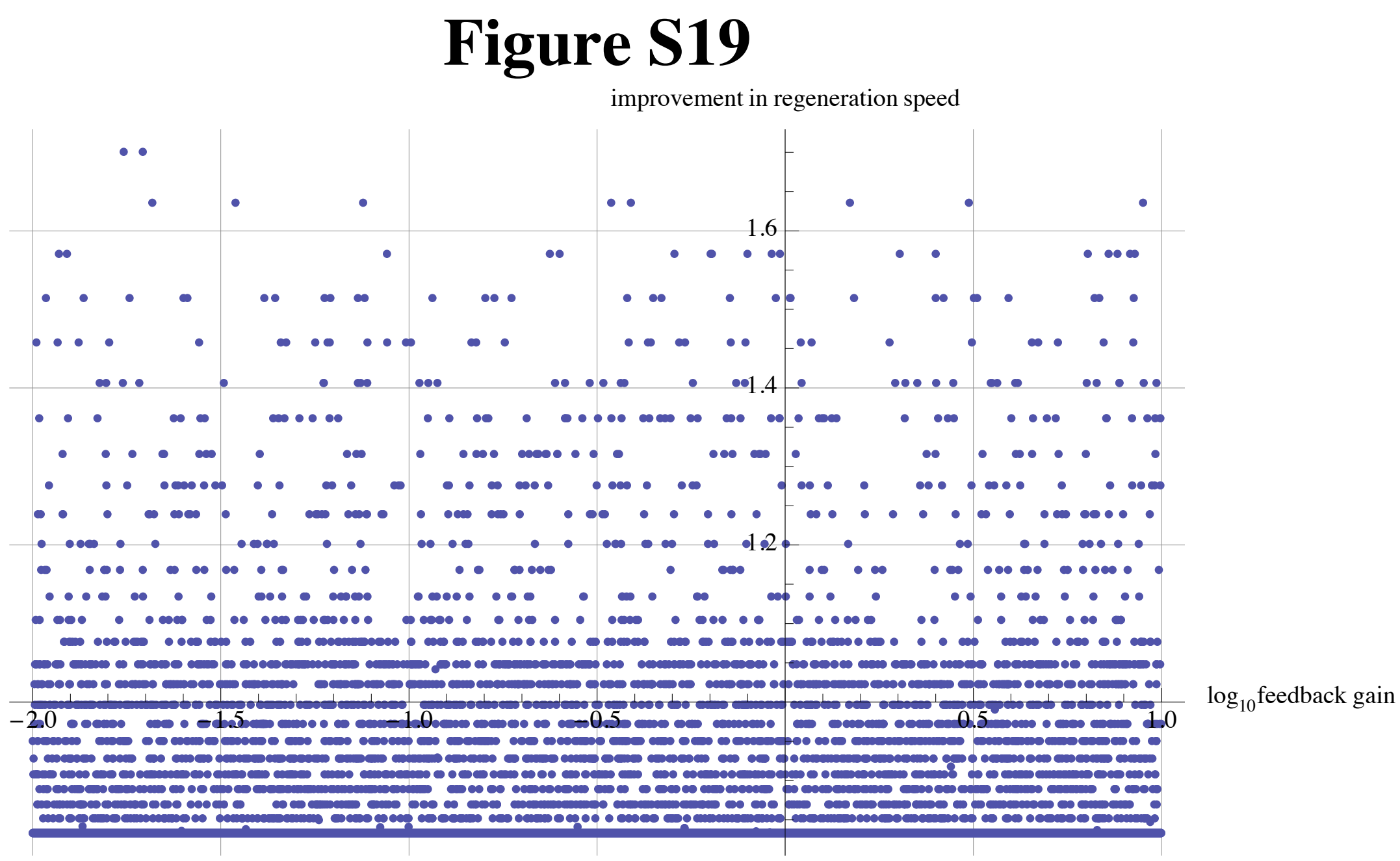

- ***Next we consider the case with feedback only on v_0. The file with the parameter space exploration of that model (see previous section) was named "2V0" and is read in and analyzed by the following code:***

```
params = {p, δ, ξ, κ};
wholeset = Join[params, {f[1], f[2], f[3], css1, css2, t[1], t[2], t[3], sen}];
Valu[list_, symbol_] := First[Part[list, Position[wholeset, symbol][[1]]]];
ListPlot[
 Map[{Valu[#, sen], Log[5] / (Valu[#, δ] Valu[#, t[1]])} &, ToExpression[Import["2V0", "List"]]],
 PlotRange → All, GridLines → Automatic, AxesOrigin → {0, 0},
 AxesLabel → {"Sensitivity to χ0", "improvement in regeneration speed"},
 ImageSize → 500, PlotLabel → Style["Figure S20", Bold, Large]]
```

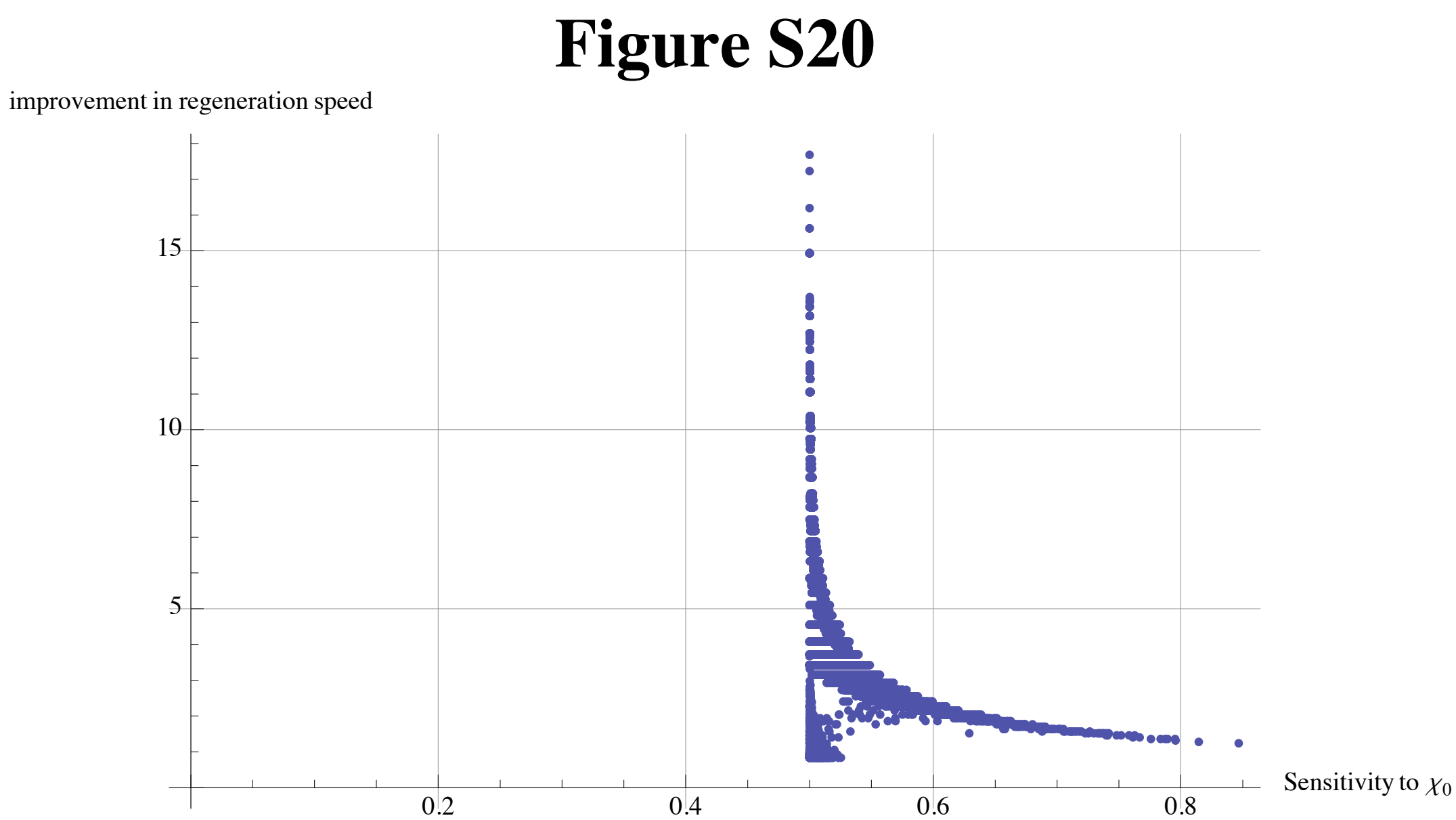

This case is capable of producing good regeneration speed, but since there is no feedback on $p_0$the lowest possible sensitivity to $\chi_0$is 0.5.

- ***Next we consider the case with feedback only on p_0 and v_0. The file with the parameter space exploration of that model (see previous section) was named "2P0V0" and is read in an analyzed by the following code:***

```
params = {p0, p1, δ, ξ, j, k};
wholeset = Join[params, {f[1], f[2], f[3], f[4], css0, css1, css2, t[1], t[2], t[3], t[4]}];
Valu[list_, symbol_] := First[Part[list, Position[wholeset, symbol][[1]]]];
ListPlot[Map[{Log[10, Valu[#, j]], Log[5]/(Valu[#, δ] Valu[#, t[1]])} &,
  ToExpression[Import["2P0V0", "List"]]], PlotRange → All, GridLines → Automatic,
 AxesLabel → {"log10feedback gain", "improvement in regeneration speed"},
 ImageSize → 500, PlotLabel → Style["Figure S21", Bold, Large]]
```

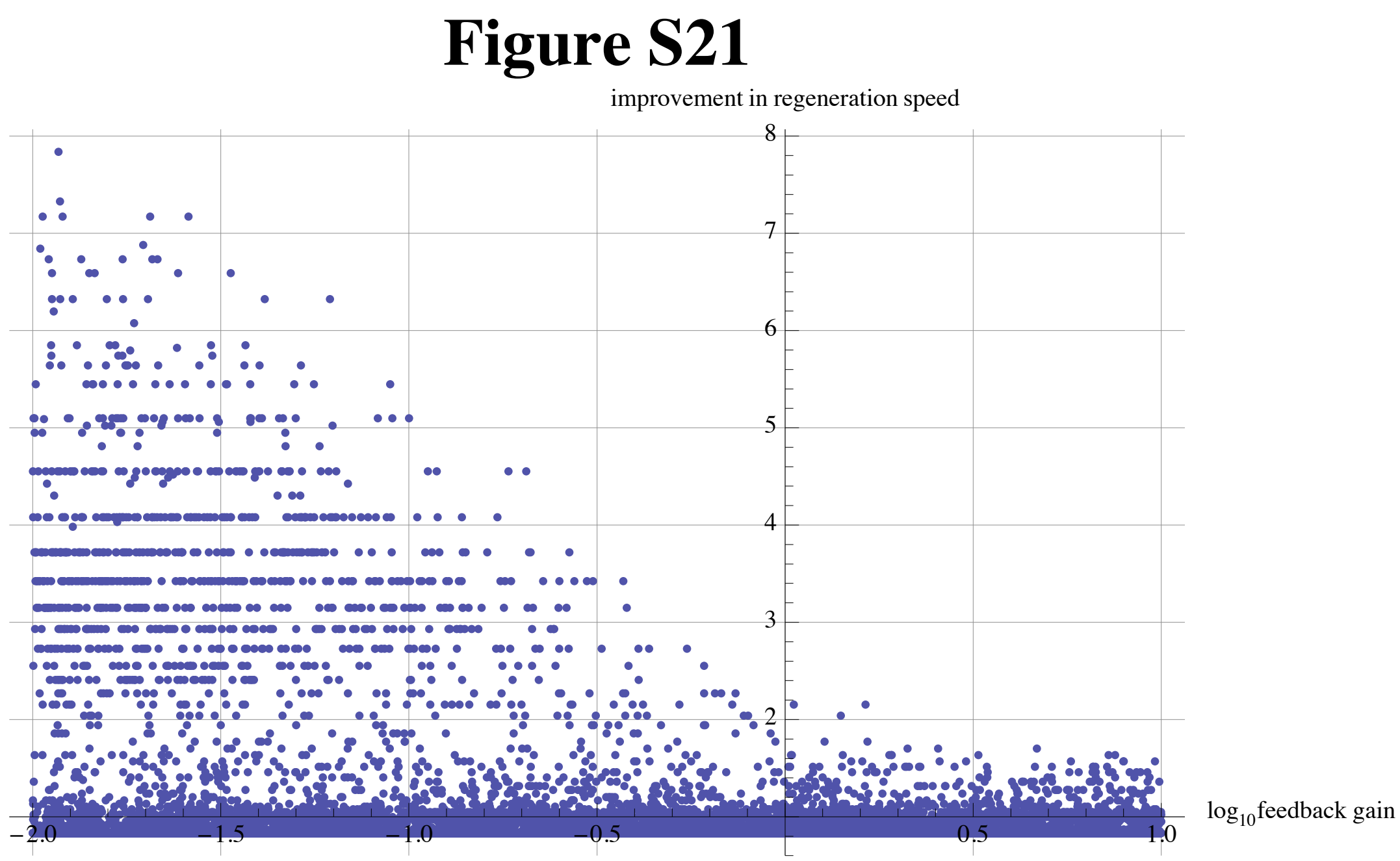

What we see here is that, with feedback onto both $p_0$and $v_0$the possibility of a large improvement in regeneration speed suddenly exists, and since there is feedback on p_0, all cases are perfectly robust to χ_0 (and δ and v_0). However, the following shows that, just as was true when feedback was on $p_1$or p_1 and v_1, those cases with good increase in regeneration speed, all display a decrease in the ratio of t[1]/t[3], which means that regeneration from a 75% depletion is substantially slower than regeneration from a 100% depletion.

```
params = {p0, p1, δ, ξ, j, k};
wholeset = Join[params, {f[1], f[2], f[3], f[4], css0, css1, css2, t[1], t[2], t[3], t[4]}];
Valu[list_, symbol_] := First[Part[list, Position[wholeset, symbol][[1]]]]; ListLogLogPlot[
 Map[{Log[5]/(Valu[#, δ] Valu[#, t[1]]), Valu[#, t[1]]/Valu[#, t[3]]} &, ToExpression[Import["2P0V0", "List"]]],
 PlotRange → All, AxesLabel → {"ln5/(δ t[1])", "t[1]/t[3]"}, AxesOrigin → {0.8, 10^0.085523},
 ImageSize → 500, PlotLabel → Style["Figure S22", Bold, Large]]
```

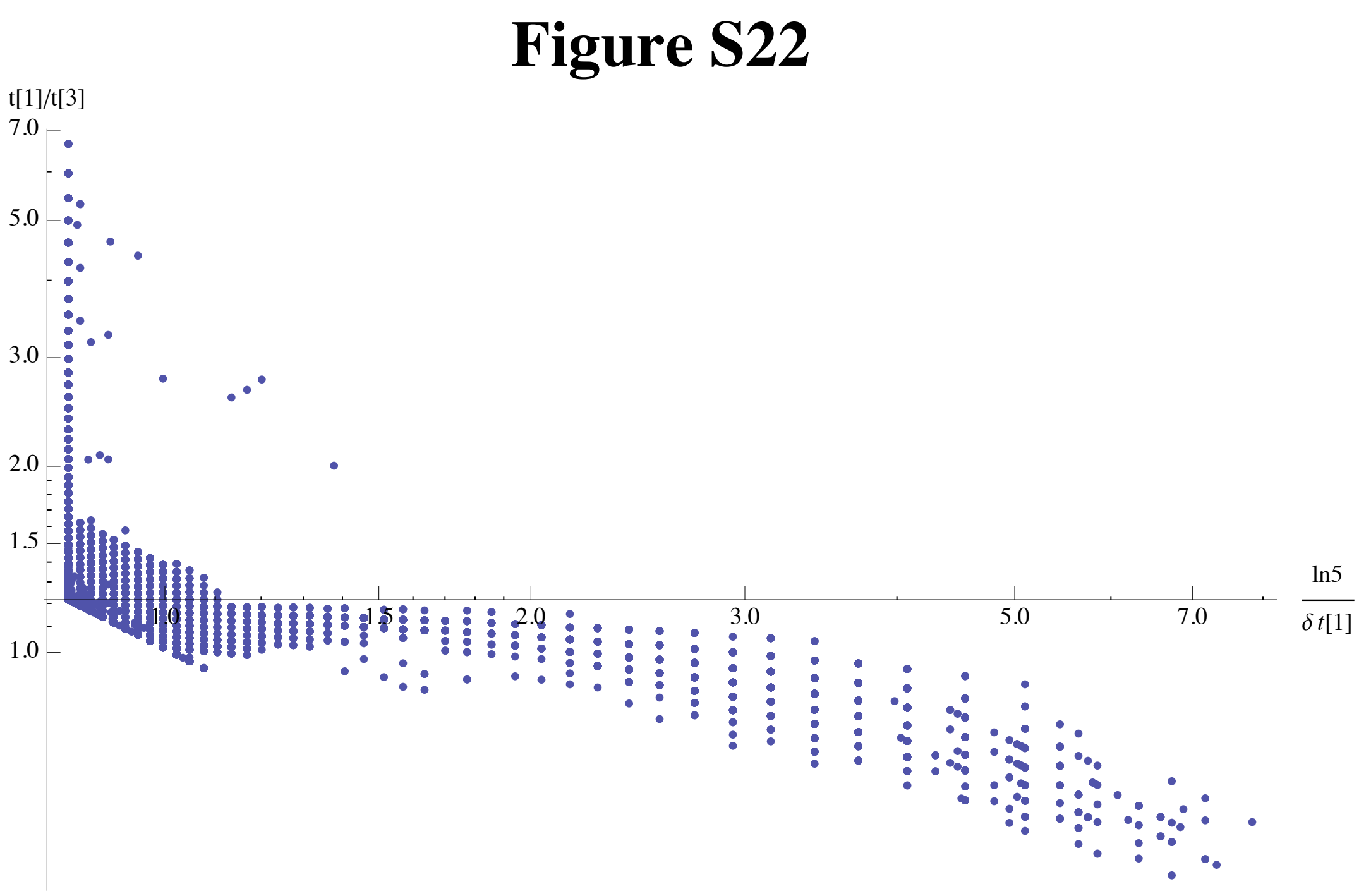

10. Simulation of pulse - chase experiment (Fig. S23)

```
Clear[σ]
```

The following code was used to simulate of the pulse chase experiment in Figure 4

```
t1 = Table[α0 = 5; β0 = 5;  σ0 = 5;  δ0 = 0;
   n0 = 500; α1 = 6; β1 = 5;  σ1 = 6;  δ1 = (9.69 * 0.23 * 10^loggdf)/(1 + 0.23 * 10^loggdf); n1 = 4000;
   π1 = 12;  π2 = 14; λn_ = αn + βn + σn + δn;
   p0 = 0.2; p1 = 0.30/(1 + 15 * 10^loggdf);
   Mashlist = RandomReal[{σ0, β0 + σ0}, n0];
list1 = RandomReal[{0, α1 + σ1 + β1}, n1];
list4 = Select[list1 - π2, #1 < 0 &] + α1 + σ1 + β1 + δ1;
list6 = Join[list4, list4, Select[list1 - π2, #1 ≥ 0 &]];
INPlist = Select[list6, 0.5 + σ1 - π2 + π1 ≤ #1 ≤ -0.5 + β1 + σ1 &];
   ORNlist = {}; ORNtally = {}; Allcelltally = {};
   timecounter = 14; i = 0.1;
   Do[MASHdividerlist = Table[λ0, {2 p0 Length[Select[Mashlist, #1 ≤ 0 &]]}];
    MASHdifferentiatorlist = Table[λ1, {2 (1 - p0) Length[Select[Mashlist, #1 ≤ 0 &]]}];
    INPdividerlist = Table[λ1, {2 p1 Length[Select[INPlist, #1 ≤ 0 &]]}];
    INPdifferentiatorlist = Table[0, {2 (1 - p1) Length[Select[INPlist, #1 ≤ 0 &]]}];
    Mashlist = Join[Select[Mashlist, #1 > 0 &], MASHdividerlist] - i;
    INPlist = Join[Select[INPlist, #1 > 0 &], MASHdifferentiatorlist, INPdividerlist] - i;
    ORNlist = Join[ORNlist, INPdifferentiatorlist] + i; timecounter = timecounter + i;
    Allcelltally = Append[Allcelltally, Length[Mashlist] + Length[INPlist] + Length[ORNlist]];
    ORNtally = Append[ORNtally, Length[ORNlist]];,
    {340}];
   {{loggdf, 100 * ORNtally[[160]]/Allcelltally[[160]]},
    {loggdf, 100 * ORNtally[[340]]/Allcelltally[[340]]}}, {loggdf, -2, 2, 0.2}];
plot30 = ListPlot[Map[First, t1], Joined → True, AxesLabel → {"Log[GDF11]", "%NCAM+"},
   AxesOrigin → {-2, 0}, PlotRange → All, PlotStyle → {Thickness[0.005], Black},
   PlotLabel → Style["Figure S23", Bold, Large]];
plot48 = ListPlot[Map[Last, t1], Joined → True, AxesOrigin → {-2, 0},
   PlotRange → All, PlotStyle → {Thickness[0.005], Blue}];
λn_ =.; p0 =.; p1 =.; α0 =.; β0 =.; σ0 =.; δ0 =.; n0 =.; α1 =.; β1 =.; σ1 =.; δ1 =.; n1 = .;
π1 =.; π2 =.;
Show[plot30, plot48, AspectRatio → 1]
```

Figure S23

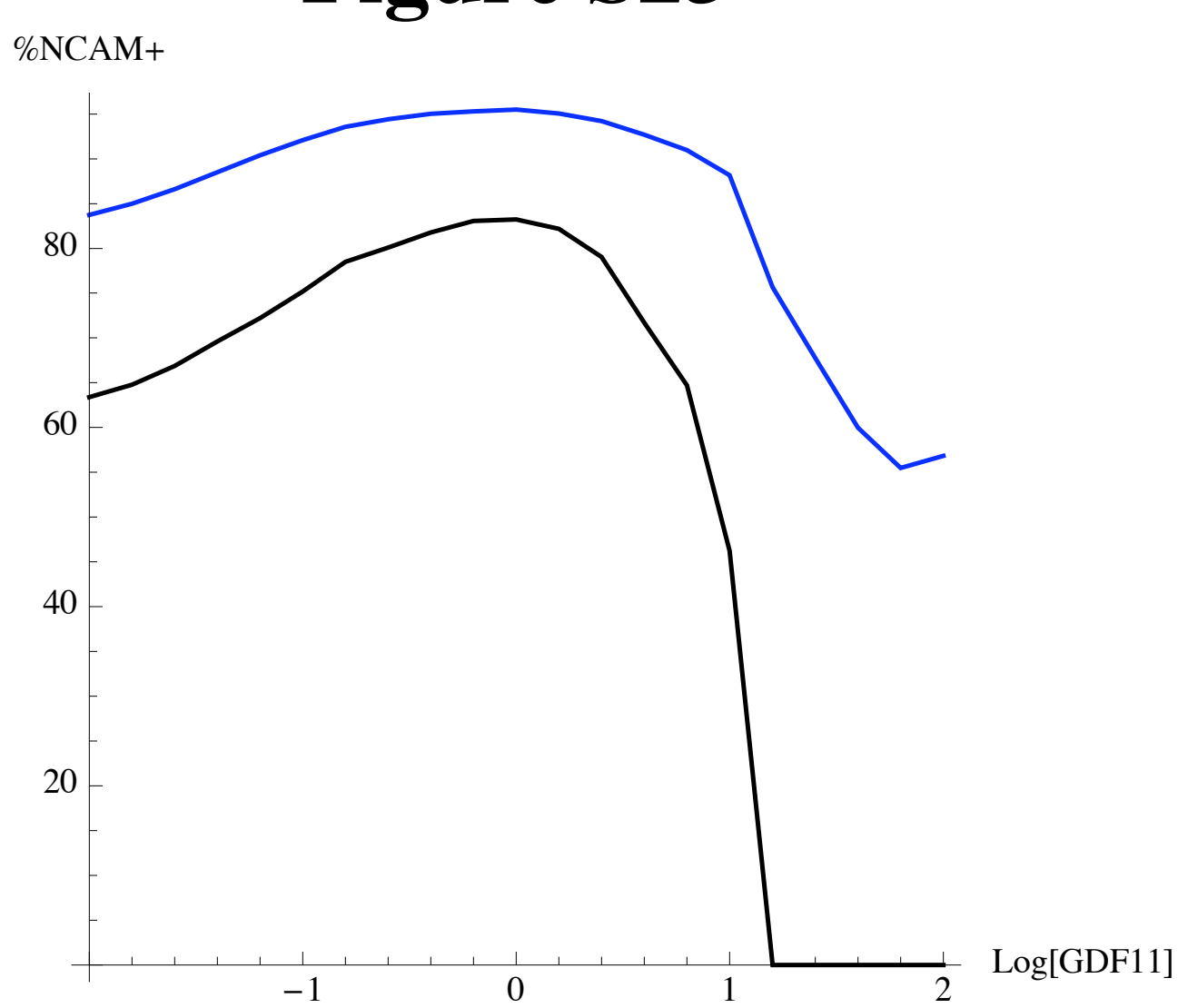

Below the code is annotated for the purpose of explanation. We begin with the three stage model of an unbranched lineage, in which the second stage in the INP and the last stage is the ORN. We assume that explantation of the OE into culture removes all feedback (secreted molecules will be diluted by the medium).

```
(*The main function of the code is to create a table of pairs of data points;
in each pair the first point has a particular value of loggdf in the first position,
and a value of the percentage of all BrdU labeled cells that is NCAM+ in the second
   position.  The first point in each pair corresponds to data at t=160 time steps,
and the second point t=340 time steps.  Since our time steps will be 0.1 hours,
this means 16 and 34 hours after then end of the BrdU pulse,
which is the same as 18 and 36 hours after its beginning*)
t1 = Table[α0 = 5; β0 = 5;  σ0 = 5;  δ0 = 0;

   n0 = 500; α1 = 6; β1 = 5;  σ1 = 6;  δ1 = (9.69 * 0.23 * 10^loggdf)/(1 + 0.23 * 10^loggdf); n1 = 4000;

   π1 = 12;  π2 = 14; λn_ = αn + βn + σn + δn;

   p0 = 0.2; p1 = 0.30/(1 + 15 * 10^loggdf);
   (*these define various parameter values:
     π1 and π2 are the times of the start and stop of the pulse of BrdU.(we assume the
         pulse occurs no more than 1 cell cycle after the start of the experiment)

For Mash1/Sox2 and INP cells (denoted by subscripts 1 and 2, respectively),
   we separately specify lengths of the G1,S and G2M phases of the cell cycle.  We
    represent these by α,β,and σ,respectively.Thus,the cell cyle length λ = α+σ+β.;

   However,we shall consider that treatment with GDF11 potentially lengthens the cell
    cycle by adding an extra time period into the G1 phase.We shall call this
    δ.  Thus when GDF11 is present, the length of the cell cycle will be α+σ+β+δ.;
   We let n1 and n2 represent the initial numbers of Mash1/Sox2 cells and INPs respectively

    The values chosen for n1 and n2 reflect the approximate ratio of cells that are Mash1 vs
    INP and incorporate BrdU at the time of the pulse, about 12 hours in culture

     The value chosen for δ2 reflects the results of a pulse-
    fix experiment (one in which cells are fixed immediatedly after a short BrdU pulse),
   from which we may infer how much additional time GDF11 adds to the cell cycle,
   as a function of GDF11.  The parameter loggdf is means to represent log10[GDF11]

    p1andp2represent replication probabilities for the two cell types.  The
    expression used for p1 is a Hill function, in which GDF11 feeds back on p1
    without cooperativiey.  The parameters within it are fit to the data manually
   *)
   Mashlist = RandomReal[{σ0, β0 + σ0}, n0];
list1 = RandomReal[{0, α1 + σ1 + β1}, n1];
   (*We begin by generating some lists where every entry in the list is the cell cycle
      position of a single cell.  Note that we identify positions counting backwards from M-
     phase.  Thus a cell cycle position of σ means a cell at the start of
      G2.  A cell cycle position of σ+β=a cell at the start of S phase.

        Since this is a pulse-chase experiment,
   we start off our lists by defining a cohort of labeled cells that were
     in S phase during the pulse.We do this by starting our lists at time=π2,
   the end of the pulse,and picking out those that are BrdU labeled.For Mash1/stem cells,
   this number atn1.For INPs,it is n2.

      our starting list of labeled Mash1 cells has cell cycle positions from σ to σ+β,
   because they are in S-phase

     In generating the cell cycle positions of the INPs,
   we take note of the fact that between plating and pulsing, existing INPs are
    replicating and giving rise to neurons, and Mash1 cells are giving rise to INPs.
```

```
     list1 is just a list of random positions about an entire normal cell cycle.

    *)
list4 = Select[list1 - π2, #1 < 0 &] + α₁ + σ₁ + β₁ + δ₁;
   (*All cells in list1 are then advanced the time between
    plating and the end of the pulse, the those with negative values
    (i.e. that have traversed M) are selected out and given the name "list4" *)
list6 = Join[list4, list4, Select[list1 - π2, #1 ≥ 0 &]];
   (*list 4 is then duplicated (to account for cell division) and
    added back to the remainder of list 1.  The result is called list 6.

    list 6 represents the cell cycle positions of all the cells that
    have been produced by INPs during the period up to the end of the pulse*)
INPlist = Select[list6, 0.5 + σ₁ - π2 + π1 ≤ #1 ≤ -0.5 + β₁ + σ₁ &];
   (*To make the INPlist, we select from list 4 those cells whose positions lie between 0.5+
   σ₂-π2+π1 and -0.5+β₂+σ₂.  The second condition says that they have to be at least
     0.5 hours into S by the end of the pulse.  The first condition says that,
   at the start of the pulse they were within 0.5 hour of the end of S.  So
    these are all the cells in list6 that would get pulsed*)
   ORNlist = {}; ORNtally = {}; Allcelltally = {}; timecounter = 14; i = 0.1;
   (*here we are just initializing some values we'll need later*)
   Do[MASHdividerlist = Table[λ₀, {2 p₀ Length[Select[Mashlist, #1 ≤ 0 &]]}];
    MASHdifferentiatorlist = Table[λ₁, {2 (1 - p₀) Length[Select[Mashlist, #1 ≤ 0 &]]}];
    INPdividerlist = Table[λ₁, {2 p₁ Length[Select[INPlist, #1 ≤ 0 &]]}];
    INPdifferentiatorlist = Table[0, {2 (1 - p₁) Length[Select[INPlist, #1 ≤ 0 &]]}];
    Mashlist = Join[Select[Mashlist, #1 > 0 &], MASHdividerlist] - i;
    INPlist = Join[Select[INPlist, #1 > 0 &], MASHdifferentiatorlist, INPdividerlist] - i;
    ORNlist = Join[ORNlist, INPdifferentiatorlist] + i; timecounter = timecounter + i;
    Allcelltally = Append[Allcelltally, Length[Mashlist] + Length[INPlist] + Length[ORNlist]];
    ORNtally = Append[ORNtally, Length[ORNlist]];,
    {340}];
   (*The above Do loop does the following things for 340 time steps of "i" hours:

       1. It counts up the number of Mash1 cells that crossed the
        M/G1 boundary during the last time step.  It multiplies that by p1,
     and adds twice that number (to account for division) into a list,
     "MASHdividerlist" that represents progeny of MASH1 cells that remain
       the same type.  It gives them all the cell cycle position=λ₁,
     because that is the position of a cell at the start of G1.
       2.  It creates a second list, "MASHdifferentiatorlist",
     in the same way, using 1-p₁ for the probability,
     and setting the cells to position=λ₂, since they are now INPs.
       3.  Similar rules are used to create and INPdividerlist and an INP
      differentiatorlist.  However, when INPs differentiate into ORNs,
     the ORNs are assigned an initial cell cycle position of "0".  For these cells,
     the decline over time in this number will provide the
      information about how long ago each ORN differentiated.
       4. Mashlist is now modified by removing from it any cells that have
      traversed the G2M boundary, adding back in the cells in Mashdivider list,
     and reducing every cell's index by i, to represent the passage of i hours.
       5. The INPlist is modified in a similar way, but now there are two sources of INPs,
     Mashdifferentiatorlist and INPdividerlist.
       6. The ORNlist, which started as an empty set,
     is modified by adding in the INPdifferentiatorlist.  "i" is added to each entry
      in this list so the entry counts upward the time since differentiation.
       7.  Timecounter is incremented by i.
       8.  ORNtally keeps track of the number of ORNs at each timepoint,
     by appending (timecounter, Length[ORNlist]} to ORNtally with every time step.  *)
   {{loggdf, 100 * ORNtally[[160]] / Allcelltally[[160]]}, {loggdf, 100 * ORNtally[[340]] / Allcelltally[[340]]}},
   {loggdf, -2, 2, 0.2}];
(*The above instructions, which end the Table command,
```

```
create the pairs of points referred to at the start of the annotations,
over a range of [GDF11] from 10^-2 to 100 ng/ml, in steps of 0.2*)
plot30 = ListPlot[Map[First, t1], Joined → True, AxesLabel → {"Log[GDF11]", "%NCAM+"},
   AxesOrigin → {-2, 0}, PlotRange → All, PlotStyle → {Thickness[0.005], Black}];
plot48 = ListPlot[Map[Last, t1], Joined → True, AxesOrigin → {-2, 0},
   PlotRange → All, PlotStyle → {Thickness[0.005], Blue}];
λn_ =.; p0 =.; p1 =.; α0 =.; β0 =.; σ0 =.; δ0 =.; n0 =.; α1 =.; β1 =.; σ1 =.; δ1 =.; n1 = .;
π1 =.; π2 =.;
Show[plot30, plot48, AspectRatio → 1]
(*The above commands simply plot to points,
connecting consecutive ones.  The command in the penultimate line simply clears a lot of
 definitions so they do not interefere with later work in the same Mathematica session*)
```

11 Spatial dynamics calculations (Fig. S24-S31)

Consider an epithelium of semi - infinite extent, so that to model diffusion of molecules we need only consider a single dimension (the apico-basal dimension). Define a coordinate system in which x=0 represents the basal lamina, and x=-xmin represents the apical surface. x>0 then represents the stroma underlying the epithelium. Assume that at the apical surface there are tight junctions, so that diffusing molecules may not leave, whereas at the basal lamina there is no barrier to molecular diffusion.

If a secreted, diffusive molecule is made uniformly throughout the epithelium at constant rate v, we can calculate its steady state concentration along the apicobasal axis. To do this we let d stand for the effective diffusion coefficient of the molecule, and k stand for its degradation rate constant. Because k may be different in the epithelium versus the stroma, we use k_L and k_R to represent epithelial and stromal degradation rate constants, respectively, on the "left" (epithelium) and "right" (stroma).

We may use a single ODE to represent the steady state solution for this situation, in which the variable a[x] is the concentration of the factor in space. In order to have boundary conditions at two ends, we define a "dummy point", xmax>0, where we will set the concentration of the factor to zero. We will later let xmax go to infinity, so that the result corresponds to an "open-ended" stroma.

```
sys = {If[x > 0,
    0 == dL a''[x] + v - kL a[x],
    0 == dR a''[x] + -kR a[x]],
   a'[-xmin] == 0, a[xmax] == 0};
```

We can simplify this by defining some lumped parameters,

$\lambda_L = \sqrt{k_L/d_L}$; $\lambda_R = \sqrt{k_R/d_R}$; and $\nu = v/\left(2\lambda_L{}^2 d_L\right)$.

The λ parameters are length constants (units of length^{-1}) and represent the inverse of the decay lengths of the diffusing factor in the epithelium or stroma. λ^{-1} may be understood as how far the average diffusing molecule travels before it is degraded.

Note that since $\lambda_L{}^2 = k_L/d$, $\nu = v/2k_L$;

Making these substitution, we may express the ODE as :

```
sys2 = {If[x > 0, 2 ν λL^2 - λL^2 a[x] + a''[x] == 0, -λR^2 a[x] + a''[x] == 0], a'[-xmin] == 0, a[xmax] == 0};
```

To solve this discontinuous system, we solve separately in the epithelium and stroma, and the require continuity of a[x] and a'[x] at x=0. We get continuity of a[x] at x=0 by defining α as the value of a[x] at x = 0, and using it as a boundary condition for both solutions.

First we solve in the stroma:

```
rightsol =
 a[x] /. DSolve[{0 == a''[x] - λR^2 a[x] , a[0] == α, a[xmax] == 0}, a[x], x] // Simplify // Flatten
```

$$\left\{\frac{e^{-x\lambda_R}\left(-e^{2x\lambda_R}+e^{2\,\mathrm{xmax}\,\lambda_R}\right)\alpha}{-1+e^{2\,\mathrm{xmax}\,\lambda_R}}\right\}$$

Now we let xmax go to infinity

```
rightsol1 = Assuming[ν > 0 && xmax > 0 && λR > 0 && xmin > 0 && α > 0,
   Limit[rightsol, xmax → Infinity]] // FullSimplify
```

$$\left\{e^{-x\lambda_R}\,\alpha\right\}$$

This simply gives the well known results that , in the region where the factor is not produced, its gradient is a simple exponential. Now we solve in the epithelium

```
leftsol1 = a[x] /. DSolve[{0 == a''[x] - λL^2 a[x] + 2 ν λL^2, a[0] == α, a'[-xmin] == 0}, a[x], x] //
   Simplify // Flatten
```

$$\left\{\frac{e^{-x\lambda_L}\left(\left(1+e^{2(x+xmin)\lambda_L}\right)\alpha-2\left(-1+e^{x\lambda_L}\right)\left(-1+e^{(x+2\,xmin)\lambda_L}\right)\nu\right)}{1+e^{2\,xmin\,\lambda_L}}\right\}$$

Finally we require continuity of a'[x] at x = 0

```
{leftsol2, rightsol2} = {leftsol1, rightsol1} /.
   Flatten[Solve[(D[rightsol1, x] == D[leftsol1, x]) /. x → 0, α]] // FullSimplify
```

$$\left\{\left\{\frac{2\,e^{-x\lambda_L}\,\nu\left(e^{x\lambda_L}\left(-1+e^{2\,xmin\,\lambda_L}\right)\lambda_L-\left(-1+e^{x\lambda_L}\right)\left(-1+e^{(x+2\,xmin)\lambda_L}\right)\lambda_R\right)}{-\lambda_L+\lambda_R+e^{2\,xmin\,\lambda_L}\left(\lambda_L+\lambda_R\right)}\right\},\right.$$
$$\left.\left\{\frac{2\,e^{-x\lambda_R}\,\nu\,\mathrm{Sinh}[xmin\,\lambda_L]\,\lambda_L}{\mathrm{Sinh}[xmin\,\lambda_L]\,\lambda_L+\mathrm{Cosh}[xmin\,\lambda_L]\,\lambda_R}\right\}\right\}$$

Let us normalize x to λ_L, i.e. define a new length X = x λ_L, Xmin = xmin λ_L. Let's also define ρ to be the ratio λ_L/λ_R.

```
{leftsol3, rightsol3} =
 {leftsol2, rightsol2} /. {x → X / λL, xmin → Xmin / λL} /. λR → λL / ρ // FullSimplify // Flatten
```

$$\left\{2\,\nu-\frac{2\,\nu\,\mathrm{Cosh}[X+Xmin]}{\mathrm{Cosh}[Xmin]+\rho\,\mathrm{Sinh}[Xmin]},\ \frac{2\,e^{-\frac{X}{\rho}}\,\nu\,\rho\,\mathrm{Sinh}[Xmin]}{\mathrm{Cosh}[Xmin]+\rho\,\mathrm{Sinh}[Xmin]}\right\}$$

We see that the parameter ν may be factored out of both of these expressions. Thus, if we consider our units of concentration to be in units of ν, we may simply treat ν as unity.

```
{leftsol4, rightsol4} = {leftsol3, rightsol3} /. ν → 1;

With[{exp1 = leftsol4, exp2 = rightsol4}, Manipulate[
  Show[ Plot[Evaluate[exp1] , {X, -Xmin, 0}, PlotRange → All], Plot[exp2, {X, 0, 4}],
   PlotRange → All, AxesLabel → {"distance", "concentration"}, AxesOrigin → {0, 0}],
  Style["Figure S24", Bold, Large], {{ρ, 0.7}, 10^-6, 10}, {{Xmin, 1}, 0, 4}]]
```

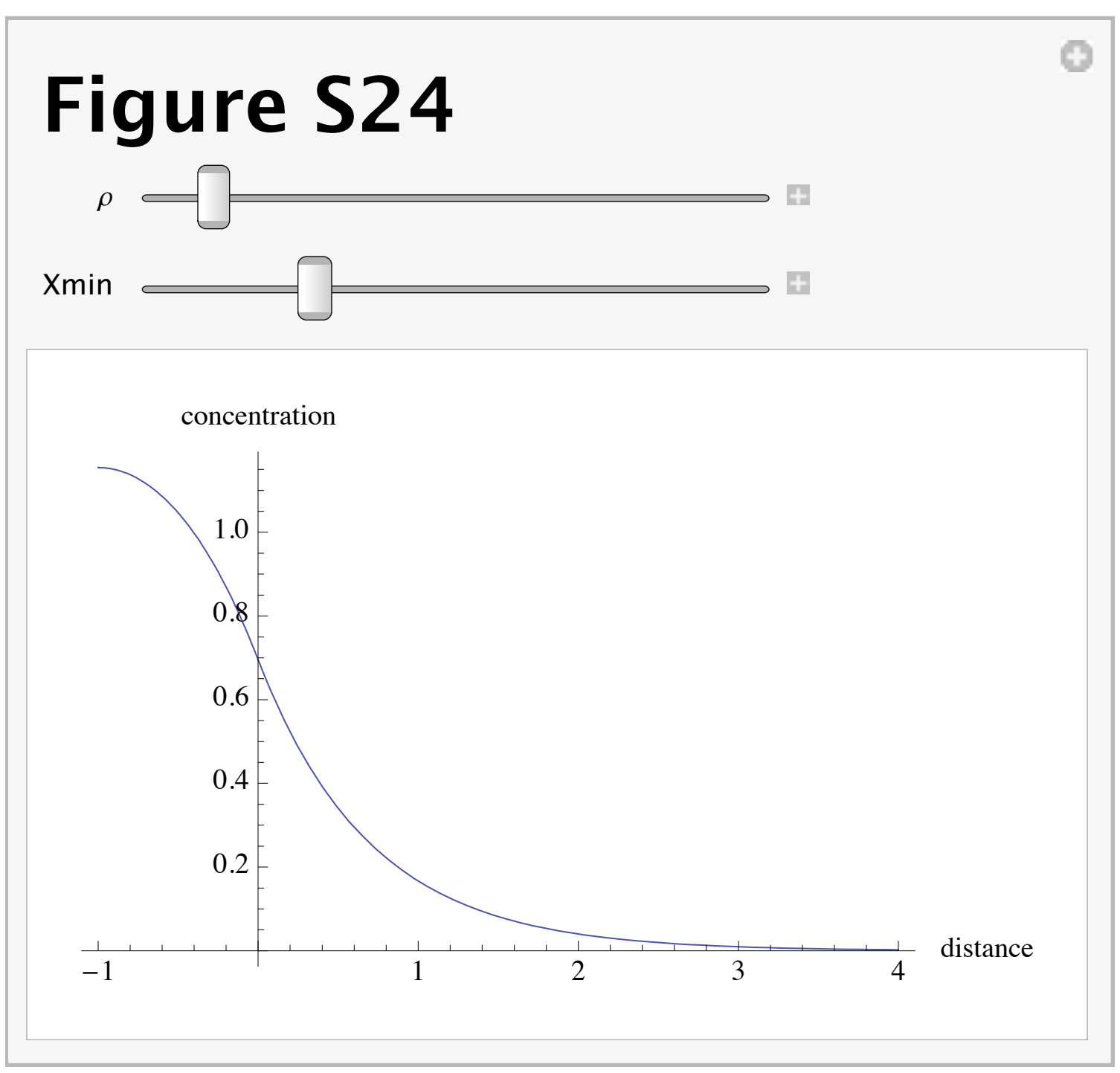

Here we look just at the concentration of the factor in the epithelium, i.e. -Xmin<X<0, as a function of ρ and Xmin. The basement membrane will be on

the right; the apical surface on the left. Concentration is on the ordinate. The sliders allow us to vary the thickness of the epithelium, and ρ.

```
Manipulate[
 Show[Plot[0, {X, -4, 0}, PlotRange → {0, 2}], Plot[2, {X, -Xmin, 0}, Filling → Axis],
  Plot[ (2 - (2 Cosh[X + Xmin])/(Cosh[Xmin] + ρ Sinh[Xmin])), {X, -Xmin, 0}, PlotRange → {0, 2}, Filling → Axis]],
 Style["Figure S25", Bold, Large], {{ρ, 0.2}, 10^-6, 10}, {{Xmin, 1}, 10^-6, 4}]
```

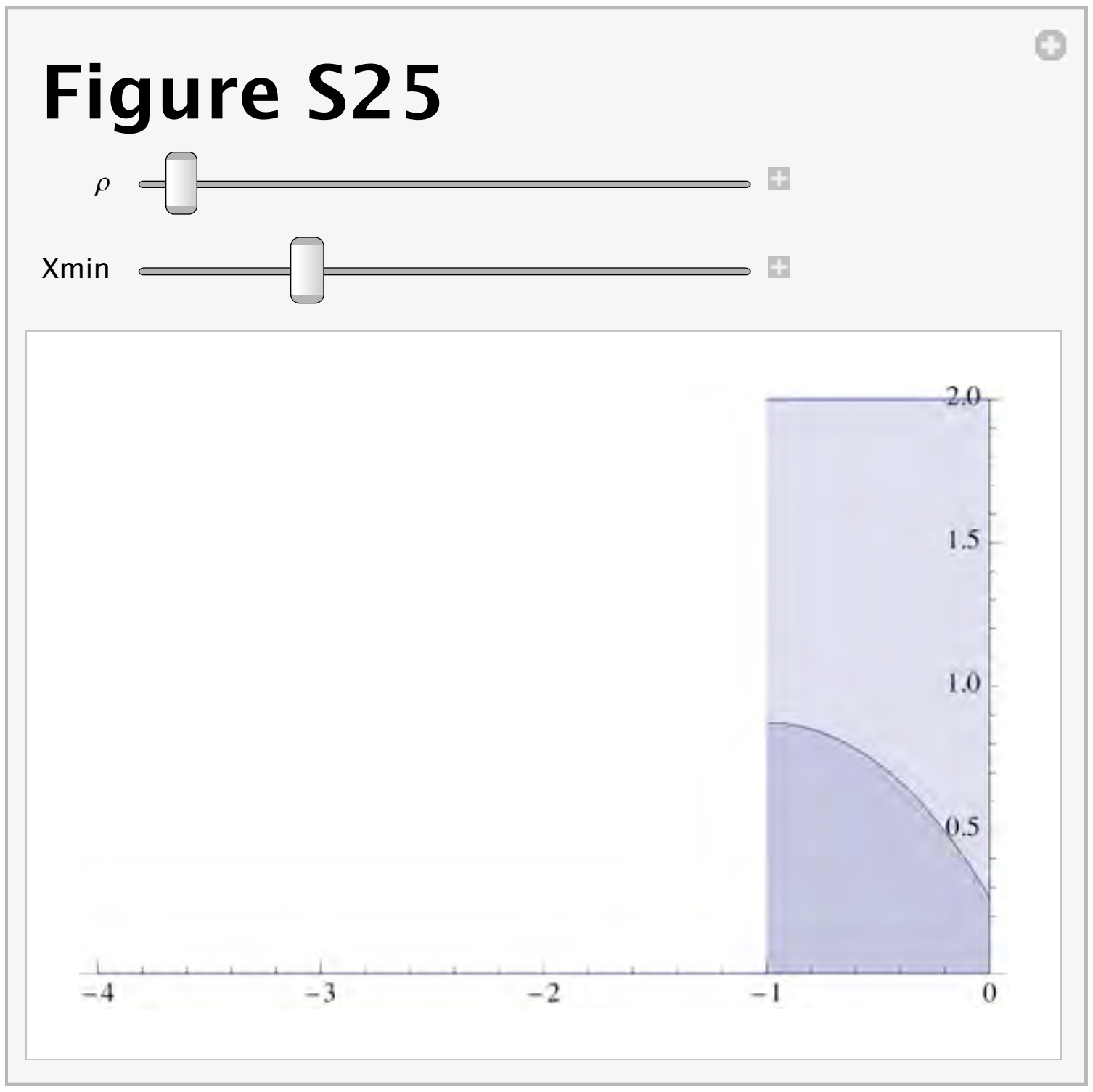

Now that we know the concentration of a[x] in space, we next would like to calculate the amount of a[x] that cells in the epithelium "see". Obviously, how much they see will depend on where they are in the epithelium. So we will consider two scenarios:

- ***Scenario 1 : Cells that respond to a[x] are distributed uniformly in the epithelium***

In this case we need to calculate the average value of a[x]. That means integrating it from 0 to -Xmin and dividing by Xmin.

```
αbar = Assuming[Xmin > 0 && ρ > 0, FullSimplify[ν/Xmin ∫_{-Xmin}^{0} (2 - (2 Cosh[X + Xmin])/(Cosh[Xmin] + ρ Sinh[Xmin])) dX]]
```

$$2\nu - \frac{2\nu\,\mathrm{Sinh}[\mathrm{Xmin}]}{\mathrm{Xmin}\,\mathrm{Cosh}[\mathrm{Xmin}] + \mathrm{Xmin}\,\rho\,\mathrm{Sinh}[\mathrm{Xmin}]}$$

We can equivalently express this as $2\nu\left(1 - \frac{1}{\mathrm{Xmin}\,(\mathrm{Coth}[\mathrm{Xmin}]+\rho)}\right)$;

- ***Scenario 2 : Cells that respond to a[x] are distributed in a fraction of the epithelium close to the basal surface.***

Define $\kappa<1$ as a fraction of the epithelium near the basal surface. To find the average value of a[x] within that zone, we integrate from $-\kappa$ Xmin to zero, and divide by κ Xmin

```
αkappa = Assuming[Xmin > 0 && ρ > 0, FullSimplify[ν/(κ Xmin) ∫_{-κ Xmin}^{0} (2 - (2 Cosh[X + Xmin])/(Cosh[Xmin] + ρ Sinh[Xmin])) dX]]
```

$$\frac{\nu\left(2\,\mathrm{Xmin}\,\kappa + \frac{2\,(-\mathrm{Sinh}[\mathrm{Xmin}]+\mathrm{Sinh}[\mathrm{Xmin}-\mathrm{Xmin}\,\kappa])}{\mathrm{Cosh}[\mathrm{Xmin}]+\rho\,\mathrm{Sinh}[\mathrm{Xmin}]}\right)}{\mathrm{Xmin}\,\kappa}$$

We can use Manipulate to observe how each one behaves

```
With[{term1 = αbar/ν, term2 = αkappa/ν},
 Manipulate[Plot[Evaluate[{Tooltip[term1, "αbar"], Tooltip[term2, "αkappa"]}],
   {Xmin, 0, 6}, PlotRange → {0, Automatic},
   PlotStyle → {{Thick, Red}, {Thick, Blue}}, AxesLabel → {"Xmin", "perceived a"}],
  Style["Figure S26", Bold, Large], {{ρ, 0.1}, 0, 5}, {{κ, 0.1}, 10^-6, 1}]]
```

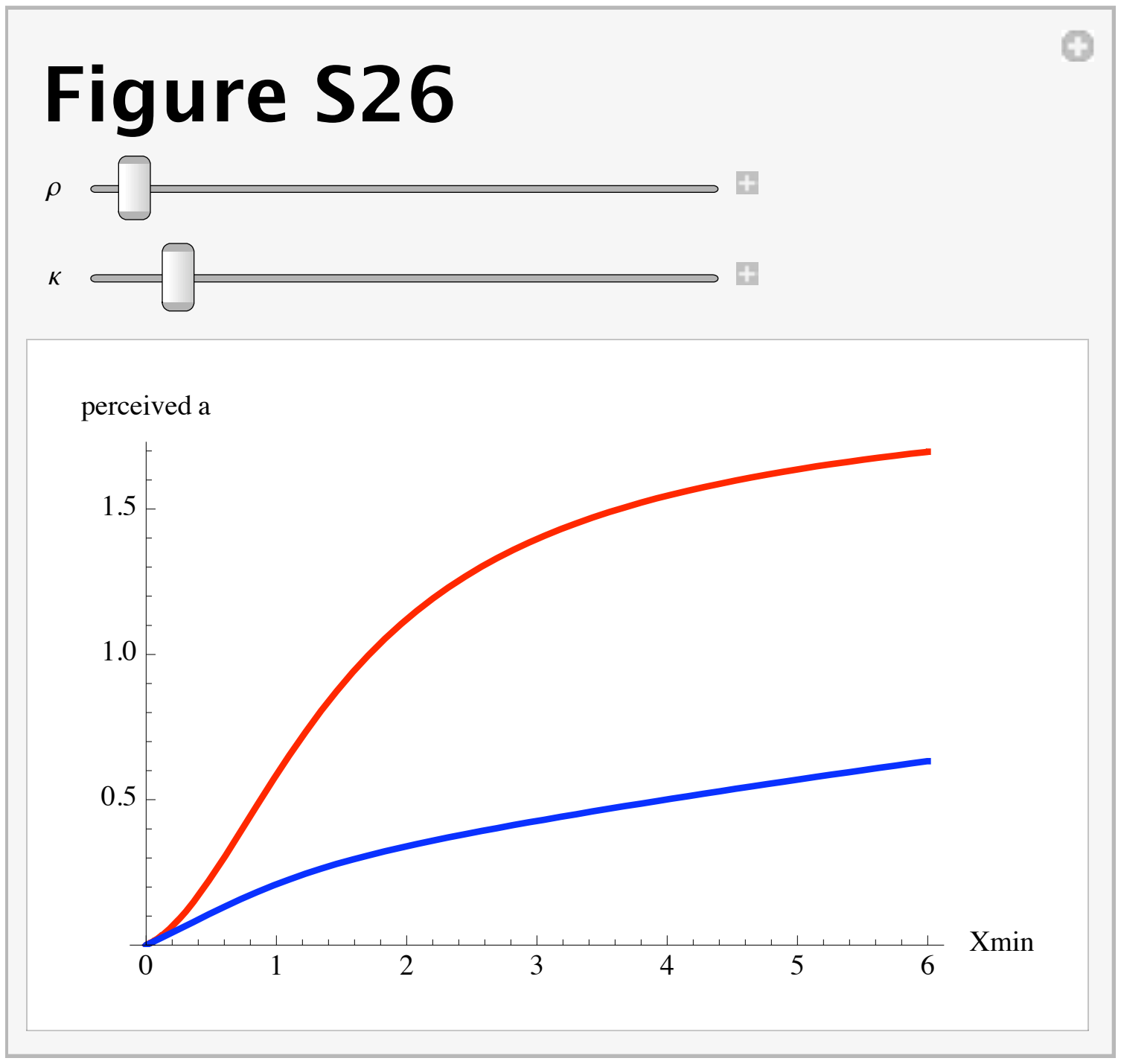

Below are two extreme cases: In Fig. 27, $\rho = 10$, which means one tenth as much degradation in the stroma as in the epithelium. In Fig. 28, $\rho = 0.001$, which means the stroma is virtually a perfect sink. In both cases, κ was 0.1, i.e. for the blue curve, concentration is only sensed over the bottom of the epithelium.

```
With[{term1 = αbar/ν, term2 = αkappa/ν}, Plot[
  Evaluate[{Tooltip[term1 /. ρ → 10, "αbar"], Tooltip[term2 /. {ρ → 10, κ → 0.1}, "αkappa"]}],
  {Xmin, 0, 6}, PlotRange → {0, Automatic}, PlotStyle → {{Thick, Red}, {Thick, Blue}},
  AxesLabel → {"Xmin", "perceived a"}, PlotLabel → Style["Figure S27", Bold, Large]]]
```

Figure S27

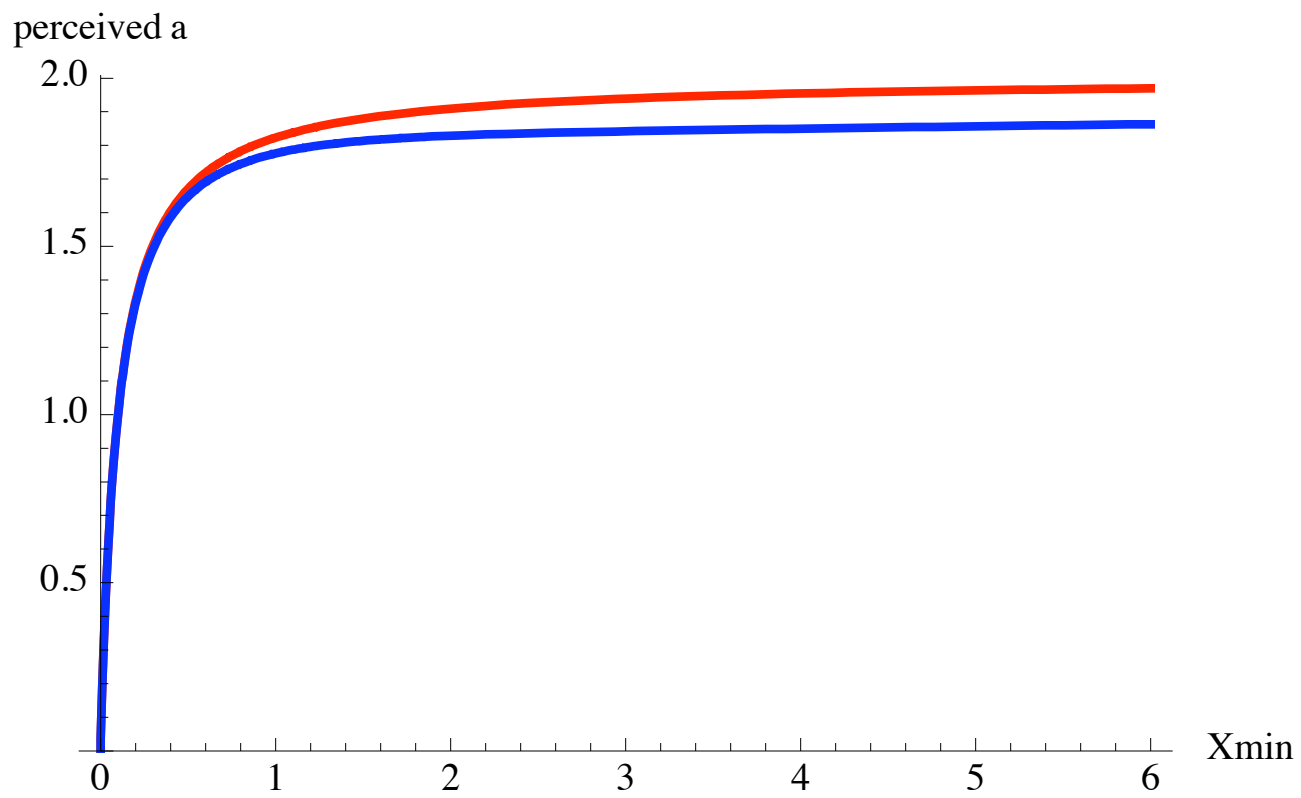


```
With[{term1 = αbar/ν, term2 = αkappa/ν}, Plot[Evaluate[
   {Tooltip[term1 /. ρ → 0.001, "αbar"], Tooltip[term2 /. {ρ → 0.001, κ → 0.1}, "αkappa"]}],
  {Xmin, 0, 6}, PlotRange → {0, Automatic}, PlotStyle → {{Thick, Red}, {Thick, Blue}},
  AxesLabel → {"Xmin", "perceived a"}, PlotLabel → Style["Figure S28", Bold, Large]]]
```

Figure S28

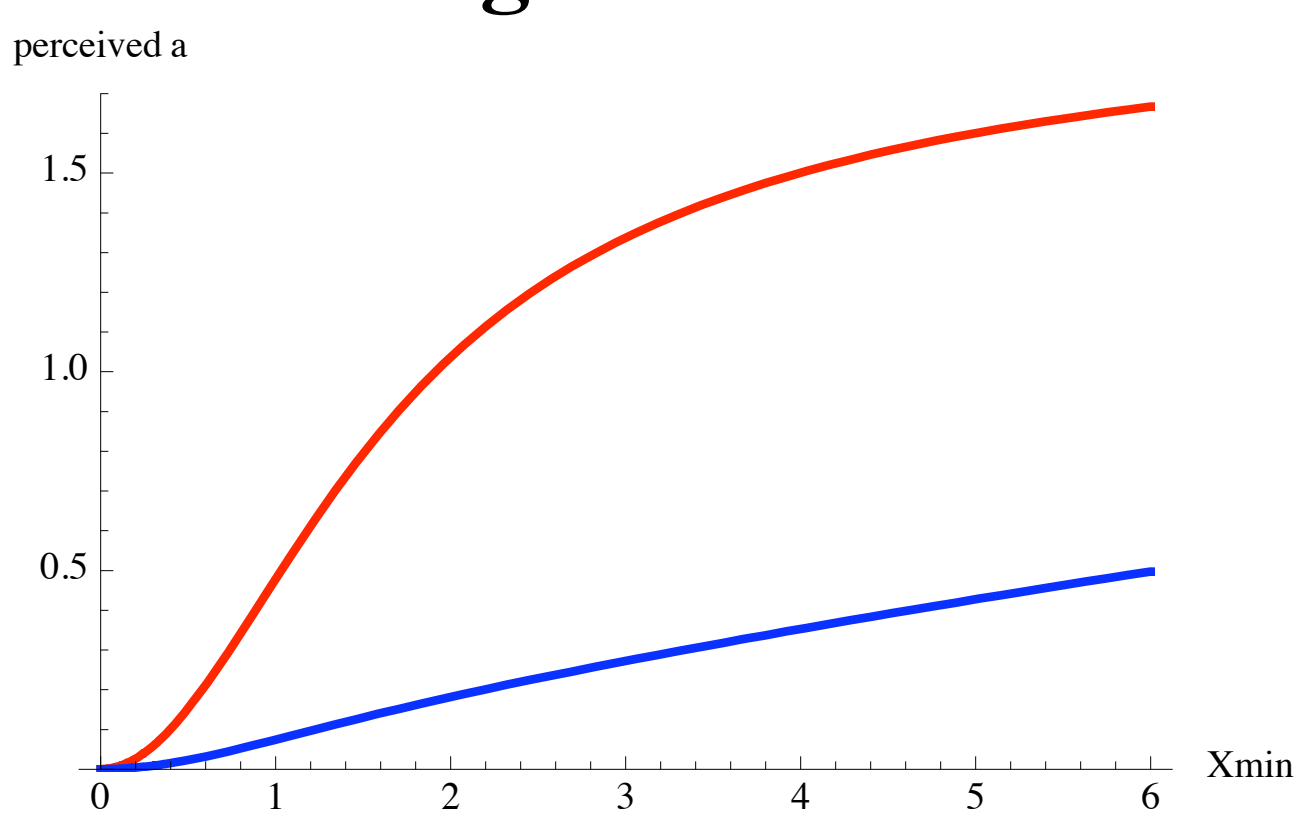

To be systematic, let's calculate the sensitivity of concentration to position, for these cases:

```
{senαbar, senαkappa} = {Sen[αbar, Xmin], Sen[αkappa, Xmin]} // FullSimplify
```

$$\left\{\frac{-2\,\text{Xmin} - \rho + \rho\,\text{Cosh}[2\,\text{Xmin}] + \text{Sinh}[2\,\text{Xmin}]}{2\,(\text{Cosh}[\text{Xmin}] + \rho\,\text{Sinh}[\text{Xmin}])\,(\text{Xmin}\,\text{Cosh}[\text{Xmin}] + (-1 + \text{Xmin}\,\rho)\,\text{Sinh}[\text{Xmin}])},\right.$$

$$(-2\,\text{Xmin} - \rho + \rho\,\text{Cosh}[2\,\text{Xmin}] - (\text{Xmin}\,\kappa + \rho)\,\text{Cosh}[\text{Xmin}\,(-2 + \kappa)] + (-\text{Xmin}\,(-2 + \kappa) + \rho)\,\text{Cosh}[\text{Xmin}\,\kappa] + \text{Sinh}[2\,\text{Xmin}] + \text{Sinh}[\text{Xmin}\,(-2 + \kappa)] + \text{Sinh}[\text{Xmin}\,\kappa] + \text{Xmin}\,\rho\,(\kappa\,\text{Sinh}[\text{Xmin}\,(-2 + \kappa)] - (-2 + \kappa)\,\text{Sinh}[\text{Xmin}\,\kappa]))\,/\,(2\,(\text{Cosh}[\text{Xmin}] + \rho\,\text{Sinh}[\text{Xmin}])$$

$$\left.(\text{Xmin}\,\kappa\,\text{Cosh}[\text{Xmin}] + (-1 + \text{Xmin}\,\kappa\,\rho)\,\text{Sinh}[\text{Xmin}] + \text{Sinh}[\text{Xmin} - \text{Xmin}\,\kappa]))\right\}$$

Let's look at the same two scenarios for ρ:

```
With[{term1 = senαbar, term2 = senαkappa}, Plot[Evaluate[
   {Tooltip[term1 /. ρ → 10, "senαbar"], Tooltip[term2 /. {ρ → 10, κ → 0.1}, "senαkappa"]}],
  {Xmin, 0, 2}, PlotRange → All, PlotStyle → {{Thick, Red}, {Thick, Blue}},
  AxesLabel → {"Xmin", "Sensitivity to Xmin"}, PlotLabel → Style["Figure S29", Bold, Large]]]
```

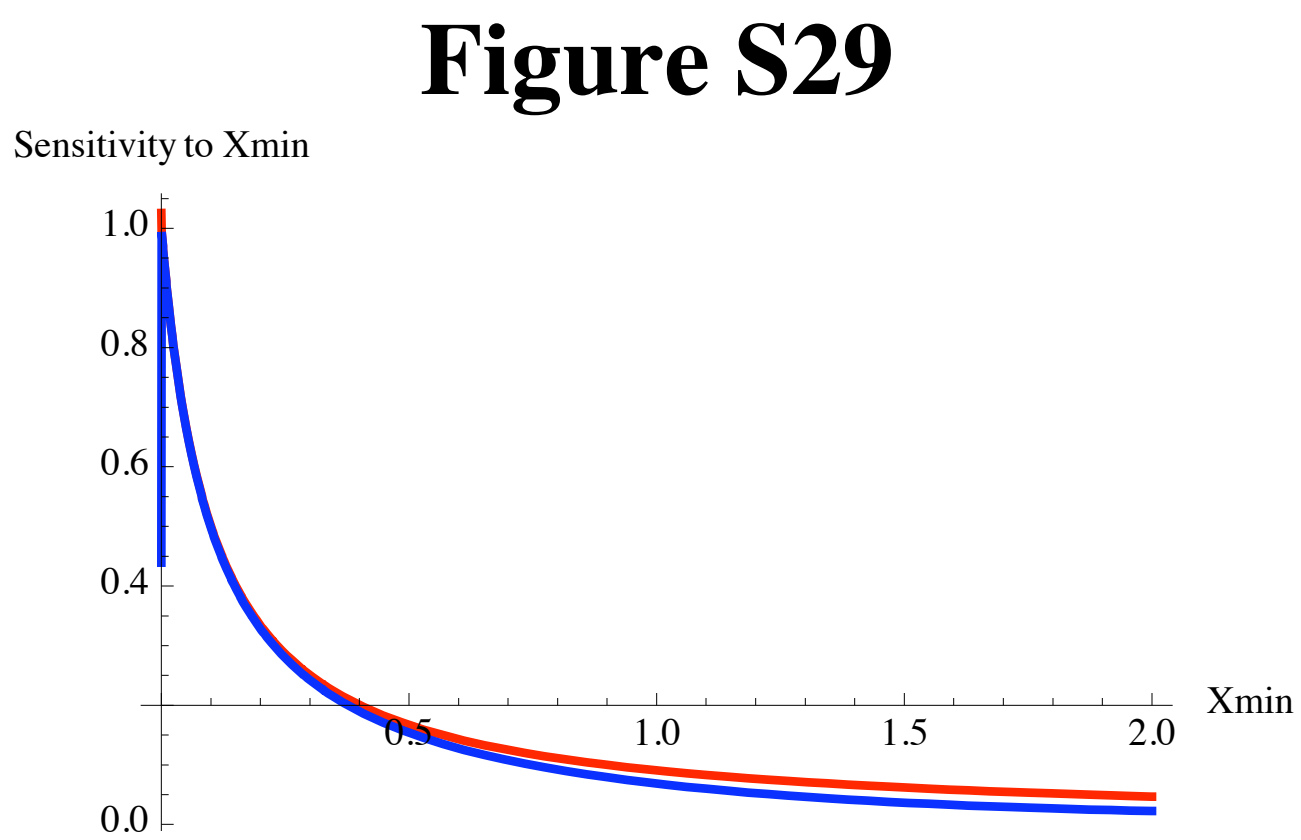

Clearly, in this case (high ρ) the epithelium's sensitivity to its own size falls below 0.5 when it is less than 0.2 diffusion lengths thick

```
With[{term1 = senαbar, term2 = senαkappa},
 Plot[Evaluate[{Tooltip[term1 /. ρ → 0.001, "senαbar"],
    Tooltip[term2 /. {ρ → 0.001, κ → 0.1}, "senαkappa"]}],
  {Xmin, 0, 6}, PlotRange → {0, 2}, PlotStyle → {{Thick, Red}, {Thick, Blue}},
  AxesLabel → {"Xmin", "Sensitivity to Xmin"}, PlotLabel → Style["Figure S30", Bold, Large]]]
```

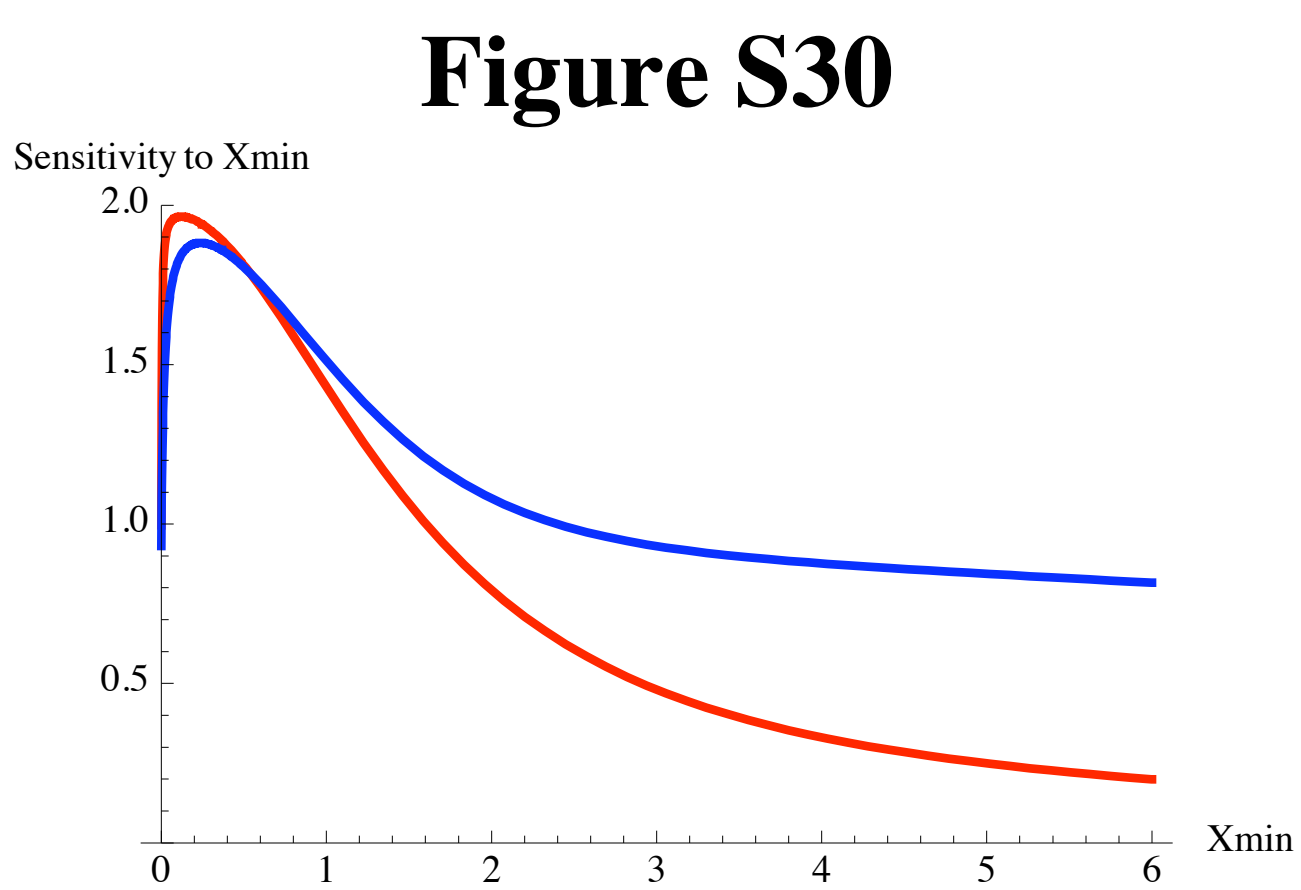

Remarkably, when the stroma is a good sink (low ρ), if the concentration is averaged all over the epithelium, sensitivity falls below 0.5 at a little over 2 diffusion lengths. But if concentration is averaged over the first 10% of the epithelium, then sensitivity remains close to 1 for very large values of epithelial thickness (up to 20 diffusion lengths [data not shown].

To plot the cartoons of the epithelium in Figure 7, the following code was used for each picture. The function "epitheliumplotBW" is defined, with a first argument representing the epithelial thickness in diffusion lengths, and the second representing the value of ρ. The apicobasal axis is renamed "Y" here so that the graphs plot in the right orientation. The basal surface appears at the top in the raw image.

```
epitheliumplotBW[height_, j_] :=
 With[{ν = 1, Ymin = height, ρ = j, frameheight = 10}, DensityPlot[2 ν - (2 ν Cosh[Y + Ymin])/(Cosh[Ymin] + ρ Sinh[Ymin]),
   {x, -10, 10}, {Y, 0, -Ymin}, Frame → False, AspectRatio → Ymin / 10,
   ColorFunction → Function[{x, y, z}, RGBColor[1 - z/2, 1 - z/2, 1 - z/2]],
   ColorFunctionScaling → False]]
```

For example:

```
GraphicsRow[{epitheliumplotBW[4, 0.001], epitheliumplotBW[4, 10]},
 PlotLabel → Style["Figure S31", Bold, Large]]
```

Figure S31

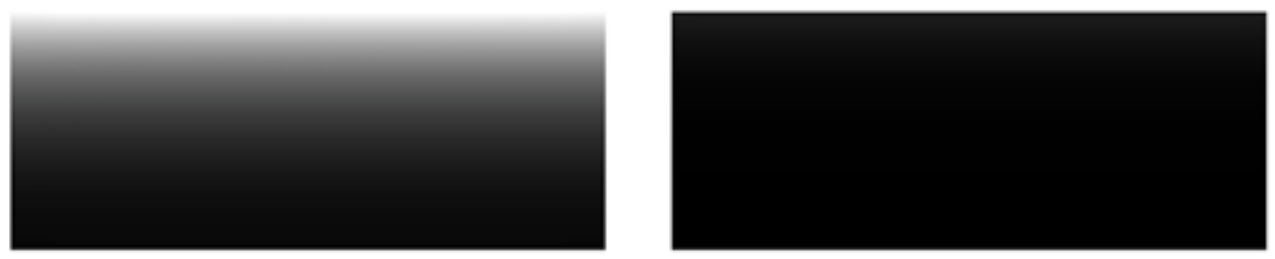

12. Parameters: definitions, ranges and justifications

For ODE (nonspatial) calculations

v_i, rate constant of division for cell type i, related to cell cycle length λ_i by $v_i = \ln 2\ \lambda_i$
p_i, probability of replication (as opposed to differentition) of cell type i
d, rate constant of death (or shedding) of terminal stage cell type
δ, non-dimensionalized death rate constant; $\delta = d / v_1$
ζ, ratio of v_0 to v_1
τ, non-dimensionalized time; $\tau = t\, v_1$
χ_i, number of cells of type i
c_i, number of cells of type i relative to number of cells of type 0
z_i, number of cells of type i relative to the steady state value for cells of type i
g, feedback gain for feedback of terminal cell stage onto p_1
γ, non-dimensionalized feedback gain, $g\,\chi_0$ (used when χ_0 is constant)
φ, non-dimensionalized feedback gain, $g\,\chi_{init}$ (used when χ_0 is not a constant)
h, feedback gain for feedback of terminal cell stage onto v_1
η, non-dimensionalized feedback gain, $h\,\chi_0$
j, feedback gain for feedback of terminal cell stage onto p_0
k, feedback gain for feedback of terminal cell stage onto v_0
ω, lumped, nondimensional parameter $g\, v_0\, \chi_0 / d$
a, amplification factor, equal to number of terminal stage cells divided by number of progenitors

For spatial dynamics calculations

a[x], concentration of molecule "a" at point x in space
αbar, concentration of molecule "a" averaged over epithelial thickness
αkappa, concentration of molecule "a" averaged over a basal region of the epithelium of thickness κ times the epithelial thickness
v, rate of production of molecule "a", at any point in space, in units of concentration per time
ν, non-dimensionalized production rate, $\nu = \frac{v}{2\, d_L\, \lambda_L^2} = \frac{v}{2\, k_L}$
d_L, effecitve diffusion coefficient of "a" in epithelium
d_R, effecitve diffusion coefficient of "a" in stroma
k_L, effective degradation rate constant of "a" in epithelium
k_R, effective degradation rate constant of "a" in stroma
λ_L, diffusion length constant of "a" in epithelium $= \sqrt{k_L / d}$; inversely related to diffusion length
λ_R, diffusion length constant of "a" in epithelium $= \sqrt{k_R / d}$; inversely related to diffusion length
ρ, ratio of diffusion length constants λ_L / λ_R;
thus ρ = ratio of diffusion length in R to diffusion length in L. If diffusion is the same in both compartments, then $\rho = \sqrt{k_L / k_R}$
[-xmin, 0], spatial domain of the epithelium;
[-Xmin, 0],
non-dimensionalized spatial domain of the epithelium (spatial units of diffusion length in epithelium)
[0, ∞], spatial domain of the stroma;

Parameter ranges used in simulations

Parameter	Range	Sampling
p_i	0 to 1	Linear
δ	$10^{-2.5}$ to $10^{-0.5}$	Logarithmic
ζ	0.1 to 1	Logarithmic
g	0.01 to 10	Logarithmic
γ	10^{-5} to 1	Logarithmic
h	0.01 to 10	Logarithmic
η	0.01 to 10	Logarithmic
j	0.01 to 10	Logarithmic
k	0.01 to 10	Logarithmic

Parameter range justifications

p_i : Probabilities;
δ : Turnover time for terminal stage cells wil vary between 3 to 316 cell cycle lengths for cell type 1. If a cell cycle length is 18 hours, then this means 2.4 days to about 8 months;
ζ : Cell cycle of cell type 1 varies from 10 times faster to 10 times slower than than of the stem cell;
g, h, j, k : Ranges largely empirical to give coverage of regimes will Hill functions are near - linear as well as near - saturated
γ, η : Ranges empirical, covering ranges of g $\chi_0[\infty]$ and h $\chi_0[\infty]$ that are observed in the outputs of simulations that use g and h
