# Supplementary material for: Cell Lineages and the Logic of Proliferative Control
Source: PLoS Biol. 2009 Jan 20;7(1):e1000015. doi: 10.1371/journal.pbio.1000015 (PMC2628408; doi:10.1371/journal.pbio.1000015)
Supplement: Supplementary file 2 [file pbio.1000015.st001.doc]

| Activin- and TGFβ-type | |
| --- | --- |
| GDF8/Myostatin | Striated muscle [1,2] |
| GDF11 | Olfactory epithelium [3] |
| Activins | Olfactory epithelium [4]  Liver [5]  Renal tubular epithelium [6] |
| BMP3 | Bone [7] |
| TGFβ | Liver [8]  Retina [9]  Epidermis [10]  Fibroblasts [11] |
| BMP-type | |
| BMP2 | Hair follicles [12,13] |
| *Others:* | |
| IL-1 | Hepatocytes [14] |
| Wnts | B-lympocytes [15] |
| Oncostatin M | Hair follicles [16] |
| Vasohibin | Vascular endothelial cells [17 ] |
| VEGI/TNFSF15 | Vascular endothelial cells [18] |
| Platelet factor 4 | Megakaryocytes [19] |
| Glutamate, GABA | Central nervous system [20,21] |
| N-substituted oligopeptides | Many tissues [22] |
| Various factors (chemokines, cytokines, oligopeptides) | Hematopoietic cells [23,24] |

**Supplemental Table 1.** Negative feedback regulators of proliferation. Citations refer to references below:

References

1. McPherron AC, Lawler AM, Lee SJ (1997) Regulation of skeletal muscle mass in mice by a new TGF-beta superfamily member. Nature 387: 83-90.

2. McKoy G, Bicknell KA, Patel K, Brooks G (2007) Developmental expression of myostatin in cardiomyocytes and its effect on foetal and neonatal rat cardiomyocyte proliferation. Cardiovasc Res 74: 304-312.

3. Wu HH, Ivkovic S, Murray RC, Jaramillo S, Lyons KM, et al. (2003) Autoregulation of neurogenesis by GDF11. Neuron 37: 197-207.

4. Gokoffski KK, Wu H-H, Beites CL, Kim JS, Silman E, et al. (2008) Differential Actions of Activin and GDF11 On Proliferation and Fate Choice By Neural Stem/Progenitor Cells. *Submitted*.

5. Endo D, Maku-Uchi M, Kojima I (2006) Activin or follistatin: which is more beneficial to support liver regeneration after massive hepatectomy? Endocr J 53: 73-78.

6. Maeshima A, Nojima Y, Kojima I (2002) Activin A: an autocrine regulator of cell growth and differentiation in renal proximal tubular cells. Kidney Int 62: 446-454.

7. Daluiski A, Engstrand T, Bahamonde ME, Gamer LW, Agius E, et al. (2001) Bone morphogenetic protein-3 is a negative regulator of bone density. Nat Genet 27: 84-88.

8. Strain AJ (1992) Transforming growth factor-beta: the elusive hepatic chalone? Hepatology 16: 269-270.

9. Close JL, Gumuscu B, Reh TA (2005) Retinal neurons regulate proliferation of postnatal progenitors and Muller glia in the rat retina via TGF beta signaling. Development 132: 3015-3026.

10. Yamasaki K, Toriu N, Hanakawa Y, Shirakata Y, Sayama K, et al. (2003) Keratinocyte growth inhibition by high-dose epidermal growth factor is mediated by transforming growth factor beta autoinduction: a negative feedback mechanism for keratinocyte growth. J Invest Dermatol 120: 1030-1037.

11. Sudarshan C, Yaswen L, Kulkarni A, Raghow R (1998) Phenotypic consequences of transforming growth factor beta1 gene ablation in murine embryonic fibroblasts: autocrine control of cell proliferation and extracellular matrix biosynthesis. J Cell Physiol 176: 67-75.

12. Plikus MV, Mayer JA, de la Cruz D, Baker RE, Maini PK, et al. (2008) Cyclic dermal BMP signalling regulates stem cell activation during hair regeneration. Nature 451: 340-344.

13. Kobielak K, Stokes N, de la Cruz J, Polak L, Fuchs E (2007) Loss of a quiescent niche but not follicle stem cells in the absence of bone morphogenetic protein signaling. Proc Natl Acad Sci U S A 104: 10063-10068.

14. Boulton R, Woodman A, Calnan D, Selden C, Tam F, et al. (1997) Nonparenchymal cells from regenerating rat liver generate interleukin-1alpha and -1beta: a mechanism of negative regulation of hepatocyte proliferation. Hepatology 26: 49-58.

15. Dosen G, Tenstad E, Nygren MK, Stubberud H, Funderud S, et al. (2006) Wnt expression and canonical Wnt signaling in human bone marrow B lymphopoiesis. BMC Immunol 7: 13.

16. Yu M, Kissling S, Freyschmidt-Paul P, Hoffmann R, Shapiro J, et al. (2008) Interleukin-6 cytokine family member oncostatin M is a hair-follicle-expressed factor with hair growth inhibitory properties. Exp Dermatol 17: 12-19.

17. Watanabe K, Hasegawa Y, Yamashita H, Shimizu K, Ding Y, et al. (2004) Vasohibin as an endothelium-derived negative feedback regulator of angiogenesis. J Clin Invest 114: 898-907.

18. Metheny-Barlow LJ, Li LY (2006) Vascular endothelial growth inhibitor (VEGI), an endogenous negative regulator of angiogenesis. Semin Ophthalmol 21: 49-58.

19. Lambert MP, Rauova L, Bailey M, Sola-Visner MC, Kowalska MA, et al. (2007) Platelet factor 4 is a negative autocrine in vivo regulator of megakaryopoiesis: clinical and therapeutic implications. Blood 110: 1153-1160.

20. Platel JC, Dave KA, Bordey A (2008) Control of neuroblast production and migration by converging GABA and glutamate signals in the postnatal forebrain. J Physiol.

21. Platel JC, Lacar B, Bordey A (2007) GABA and glutamate signaling: homeostatic control of adult forebrain neurogenesis. J Mol Histol 38: 602-610.

22. Elgjo K, Reichelt KL (2004) Chalones: from aqueous extracts to oligopeptides. Cell Cycle 3: 1208-1211.

23. Marshall E, Lord BI (1996) Feedback inhibitors in normal and tumor tissues. Int Rev Cytol 167: 185-261.

24. Lord BI (1988) Feedback regulators in normal and tumour tissues. J Cell Sci Suppl 10: 231-242.
